# Ligand‐driven modulation of chaperone–cochaperone networks shapes proteostasis outcomes

**DOI:** 10.1002/pro.70543

**Published:** 2026-04-08

**Authors:** Andrea Magni, Giorgio Bonollo, Gauthier Trèves, Francesco Frigerio, Fabrizio Cinquini, Silvia Pavoni, A. Sofia F. Oliveira, Adrian J. Mulholland, Stefano A. Serapian, Giorgio Colombo

**Affiliations:** ^1^ Dipartimento di Chimica Università di Pavia Pavia Italy; ^2^ Department of Physical Chemistry R&D Eni SpA San Donato Milanese (Mi) Italy; ^3^ Centre for Computational Chemistry School of Chemistry, University of Bristol Bristol UK

**Keywords:** allostery, chaperones, Hsp90, internal dynamics, ligand recognition, ligand regulation of function, molecular dynamics, protein folding

## Abstract

Protein homeostasis depends on a delicate interplay between folding and degradation, orchestrated by molecular chaperones. Among them, Hsp90 is a central hub, regulating nearly 10% of the proteome through ATP‐driven conformational cycles and selective interactions with cochaperones. The glucocorticoid receptor (GR) represents a paradigmatic Hsp90 client, whose maturation requires sequential remodeling steps involving multi‐protein assemblies. While cryo‐EM provided snapshots of these complexes, the dynamic determinants of GR activation and the antagonistic roles of cochaperones FKBP51 and FKBP52 remain poorly understood. Here, we integrate unbiased equilibrium atomistic molecular dynamics with nonequilibrium simulations of four different Hsp90‐cochaperone‐client assemblies that oversee distinct steps of GR maturation to elucidate how finely tuned dynamics and coordination/communication mechanisms determine functional emergence. Perturbations encoded by ligand insertion or removal reveal steroid binding as critical for both structural stability and inter‐component communication. Ligand engagement not only stabilizes GR's active conformation but also feeds back to reshape chaperone and cochaperone dynamics, thereby modulating progression through the folding pathway. Steroid binding reinforces the interface in the Hsp90–p23–GR assembly, positioning cochaperone p23 as a molecular sensor for ligand occupancy. Comparative analyses of post‐maturation complexes further uncover how immunophilins FKBP51 and FKBP52, despite structural similarity, elicit divergent allosteric effects on GR conformation and Hsp90–ATPase, determining opposing client fates. Our results establish ligand binding as an active modulator of chaperone‐mediated folding, linking metabolic cues (ligand presence and levels) to client maturation. More broadly, they highlight cochaperones as dynamic checkpoints that selectively bias client outcomes, revealing generalizable principles of proteostasis regulation and opportunities for therapeutic intervention.

## 1 INTRODUCTION

Cells operate as highly dynamic systems, where a dense and intricate network of molecular events takes place continuously (Bunne et al., 2024; Goldberg et al., 2018). At the core of these processes lie proteins, the versatile macromolecules that drive nearly every aspect of cellular physiology. To function, newly synthesized polypeptides must acquire their native three‐dimensional conformations, while defective, misfolded, or no‐longer‐needed proteins must be efficiently cleared (Gershenson & Gierasch, 2011; Mecha et al., 2022; Rodina et al., 2023). The equilibrium between folding and degradation is therefore central to proteome integrity, which requires strict regulation of protein homeostasis (Lim & Vendruscolo, 2025; Miles et al., 2019).

Molecular chaperones have long been recognized as indispensable guardians of this balance, ensuring that client proteins achieve their functional states (Bhattacharya & Picard, 2021; Brehme et al., 2014; Ciechanover & Kwon, 2017; Hartl, 1996; Hartl et al., 2011; Kim et al., 2013). More recently, however, evidence has revealed that chaperones also act as triaging agents targeting unstable or damaged clients for degradation, thereby linking distinct aspects of cellular protein quality control pathways (Chio et al., 2025).

Among others, Heat Shock Protein 90 kDa (Hsp90) has taken up a unique role in the molecular chaperone family, as it acts late in the folding pathway of clients and its dysregulation has been associated with several diseases, ranging from cancer to neurodegeneration (Chiosis et al., 2023; Kurop et al., 2021; Schopf et al., 2017; Silbermann et al., 2024; Truman et al., 2021).

**FIGURE 1:**
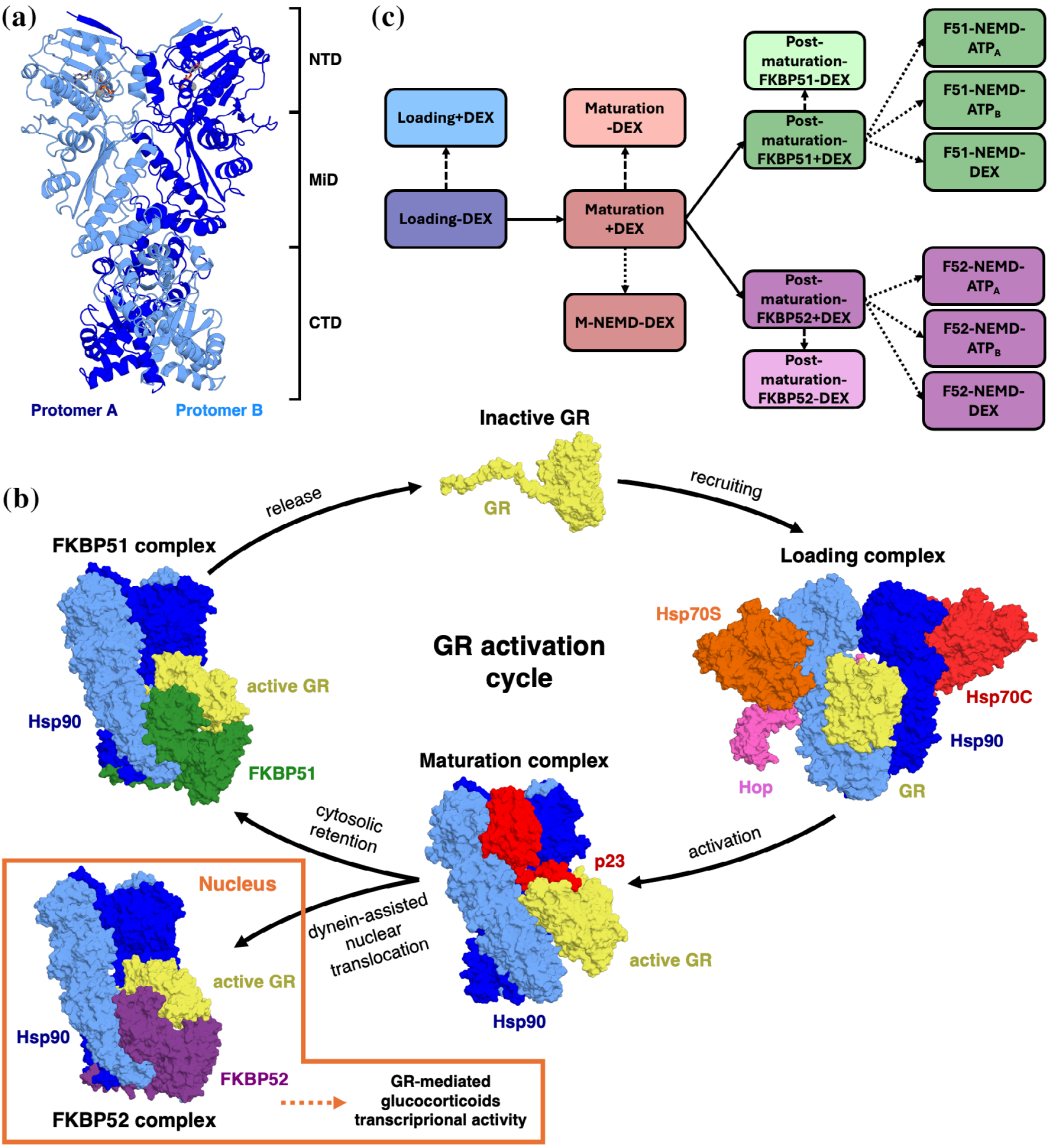
Structural organization and chaperone cycle of the Hsp90 machinery. (a) The structural organization of Hsp90: Protomer A in blue; protomer B in light blue. (b) Schematic representation of the GR activation cycle, showing Cryo‐EM resolved structures of multiprotein complexes at different stages. Individual proteins are defined in the main text. (c) A schematic view of the different equilibrium and non‐equilibrium simulations performed for this study.

Hsp90 is a homodimeric protein, composed of two identical protomers (protomer A and protomer B), each subdivided into three domains (Ali et al., 2006; Dollins et al., 2007; Lavery et al., 2014; Pearl & Prodromou, 2006; Sahasrabudhe et al., 2017; Schopf et al., 2017; Shiau et al., 2006). In the N‐terminal domains (NTD), where the active site is located, ATPase activity takes place via an asymmetric mechanism, involving a first hydrolysis at one protomer followed by a second hydrolysis at the other. The middle domain (MiD) binds client proteins. Finally, the C‐terminal domain (CTD) is responsible for preserving dimerization and is also involved in client recognition (Figure 1a). Several Hsp90 isoforms exist in different cell compartments (Hsp90⍺ and Hsp90β in the cytoplasm, Grp94 in the endoplasmic reticulum, TRAP1 in mitochondria), and their clients and functional networks do not overlap (Albakova et al., 2022; Chiosis et al., 2023; Guarra et al., 2024; Kolhe et al., 2023; Qu et al., 2024; Truman et al., 2021). Notably, Hsp90 oversees the quality control of about 10% of the proteome (Finka & Goloubinoff, 2013), and has a long interactors list that is constantly being updated (Chiosis et al., 2023; Echeverria et al., 2012).

Due to the wide variety of its clientome, Hsp90's context‐dependent activity is finely tuned by the association with other task‐specific proteins, called cochaperones, that selectively modulate the dynamics of chaperone‐client interactions. As mentioned, the chaperoning role of Hsp90 is achieved through an ATP‐driven conformational cycle that involves structural changes in the chaperone itself and the coordinated assembly and disassembly of various multi‐protein complexes, which ultimately determine selective client remodeling and activation. In the Hsp90 chaperone cycle, ATP binds to both protomers, shifting the population to a closed high‐energy state characterized by an asymmetric organization. Hydrolysis of the first ATP leads to a change in symmetry with transition to a symmetric closed dimer reminiscent of the initial yHsp90 crystal structure. In this scenario, the conformational change of the chaperone implies a significant remodeling of the client binding site, defining a direct coupling between the two main functional sites in Hsp90. This also facilitates the induction of structural changes in the client. Hydrolysis of the second ATP drives the chaperone into a transiently compact ADP‐bound state, from which client proteins are released, before nucleotide dissociation and the return of the chaperone to the initial state (Lavery et al., 2014).

Biochemical and biophysical investigations, together with advances in cryo‐EM structural analysis, have provided high‐resolution details of the complexes that control the mechanisms in the key stages of glucocorticoid receptor (GR) activation (Dahiya et al., 2022; Kirschke et al., 2014; Li et al., 2013; Lopez et al., 2021; Lorenz et al., 2014; Noddings et al., 2022; Sabbagh et al., 2018; Wang et al., 2022).

GR mediates the action of glucocorticoids in the cell, a class of hormones whose altered cellular levels are linked to Cushing's and Addison's disease (Kadmiel & Cidlowski, 2013). This receptor consists of three domains, but only experimental structures for the ligand‐binding domain (LBD) and part of the inter‐domain connecting loop are currently available in the Protein Data Bank. GR's functional maturation is orchestrated by Hsp90 through a stepwise cycle that transitions the client from an inhibited, unfolded state to its active, ligand‐bound conformation (Figure 1b). Initially, the nascent GR is recruited and stabilized by Hsp40 and Hsp70, before being transferred to Hsp90 with the aid of Hop and a second Hsp70 to form the “Loading complex” (Wang et al., 2022). In this intermediate state, Hsp90 adopts a semi‐closed conformation, clamping the receptor's disordered tail within its central lumen while GR remains inactive and partially unfolded. The two Hsp70 molecules play distinct roles, one acting as a scaffold and the other directly engaging with the GR N‐terminal tail. Dissociation of Hsp70 and Hop drives the transition to the “Maturation complex” (Noddings et al., 2022), marked by incorporation of cochaperone p23. Here, Hsp90 achieves a fully closed, ATP‐hydrolysis–competent state that enables remodeling of the GR ligand‐binding domain, resulting in receptor activation, as exemplified by cryo‐EM structures showing the engagement of GR ligand, dexamethasone (DEX).

Subsequent late‐stage remodeling is mediated by mutually exclusive interactions with the immunophilins FKBP51 and FKBP52 (Noddings et al., 2023), which displace p23 and bind to the Hsp90 C‐terminal region while contacting GR near its active site (Figure 1b). Despite adopting nearly identical poses, these cofactors encode opposing functional outcomes. FKBP52 tightens GR binding to the complex and promotes dynein‐mediated nuclear translocation of the entire multiprotein assembly, enhancing glucocorticoid signaling. Indeed, to successfully mediate transcriptional activity, GR needs to reach the cellular nucleus while preserving association with the Hsp90 machinery to remain protected in the active form. In contrast, FKBP51 acts as an antagonist and constrains GR in the cytosol, facilitating receptor release, spontaneous deactivation and subsequent recycling (Noddings et al., 2023). Intriguingly, in both immunophilin‐bound complexes, GR remains ligand‐bound and competent for signaling, underscoring the finely tuned balance of chaperone‐cochaperone interactions and suggesting a role for the receptor ligand in dictating GR maturation and release.

Despite exciting advancements in the structural characterization of complexes along the pathway to GR folding, many open questions remain regarding the detailed mechanisms of their functional regulation and their relationships to GR maturation. These range from the functional role of dynamic cross‐talk between components of the assemblies to GR organization within different complexes, including the link between GR substrate binding and the determinants that underlie the distinct behaviors of the highly similar immunophilins FKBP51 and FKBP52.

Here, we address these fundamental questions using unbiased equilibrium and out‐of‐equilibrium atomistic molecular dynamics (MD) simulations. Our aim is to shed light on the fine determinants of the dynamics of the different complexes and models of GR‐ligand states, reconnecting them to the observed functional roles of the chaperone complexes in regulating the client folding pathways. Specifically, by integrating extensive equilibrium MD with dynamical‐nonequilibrium MD (D‐NEMD) simulations and advanced analysis approaches (Artur et al., 2023; Balega et al., 2025; Castelli, Magni, et al., 2024; Castelli, Marchetti, et al., 2024; Meli et al., 2020; Morra et al., 2012; Oliveira et al., 2021; Oliveira et al., 2022; Oliveira, Edsall, et al., 2019; Oliveira, Shoemark, et al., 2019; Serapian et al., 2020), we uncover how internal dynamics and inter‐component communication shape progression through the chaperone cycle (Figure 1c). In brief, our results show how the chaperone machinery primes GR for steroid binding, while also suggesting that the steroid can act as a signaling molecule whose engagement feeds back to modulate the stability and dynamics of the various assemblies. Collectively, these findings establish a direct link between the atomic‐scale conformational dynamics of GR‐chaperone assemblies and specific chemical entities in the binding sites, thereby defining how molecular inputs are translated into distinct modulations of the complexes that oversee the folding of Hsp90 client proteins. Our results reveal long‐range allosteric effects triggered by subtle structural modifications—whether encoded by the nucleotide state of the chaperone or by steroid binding/unbinding in the client—that propagate across proteins and domains to influence and regulate function.

## 2 MATERIALS AND METHODS

### 2.1 Systems modeling and preparation

The models and simulation names with related labels are reported in Table 1.

**TABLE 1:** On the top, a summary of the equilibrium classical MD models and simulations featured in this work. On the bottom, a summary of the non‐equilibrium simulations (D‐NEMD).

Equilibrium simulations (classical MD)					
Model	Components	Unperturbed/perturbed	PDB ID	MD name	Length (μs)
Loading‐DEX	Hsp90:Hsp70C_ADP_:Hsp70S_ADP_:Hop:GR	Unperturbed	7KRW	L‐DEX	4
Loading + DEX	Hsp90:Hsp70C_ADP_:Hsp70S_ADP_:Hop:GR_DEX_	Perturbed	7KRW	L + DEX	5
Maturation + DEX	Hsp90_ATP_:p23:GR_DEX_	Unperturbed	7KRJ	M + DEX	4
Maturation‐DEX	Hsp90_ATP_:p23:GR	Perturbed	7KRJ	L‐DEX	5
Post‐maturation‐FKBP51 + DEX	Hsp90:FKBP51:GR_DEX_	Unperturbed	8FFW	F51 + DEX	9
Post‐maturation‐FKBP51‐DEX	Hsp90:FKBP51:GR	Perturbed	8FFW	F51‐DEX	5
Post‐maturation‐FKBP51 + DEX	Hsp90:FKBP52:GR_DEX_	Unperturbed	8FFV	F52 + DEX	9
Post‐maturation‐FKBP51‐DEX	Hsp90:FKBP52:GR	Perturbed	8FFV	F52‐DEX	5

Non‐equilibrium simulations (D‐NEMD)		
Starting model	D‐NEMD name	Perturbation
Maturation + DEX	M‐NEMD‐ATP M‐NEMD‐DEX	ATP hydrolysis DEX removal
Post‐maturation‐FKBP51 + DEX	F51‐NEMD‐ATP F51‐NEMD‐DEX	ATP hydrolysis DEX removal
Post‐maturation‐FKBP52 + DEX	F52‐NEMD‐ATP F52‐NEMD‐DEX	ATP hydrolysis DEX removal

#### 2.1.1 Loading‐DEX and loading + DEX complexes

Loading‐DEX complex modeling starts from the cryo‐EM structure of PDB 7KW7 (Wang et al., 2022). The Hsp90α sequence is based on UniProt P07900 and includes residues from E16 to D699. Due to its high flexibility, the charged linker (residues 233–283) is not resolved in the original structure of either protomers and was thus modeled as a shorter version to preserve backbone continuity within each protomer. In detail, we connected the two protein portions with an EKEK loop using the *Crosslink Proteins* utility of the *Maestro Schrödinger suite* version 2021–4 (*Schrödinger Release 2021–4: Maestro, Schrödinger, LLC, New York, NY, 2021*), following the same procedure as in our previous work (Castelli et al., 2023). Regarding the presence of ligands, Hsp90 is in the *apo* form, but both instances of Hsp70 (scaffolding Hsp70S and catalytic Hsp70C) feature one Mg^2+^ cation and one ADP nucleotide inside their active sites.

The Loading + DEX complex is based on the Loading‐DEX complex, but with the ligand DEX docked into the active site of GR. Induced‐fit docking (IFD) was performed to accommodate the ligand in the otherwise closed binding pocket of inactive GR. In detail, we used the 4 μs‐long unbiased MD simulations of both Loading‐DEX and Maturation + DEX (vide infra) complexes to obtain conformations of the Loading‐DEX complex suitable for the IFD procedure. For this, for each frame of the Loading‐DEX complex trajectories, we computed the root mean square deviation (RMSD) against each frame of the Maturation + DEX complex trajectories, considering only the GR folded core (residues 559 to 769), thus excluding the long tail loop. Some of the Loading‐DEX frames achieved low RMSD values (around 1.7 Å) for the GR core backbone. From the resulting 2D *n* x *n* RMSD matrix, we extract the conformations of the Loading‐DEX complex that better resemble the conformations of the Maturation + DEX (frame pairs with the lowest RMSD); therefore collecting GR conformations from the unbiased Loading‐DEX complex simulation, with an overall structure and an active site arrangement similar to the reference Maturation + DEX complex and that can accommodate the ligand in its wider binding pocket. A total of four suitable frames were selected for the IFD protocol, which was entirely performed using *Maestro Schrödinger suite* version 2024–3 (*Schrödinger Release 2024–3: Maestro, Schrödinger, LLC, New York, NY, 2024*). This procedure allowed the retrieval of GR conformations from the Loading‐DEX simulation that were structurally comparable to the active, ligand‐competent GR within Maturation + DEX MDs.

The four selected conformations of the Loading‐DEX identified were then prepared using *Maestro Protein Preparation Workflow* utility. The *Maestro LigPrep* tool was used to prepare the DEX ligand, for which the pH was set to 7.00 ± 2.00 using the *Epik* utility (Johnston et al., 2023). The IFD protocol for the Loading + DEX structure was then performed with *Maestro* (Sherman et al., 2006). The inner‐box size for the induced docking procedure was set as a 10 Å cube centered on the centroid of the residues within a 4 Å radius from the DEX ligand in the GR's Maturation + DEX complex model (namely M560, L563, N564, L566, G567, Q570, M601, M604, A605, L608, F623, Q642, M646, L732, Y735, C736, T739, I747, F749, and L753). However, some of the residue side chains around the defined pocket significantly reduced the available space for the docking, potentially clashing with DEX. To circumvent this, we trimmed their sidechains to Ala (and then reconstructed them) during the IFD process. This was done for six residues within the DEX binding site, namely L563, N564, L566, M604, L608, and Y735. After the IFD process, residues within 7.0 Å from each atom of ligand poses were minimized and their side chains optimized with *Prime* tool. In the final step, 20 poses from the IFD set created above were selected to be redocked, using *Maestro Glide* redocking in Standard Precision (SP) mode. The resulting poses were visually evaluated, and the five with the highest similarity to the DEX pose in the Maturation + DEX complex reference frame were selected for the equilibrium simulations (Figure S1).

#### 2.1.2 Maturation + DEX and maturation‐DEX complexes

The starting structure of the Maturation + DEX complex is based on the cryo‐EM PDB ID 7KRJ (Noddings et al., 2022), which contains cochaperone p23 and client GR (Figure 1b). As for the Loading‐DEX complex, the Hsp90 charged linker is unresolved in both protomers within the original PDB and was modeled as described above to ensure no discontinuities between N‐Terminal Domains (NTDs) and Middle Domains (MiDs).

In addition to the Hsp90 linker, in the cryo‐EM structure, GR lacks four residues in the N‐terminus (amino acids SIVP) compared to the Loading‐DEX complex. Such residues belong to the important GR tail that is threaded in the Hsp90 lumen between the Hsp90 MiDs of protomer A and B. Using the *PyMOL* molecular modeling package (Schrödinger, LLC, The *PyMOL* Molecular Graphics System, Version 1.82015), we reconstructed the missing amino acids (from residue 519 to 522 according to UniProt P04150) to obtain a complete structure of GR LBD from residue 519 to 577. There were no modifications involving cochaperone p23, which was left as experimentally resolved. Regarding the ligands, an ATP nucleotide molecule and a Mg^2+^ cation were present in each Hsp90 NTD, and the DEX ligand was bound to GR's active site.

The artificial Maturation‐DEX complex structure was obtained by simply removing DEX from the GR ligand‐binding domain.

#### 2.1.3 Post‐maturation‐FKBP51 + DEX and post‐maturation‐FKBP51‐DEX complexes

The Post‐maturation‐FKBP51 + DEX complex structure was based on the cryo‐EM structure PDB ID 8FFW (Noddings et al., 2023). As for the other models, the original cryo‐EM structure has the charged linker not fully resolved for both protomers. This linker was replaced with a smaller loop with sequence EKEK (as for the previous structures) by the *crosslink* tool of *Schrödinger Maestro* suite (Schrödinger release 2021–4, Schrödinger, LLC, New York, NY, 2021). Unlike the other PDBs, there are some residues belonging to the final portion of the CTD of protomer B (CTD_B_), which are in contact with the immunophilin FKBP51, that are resolved (residues 724–732). These residues are separated from the main CTD_B_ by a missing, unresolved loop. We modelled the missing region with the same *crosslink* tool, joining these two parts (residues 704–723 with sequence TADDTSAAVTEEMPPLEGDD). For GR, we introduced a single point mutation (T519S) in the Post‐maturation FKBP51 to restore a wild type (WT) serine. FKBP51 was retained as in the original cryo‐EM structure. This multi‐protein complex contains one ATP nucleotide molecule and a Mg^2+^ ion bound to each Hsp90's NTD and a DEX ligand in the GR's binding pocket.

The artificial Post‐maturation‐FKBP51‐DEX was prepared based on the above‐described modeled WT structure by simply removing the DEX ligand from its binding site on GR.

#### 2.1.4 Post‐maturation‐FKBP52 + DEX and post‐maturation‐FKBP52‐DEX complexes

The Post‐maturation‐FKBP52 + DEX model was built in the same way as the structure containing immunophilin FKBP51, but starting from the cryo‐EM structure PDB ID 8FFV (Noddings et al., 2023). All missing loops were modelled as described above. Also, the T519S mutation (back to WT) was introduced in GR, as done for the Post‐maturation‐FKBP51 + DEX complex.

Similarly, the Post‐maturation‐FKBP52‐DEX model was obtained by simply removing the DEX ligand from its active site.

#### 2.1.5 Preparation of the systems

All the models were pre‐processed for protonation and histidine tautomeric states with *Schrödinger Maestro* suite (Schrödinger release 2021–4, Schrödinger, LLC, New York, NY, 2021). The most likely protonation state for the titratable sites in the complexes at physiological pH was chosen based on *PropKa* predictions (Olsson et al., 2011). Histidine tautomerization was assigned by the H‐bond optimization tool during protein preparation. The charge state of ATP was considered to be −4. Starting structures and input files are available at the following link (https://doi.org/10.5281/zenodo.18267647).

All the simulations were carried out in explicit solvent, with each model solvated using *AmberTools*' *tleap* utility of the *Amber* suite in a periodic cuboidal box of TIP3P (Jorgensen et al., 1983) water molecules. To ensure adequate dimensions and avoid interactions between periodic images, the simulation boxes were built to have a minimum distance of 11 Å between any atom of the protein and the edge of the simulation box. Finally, the negative charge excess for each model was neutralized by randomly adding the corresponding amount of Na^+^ cations to the boxes.

#### 2.1.6 Equilibrium MD simulations

MD simulations of all modeled complexes employed the same parameters (i.e., used the same force‐field and simulation settings). The proteins were described using the *ff14SB* forcefield (Maier et al., 2015) and water using the *TIP3P* model (Jorgensen et al., 1983).

Regarding the ions, Joung and Cheatham parameters were assigned to Na^+^ cations (Joung & Cheatham, 2008), while Allnér and Nilsson ones were used for Mg^2+^ (Allnér et al., 2012). Nucleotides, ATP in Hsp90 and ADP in Hsp70 and Hsp90, were parameterized according to the polyphosphate parameters published by Meagher and coworkers (Meagher et al., 2003). DEX, which is present in the majority of the structures, was parametrized using quantum mechanical calculations to the assignment of point charges. Using the *Gaussian16* package (Frisch et al., 2016), DEX structure was optimized to an energy minimum in the gas phase using density functional theory (DFT) with B3LYP and 6‐31G(d) as functional and basis set, respectively. Next, *Gaussian16* was used to calculate ESP charges (Lee et al., 1988) at the Hartree‐Fock/6‐31G(d) level, sampling over 10 shells per atom at a density of 17 grid points per square Bohr. After RESP (Bayly et al., 1993) fitting, point charges are assigned to each atom by *Amber*'s *antechamber* tool, which also parametrizes remaining bonded and nonbonded terms according to the *gaff* forcefield (Wang et al., 2004).

MD simulations were carried out with the AMBER molecular simulation suite. For the energy minimization and early equilibration steps across the different complexes, CPU‐based calculations with *sander* were employed, while the GPU‐accelerated *pmemd* was used to run the late equilibration stages and the unrestrained MD trajectory production. Pre‐production steps consisted of the following: pre‐minimization, minimization, solvent equilibration, heating and equilibration.

Pre‐minimization is a soft initial minimization, useful for relieving the strain caused by the preparation stages and involves relaxing the modelled loops and sorting out any possible steric clashes between hydrogens belonging to the protein, ligands, and solvent. A total of 300 minimization steps were carried out on all protein and ligand hydrogens (excluding solvent ones). In detail, the protocol consists of 10 steps of the steepest descent algorithm followed by 290 steps of the conjugate gradient algorithm. On the excluded heavy atoms, a positional restraint with a harmonic force constant of 5 kcal mol^−1^ Å^−2^ was applied.

A second minimization step was also performed with the same pre‐minimization settings, but without restraints on heavy atoms and solvent. As in the previous step, a two‐stage approach was used, for a total of 300 minimization steps (10 with the steepest descent algorithm, followed by 290 with the conjugate gradient method).

In the case of the Loading + DEX complexes, whereby DEX is docked into an otherwise unliganded protein, an extra intermediate minimization step was introduced between the above described steps. This involves the minimization of hydrogens, side chains, and ligands atoms, applying restraints over the protein backbone. In addition, for the Loading + DEX complex, the final minimization step was slightly modified, restraining only DEX heavy atoms, thus allowing the active site to undergo a more optimal relaxation.

The solvent equilibration stage reduces the intrinsic artificial order introduced by *tleap* solvation. It consisted of a 9 ps simulation in the *NVT* ensemble with a short time step (1 fs) and positional restraints on every solute atom (force constant 10 kcal mol^−1^ Å^−2^). The solvent equilibration procedure was divided into three steps (3 ps each): heating from 25 K to 400 K with 0.2 ps temperature coupling; equilibration at 400 K with 0.2 ps temperature coupling; and a final cooling from 400 K to 25 K, with a relaxation of the temperature coupling from 2.0 ps to 1.0 ps.

The heating stage allows the systems to heat up from a temperature of 25 K to 300 K. This is performed throughout a 20 ps simulation in the *NVT* ensemble (using the Langevin thermostat) (Loncharich et al., 1992) with a 2 fs time step. To avoid large positional and energy fluctuations, a harmonic positional restraint was applied to all Cα atoms (force constant 5 kcal mol^−1^ Å^−2^) to limit backbone movements. The SHAKE algorithm (Miyamoto & Kollman, 1992) was applied to constrain hydrogen‐containing bonds. A new set of atomic velocities was reassigned at this stage (a different set for each replica; *vide infra*), and remaining stages inherit atomic velocities from the immediately preceding stage.

Equilibration consisted of three steps, with different atomic restraints. These simulations were carried out in the *NpT* ensemble, ensured by the combination of the Langevin thermostat (Loncharich et al., 1992) at 300 K and the Berendsen barostat (Berendsen et al., 1984) at 1 atm. The SHAKE algorithm (Miyamoto & Kollman, 1992) was used to constrain H‐bonds, and a 2 fs integration time step was used. Firstly, a 20 ps simulation was performed, relaxing the restraint on Cα atoms with respect to the heating step (3.75 kcal mol^−1^ Å^−2^). Then, an additional 20 ps with further Cα restraint relaxation to 1.75 kcal mol^−1^ Å^−2^ was carried out. Finally, a 2 ns simulation without any restraints ensured that the system is equilibrated when the production runs begin.

Each production run was 1 μs long in the *NpT* ensemble with a temperature of 300 K, kept constant by Langevin thermostat (Loncharich et al., 1992) and 1 atm pressure, controlled by Berendsen barostat (Berendsen et al., 1984). The integration step was 2 fs. Electrostatic interactions were handled using the particle mesh Ewald method (Darden et al., 1993). A cutoff of 8 Å was used for the Lennard‐Jones interactions. The SHAKE algorithm was employed to constrain all bonds involving hydrogen atoms (Miyamoto & Kollman, 1992). Finally, we performed independent 1 μs replicas per system as production runs:

- Loading‐DEX: 4 replicas
- Loading + DEX: 5 replicas
- Maturation + DEX: 4 replicas
- Maturation‐DEX: 5 replicas.
- Post‐maturation‐FKBP51 + DEX: 9 replicas
- Post‐maturation‐FKBP51‐DEX: 5 replicas
- Post‐maturation‐FKBP52 + DEX: 9 replicas
- Post‐maturation‐FKBP52‐DEX: 5 replicas

Structural and internal dynamics analyses were performed for each system on the concatenated production trajectories, obtained by combining the single replicas for each system. The stability of the trajectories was assessed by analysing the Cα RMSD of the whole complexes or of the single components of each assembly (Figure S2). We further introduce two methodological advances in MD analysis (see below), specifically designed to extract detailed information on large‐scale and allosteric motions within these multi‐protein assemblies.

### 2.2 Distance fluctuation (DF) analysis

Distance fluctuation analysis (DF) is an in‐house method used to identify protein regions (groups of residue pairs) that have a coupled motion during a MD trajectory (Castelli, Magni, et al., 2024; Castelli, Marchetti, et al., 2024; Moroni et al., 2018). This analysis takes into consideration the fluctuation of Cα atoms during the simulation and generates a pairwise matrix where a value, *DF*_*ij*_, is assigned to each amino acid pair based on its components' Cα–Cα distance:

(1) $${\mathrm{DF}}_{\mathrm{ij}}=\left\langle {\left(d_{\mathrm{ij}}-\left\langle d_{\mathrm{ij}}\right\rangle \right)}^{2}\right\rangle$$

where *d*_*i*j_ is the distance between the *i* ^*th*^ and the *j* ^*th*^ residue's Cα atoms and <> identifies the average over all trajectory frames. The *DF*_*ij*_ value measures the propensity for a residue pair to move in a coordinated manner. Specifically, the higher the value, the lower the propensity to coordinated motion is. Thus, the lower the value, the higher the coordination is during the MD trajectory. Distal regions coupled by lower *DF* values are likelier to engage in allosteric cross‐talk.

For a clearer interpretation of the differences between the DF matrices for each equilibrium and the related nonequilibrium simulation, we also introduce the ΔDF matrices. These represent the simple matrix differences between the two independent DF matrices. High absolute |Δ*DF*_*ij*_| values encode a relevant gain or loss of mechanical connection between amino acid pairs, while low absolute values represent a comparable mechanical connection.

### 2.3 RMSF

Cα root mean square fluctuations (RMSF) were computed with the *atomicfluct* command in *cpptraj Amber*'s analysis tool on the equilibrated trajectories, using the whole trajectories to build the average structure (Roe & Cheatham III, 2018).

### 2.4 HSP90 MiD—GR distances

All Cartesian distances among the points described in this work are computed with *python3* through the *mdtraj* package.

### 2.5 D‐NEMD simulations

The D‐NEMD approach (Artur et al., 2023; Balega et al., 2025; Oliveira et al., 2021; Oliveira et al., 2022; Oliveira, Edsall, et al., 2019; Oliveira, Shoemark, et al., 2019) was used to investigate how the different complexes respond to a certain external stimulus (in this case, ATP hydrolysis and DEX binding/unbinding). D‐NEMD, which integrates simulations in equilibrium and nonequilibrium conditions, enables a detailed characterization of the system's dynamical changes as it relaxes after the introduction of an external trigger.

To model ATP hydrolysis using classical physics only, we designed a perturbation protocol that reproduced the key features of the reaction according to the scheme: Glu47^−^ + ATP^4−^ + H_2_O → Glh47 + ADP^3−^ + HPO^2−^, following an approach similar to that used in our previous work investigating the nucleotide reactivity mechanism of the Hsp90 mitochondrial paralog (Serapian et al., 2021). Starting with the protonation of the Glu47 to Glh47, the reactive (nucleophilic) water was selected as the closest water molecule to the ATP ɣ‐phosphate oxygen that was not coordinated by the Mg^2+^ ion. The conversion from ATP to ADP and HPO₄^2−^ was done using the same procedure as in Castelli, Magni, et al. (2024) and Castelli, Marchetti, et al. (2024), which entailed breaking the bond between the ɣ‐ and β‐phosphates and inverting the phosphorous tetrahedral hybridization, based on the direction of the cleaved bond. The new HPO_4_^2−^ (inorganic phosphate) and Glh (protonated glutamate) were built to match the forcefield stable geometries. Glh parameters were taken from the *Amber ff14SB* forcefield, while for the inorganic phosphate, the parameters from Kashefolgheta and Vila‐Verde (Kashefolgheta & Vila Verde, 2017) were used. ATP and ADP parameters were taken from the work of Meagher and coworkers (Meagher et al., 2003).

For the DEX removal, the perturbed structures were obtained by simply removing the glucocorticoid ligand from the GR active site, without further adjustments.

**FIGURE 2:**
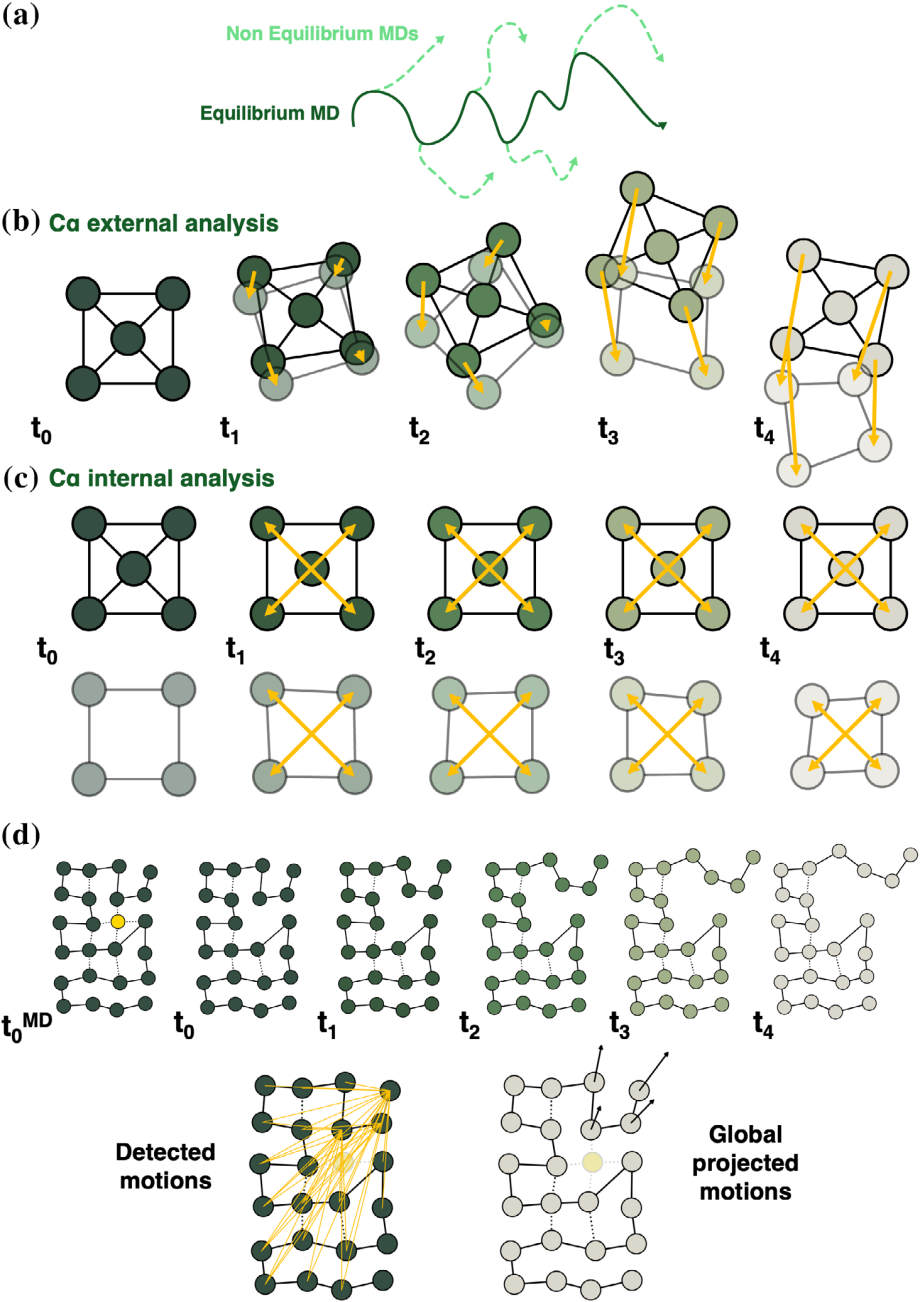
Nonequilibrium and novel analysis approaches. Schematic view of the different D‐NEMD analyses used to characterize the systems' internal dynamics. (a) Nonequilibrium trajectories initiated from the equilibrium trajectory at different times. (b) An illustrative representation of the standard method to analyze the effect of the perturbation on the system. The external analysis relies on the measure of the average deviation from the equilibrium simulation of corresponding atoms (considering also diffusive effects). (c) An illustrative representation of the newly implemented internal method to explore dynamical differences within D‐NEMD, which focuses on the differences between internal measures (such as pairwise distances). (d) Top lane: Schematic representation of the time‐dependent D‐NEMD perturbation effect, caused by ligand removal (yellow circle) in a protein‐like model (green circles). Bottom left: A representation of the residue pairs involved in the D‐NEMD analysis using pairwise distances (yellow lines), which correspond to the residues affected by the perturbation (selected by a threshold on the pairwise distances). Bottom right, vector directions and norms encode the direction and entity of the most significant residue displacements from the D‐NEMD analysis.

Starting from frames isolated from the equilibrium MD trajectories at different times (time steps for each system will be provided in the following paragraphs), instantaneous ATP hydrolysis or DEX removal was carried out, forcing the systems out of equilibrium. Only for the nucleotide hydrolysis, a brief minimization of the cleaved phosphate and the Glh with all other atoms positionally restrained was carried out to relieve the strains caused by the perturbation. Without this short relaxation, the system turned out to be unstable for several MD restarts. Then, the systems were simulated for 20 ns (Figure 2a), allowing observation of the structural changes in the complexes as they relaxed towards a new equilibrium state. The Kubo‐Onsager relation was used to extract the system's response to ATP hydrolysis or DEX removal, by comparing each pair of nonequilibrium and equilibrium trajectories at equivalent times as averages of absolute deviations between Cα positions before and after the perturbation (Ciccotti & Ferrario, 2016). This analysis allows us to obtain a description of the evolution of the system's response to the external trigger (Figure 2b). The combination of a large number of replicas performed for each perturbation (see details below), designed to accumulate robust statistics, together with the use of the subtraction technique, reduces noise and enables the identification of protein regions significantly affected by the perturbations. We outline below the various systems studied with D‐NEMD and the perturbations involved.

#### 2.5.1 Maturation + DEX complex—M‐NEMD‐ATP

The response of the Maturation + DEX complex to the hydrolysis of the two ATP molecules (M‐NEM) was taken from Castelli, Magni, et al. (2024) and Castelli, Marchetti, et al. (2024).

#### 2.5.2 Maturation + DEX complex—M‐NEMD‐DEX

To investigate the response of the Maturation + DEX complex to the removal of DEX, D‐NEMD simulations were performed by extracting frames every 20 ns over the four replicas to collect a pool of 196 starting structures as starting points for the nonequilibrium simulations. For each of these frames, the ligand DEX was instantaneously removed, and simulations were carried out for 20 ns, preserving atomic velocities. The per‐residue Cα response was averaged across all pairs of 196 simulations.

#### 2.5.3 Post‐maturation‐FKBP51 + DEX complex—F51‐NEMD‐ATP

Frames were extracted every 5 ns from the F51 + DEX simulations (over all nine replicas). These were used as starting points for the nonequilibrium simulations using ATP hydrolysis as the external perturbation. Each ATP molecule in the two active sites was hydrolysed independently. This initiated two separate non‐equilibrium simulations, mimicking hydrolysis in protomer A and protomer B, respectively. With respect to the *M‐NEMD‐DEX* simulations, the number of simulations and granularity of sampling from the equilibrium simulations are increased to reduce the noise of the analysis and enhance possible differences between the immunophilin‐containing complexes. Given the high similarity between the two systems, more sampling was in fact needed to obtain statistically significant responses. This approach was, in fact, suggested by the high similarity between the two systems in terms of sequence and structural organization.

A total of 1773 starting structures for each protomer were obtained for the non‐equilibrium simulations. For each hydrolyzed ATP structure, 20 ns simulations were carried out. The major alterations within the active sites caused numerical instability issues in a fraction of nonequilibrium simulations. Indeed, in these simulations, the sudden perturbation introduces large forces that cause energy convergence to fail. Hence, for the D‐NEMD analysis, we considered only the correctly terminated trajectories, which for this system can be summarized as follows: 1009 trajectories for ATP hydrolysis within protomer A and 1114 for protomer B. As for the M‐NEMD‐ATP analysis, the Cα deviations were computed every 1 ns for each protomer using the Kubo‐Onsager relation, as described above.

#### 2.5.4 Post‐maturation‐FKBP51 + DEX complex—F51‐NEMD‐DEX

In this system, a starting pool of conformations for D‐NEMD was obtained by collecting frames every 5 ns over the equilibrium MD trajectories, in a total of 1773 structures. For each starting structure, DEX was instantaneously removed, and the perturbed systems were simulated for 20 ns. Unlike ATP hydrolysis, the minor modifications within the GR active site did not cause system instability, and all the nonequilibrium simulations smoothly ended. The Cα deviations were computed as described above in the M‐NEMD‐DEX section.

#### 2.5.5 Post‐maturation‐FKBP52 + DEX complex—F52‐NEMD‐ATP

The alternative ATP hydrolysis of the Post‐maturation‐FKBP52 + DEX was carried out in the same way as the F51‐NEMD‐ATP. The only difference is the number of 20 ns simulations that successfully finished for each protomer: 1254 for protomer A and 1155 for protomer B.

#### 2.5.6 Post‐maturation‐FKBP52 + DEX complex—F52‐NEMD‐DEX

For the DEX perturbation‐based D‐NEMD for Post‐maturation‐FKBP52 + DEX, the analysis was carried out in the same way as for the F51‐NEMD‐DEX.

### 2.6 Analysis of internal dynamics from D‐NEMD

To further analyse the D‐NEMD trajectories, we developed a modified version of the previously mentioned D‐NEMD analysis (Figure 2b). We define the latter “external” analysis as it compares the movement of single atoms in different simulations: indeed, in the generally applied approach, an absolute Cα deviation is computed as the average distance between the equilibrium and nonequilibrium trajectory at a given amount of time from the perturbation (Figure 2b).

Here, we also calculated all intra‐complex pairwise Cɑ distances matrices for the equilibrium and nonequilibrium trajectories at equivalent points in time (Figure 2c), providing an efficient analysis of the relative (possibly coordinated) displacement of residue pairs (e.g., the motions inside the active sites, mapping which residue pairs displace the most from their equilibrium relative positions). Nevertheless, the number of data points increases as the square of the number of involved residues, making the interpretation of the results more challenging and time‐consuming. To mitigate this, we assume that, after the perturbation, averaged pairwise distances, calculated by averaging over all the collected pair‐distance matrices at a given time instant after the perturbation, should behave linearly, according to the linear response theory (LRT):

(2) $$C_{\alpha }^{i-j}\left(t\right)=a_{C_{\alpha }^{i-j}}+b_{C_{\alpha }^{i-j}}t+O\left(t^{2}\right)$$

where C_⍺_^*i‐j*^ is the distance between the C⍺s of residue *i* and residue *j* at time *t*, *a*_C⍺_^*i‐j*^ is a parameter that quantifies the displacement at *t = 0*, *b*_C⍺*i‐j*_ is the linear coefficient that relates the extent of the displacement to time increase; while *O* (*t* ^2^) represents terms of order *t* ^2^ or smaller, which are neglected in the linear approximation.

Except for the residues near the active sites (at a small number of time points), for which the displacement seems to follow an exponential law, this approach works reasonably well. Thus, we employ the parameters *a* and *b* of Equation (2) to calculate an approximate conformational response of the system at specific times.

In this way, we detect which points are internally most affected by the perturbations. To determine the direction of the perturbation, we used the combination of all the vector directions connecting an “affected” residue to the other ones. While qualitative, this approach provides an indication of the movement. Indeed, if a loop performs an opening motion, one of its residues should increase the distance to all the other residues of the protein it belongs to along a specific direction, providing the direction of the motion with respect to its surroundings. Below, we explain in more detail the procedure for extracting information from D‐NEMD simulations using the “internal” method.

With respect to the “external” analysis method, which compares the positions of individual Cα atoms in equilibrium and nonequilibrium conditions, the amplitude of the response is approximately an order of magnitude lower compared to the “internal” analysis approach.

To better understand the effect of the perturbation, the structural responses must be denoised. For this, we firstly identify the residue pairs whose absolute linearized distance difference between the equilibrium and the perturbed system is over a 0.3 Å threshold (called “visibility” threshold). Then, a frequency value for each residue is assigned by counting the number of times that, at a given time after the perturbation, the residue is involved in a pair with any other residue over the “visibility” threshold. If the frequency overcomes a certain threshold, the amino acid is considered to be “affected” by the perturbation, assessing a compromise that helps to filter out non‐significant displacements. For each “affected” residue, an associated overall displacement vector is calculated. More specifically, for the *i^th^* “affected” residue, it is given by the vectorial sum of the versors along all *i‐j* directions, each further scaled by the related C⍺^*i‐j*^ *(t)* value (see Equation 2). Thus, for each “affected” amino acid, a resulting vector is obtained and, for a better visualization, its norm is increased by a constant factor, and it is projected onto a reference structure (first frame of the first replica for each system). This way of plotting the result has the advantage of explicitly showing how the affected residues, in our case around the active site, move relative to the rest of the system, showing the net effect of the perturbation (Figure 2d).

The results obtained with the “internal” analysis approach, based on the extraction of a larger number of data linked to the calculation of all distance changes between all pairs of atoms, are more sensitive to small internal variations and movements compared to the “external” method.

### 2.7 PCA D‐NEMD

Principal component analysis (PCA) was utilized to further characterize the effect of the perturbations triggered in the systems by investigating the presence of time‐dependent collective principal movements. Hence, two time‐dependent trajectories are compared: the equilibrium one, made up of equilibrium frames after the perturbation at fixed time intervals and the nonequilibrium one, with frames extracted from the D‐NEMD trajectories at the same fixed time intervals (both trajectories are composed only of Cɑ atoms). Frames superposition on the same reference structure is followed by covariance matrix calculation (using atomic coordinates). Covariance matrix diagonalization reveals the collective movements induced by the perturbation and indicates whether these differ from those observed under equilibrium conditions. To ensure a correct comparison between the two PCA results, we use the same number of frames for both the equilibrium and nonequilibrium trajectories.

### 2.8 Tensor of gyration (TOG) analysis

One of the aims of the work is to characterize the interaction between different proteins involved in the GR maturation cycle and to assess their responses to ligand‐induced perturbations. Here, we introduce the tensor of gyration analysis (TOG), a method to characterize the macroscopical movements of different proteins and related subdomains:

(3) $$S_{\mathrm{mn}}=\frac{1}{M}\sum_{i=1}^{M}\left(r_{m}^{i}-r_{m}^{\mathrm{COM}}\right)\left(r_{n}^{i}-r_{n}^{\mathrm{COM}}\right)$$

where *S*_*mn*_ is the (*m*,*n*) element of the gyration tensor calculated on an ellipsoid‐like group of atoms; *m* and *n* are cartesian coordinate indices (*x*, *y*, and *z*); *M* is the number of atoms considered to establish the ellipsoid; *r*_*m*_^*i*^ is the *m*‐th cartesian component of the *i*‐th atom; and *r*_*m*_^*COM*^ is the center of mass of the *M* considered atoms along the *m‐th* component. All the tensors of gyration described in this work are computed with *python3* through the *mdtraj* package.

**FIGURE 3:**
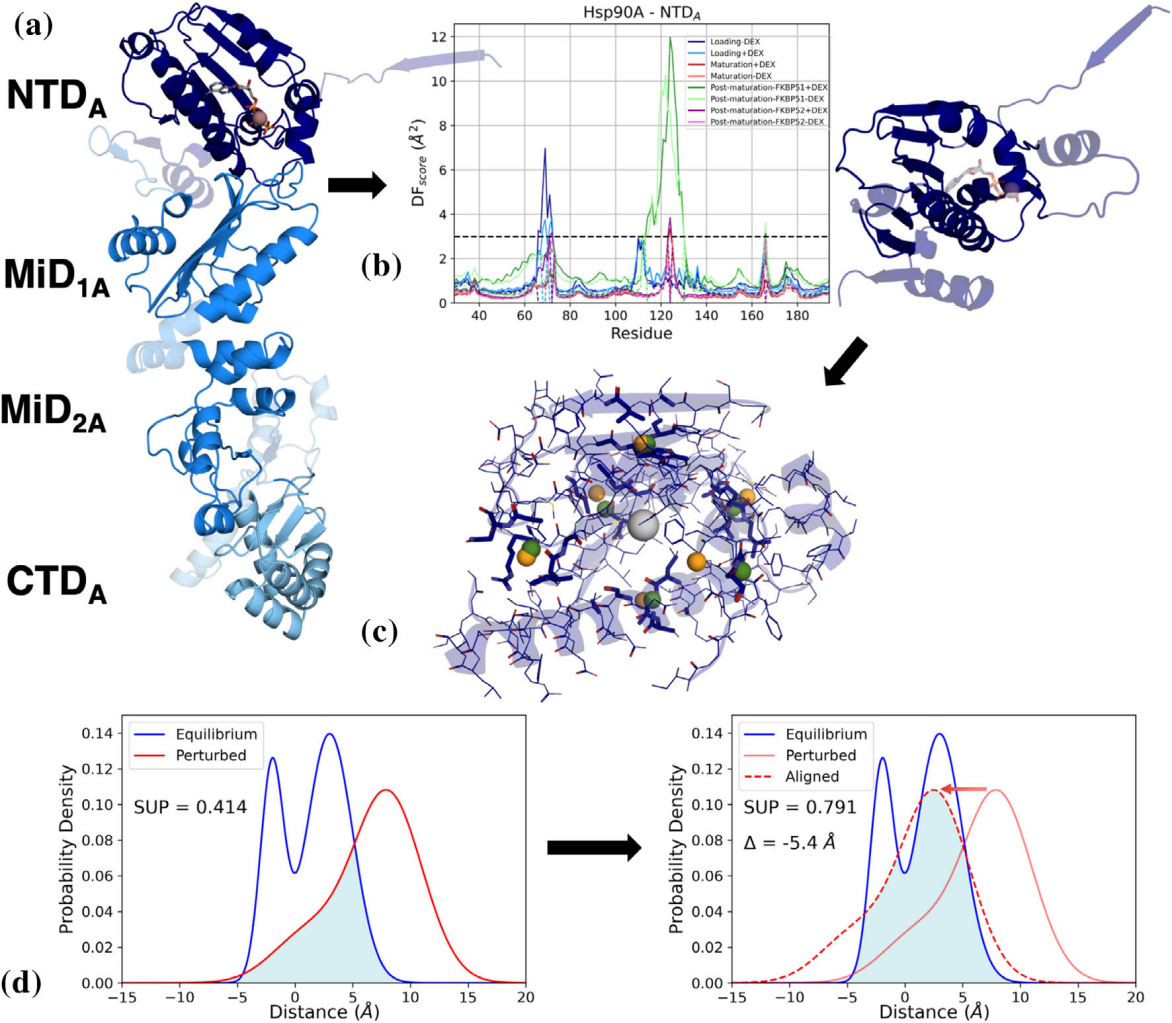
Illustrative representation of Tensor Of Gyration (TOG) analysis on the NTD_A_ of Hsp90. (a) Hsp90 protomer A is colored by its coarse domain division in NTD_A_, MiD_1A_, MiD_2A_ and CTD_A_. Residues discarded from the initial selection are represented in transparency. (b) On the left, the DF score plots of the NTD_A_ for each simulated system. The original DF score is represented by colored solid lines. The horizontal black dotted line represents the threshold to iteratively discard highly flexible amino acids. Colored dotted lines represent the DF score at the end of the iterative selection process. On the right, selected amino acids are highlighted on the NTD_A_ structure. Transparent representation highlights the common Hsp90 residues that are discarded during both the first coarse and the second refined filtering processes. (c) The results of TOG diagonalization are represented on the NTD_A_ structure (backbone as transparent cartoon). Orange dummy atoms correspond to the positions of geometrical semiaxes of the ellipsoid, while green ones represent their closest physical counterpart. The green physical spheres are extracted from the center of mass of the atoms in the surroundings of geometrical points for at least 25% of the simulation (represented as sticks). (d) Example of a superposition and alignment procedure for a pair of distance distributions (before and after the perturbation caused by introduction of the ligand) on an illustrative pair of reduced points (physical semiaxes). In the left plot, raw distance distributions from the original and perturbed dynamics (poor superposition, 41.4%). In the right plot, the perturbed distance distribution is shifted by a Δ value to the maximum degree of overlap (improved to 79.1%). The perturbed system is translated to lower distance values (negative translation value means that reduced points move away, while positive Δ identifies an approaching motion).

TOG helps describe the atomic dispersion of a given system along every direction. Firstly, each protein is segmented into ellipsoidal‐like subdomains. Indeed, the approximation of an entire domain to an ellipsoid description reduces the degree of freedom of the system, facilitating the characterization of relevant movements from a macroscopical point of view. In detail, we roughly divide each protein into one or more subdomains, based on the starting system's conformation (Figure 3a). Then, we integrate this coarse domain division with the information gathered from the DF analysis (vide supra), computing the DF^score^ from the DF matrices in the following way:

(4) $${\mathrm{DF}}_{i}^{\text{score}}=\frac{1}{M}\sum_{j=1}^{M}{\mathrm{DF}}_{\mathrm{ij}}$$

where *DF*_*ij*_ is the DF value for the amino acid pair *i* and *j*; *M* is the total number of amino acids, and the *DF*_*i*_^*score*^ represents the average DF value for the *i* ^th^ amino acid. Based on the score, it is possible to extract the residues that display an ordered and low fluctuating motion, that is, the amino acids that are mostly representative for the shape of the domain or subdomain. After this coarse and manual selection, an iterative filtering process further selects the residues to retain. At each step and for each ellipsoidal subdomain, the residue with the highest score above a specific threshold (DF^score^ >3 Å^2^) is removed, and DF scores are recalculated. This filtering process continues until all the remaining scores fall below the threshold, resulting in an optimal “compact” set of residues accounting for each biologically relevant domain/subdomain (Figure 3b). This approach is applied consistently across all the protein entities composing each assembly of the cycle. In particular, since Hsp90 and GR are present in all multi‐protein complexes, the iterative procedure selects different residues based on the considered multi‐protein model. In this case, we collect the filtered amino acid ensembles resulting from this selection process for each multi‐protein complex and unify them to obtain a coherent set of residues across the models.

Our initial division results in four groups to describe each Hsp90 monomer (NTD, MiD_1_, MiD_2_, CTD) and only a single group for GR, which consists of its core region. Regarding the other proteins, they are segmented as follows: Hsp70C into NBD and SBD domains; Hsp70S, considering only the NBD domain; Hop into three domains, namely Hop_1_, Hop_2_, Hop_3_; p23 into two domains, the core and its tail α‐helix; FKBPs are both divided into four domains: FK_1_, FK_2_, TPR_1_, and TPR_2_. Domain intervals are summarized in Table S1.

For each properly filtered subgroup, TOG calculation is carried out on every simulation frame according to Equation (3) and the resulting tensor is further diagonalized. This yields the direction of the ellipsoid axes (eigenvectors) and their length (eigenvalues) for each subdomain.

(5) $${\mathrm{TOG}}_{p}^{s}=\left\{{\mathrm{COM}}^{s};{\mathrm{COM}}^{s}\pm \sum_{i=1}^{3}\lambda_{i}^{s}{\vec{v}}_{i}^{s}\right\}$$

Thus, for each ellipsoidal subdomain *s*, seven key points *p* are computed: the center of mass (center of the ellipsoid, COM) and the six endpoints of the ellipsoids (corresponding to the semiaxes +x, −x, +y, −y, +z, −z). The endpoints are obtained as the sum of the coordinates of the center of mass and the product of the eigenvectors *v*_*i*_ and eigenvalues *λ*_*i*_ along the three dimensions, considering both the positive and negative directions.

Each COM, together with the related endpoints, becomes a dummy atom, so that we can more easily trace its position with respect to the rest of the system. However, although most endpoints can be easily mapped around a standard position, we encounter two problems. The first is point swapping, which causes dummy atoms to swap their positions on a specific axis (as due to small instabilities, the eigenvector may flip direction). The second issue relates to point rotation, for which dummy atoms rotate their positions around one axis of the ellipsoid (when two semiaxes have comparable length, tensor diagonalization can slightly change eigenvectors directions and lengths). To mitigate these issues, we anchor each geometrical point (dummy atom) to an actual (physical) atom, since these apparent movements may interfere with the interpretation of the results. To accomplish this, for each frame, a sphere is defined around each dummy atom, and atoms residing within it for at least 25% of the simulation are marked as significant. Next, we calculate the averaged pairwise distance matrix for all significantly targeted atoms, hierarchically clustering them into six groups (Figure 3c). The center of mass of each cluster (represented as a dummy atom) defines the final physical atomic anchor, thus resolving the swapping and rotation artifacts. Indeed, now, the points do not depend only on the geometrical description provided by the eigenvectors and eigenvalues, but they rely on an effective physical structural anchoring. TOG‐based decomposition provides a dimensionally reduced description of the dynamics, where high‐dimensional atomic motions are approximated by lower‐dimensional fluctuations of dummy atoms accounting for the dynamics of biologically significant domains.

To further explore the systems' dynamic behavior, PCA is performed on dummy atom trajectories to identify major collective motions, with those movies electronically provided at https://doi.org/10.5281/zenodo.18267647.

Additionally, we compare inter‐dummy atom distance distributions between systems to identify global motion trends. Overlap integral analysis quantifies how similar the distributions are between two systems for each dummy atom pair. Then, by shifting one of the two distributions in 0.5 Å increments, we find the optimal alignment (Figure 3d). Results are mapped within two different matrices: the overlap matrix, which encodes the degree of overlap between the two sets of distance distributions (before and after the translation). In the matrix, the upper diagonal values refer to the overlap before the alignment, while the lower values represent the post‐transformation overlapping. The second matrix indicates the extent of the translation necessary to reach the maximized overlap. In our case, translations are performed on the perturbed systems, so that a negative value means the perturbed system moves the atoms apart, and a positive value indicates they move closer.

In summary, TOG analysis offers a robust framework for reducing complex protein motions into interpretable geometric descriptors, enabling cross‐system comparisons and a high‐level understanding of domain‐level dynamics in multi‐protein assemblies.

### 2.9 Contact analysis and contact probability distributions

To investigate the nature of the interactions between each protein in the complexes and their remodulations after DEX‐induced perturbations, contact analysis is performed. For each frame of every MD simulation, we evaluate residue pair interactions through a pairwise distance calculation, considering only heavy atoms. We mark two residues as interacting if at least one heavy atom of each one is found closer than 4.5 Å. Application of this analysis to whole trajectories enables us to build a probability contact map between each pair of residues to identify the pairwise interactions most affected by the perturbation. We select those that show an absolute contact probability difference greater than or equal to 25%, with a minimum contact probability of 25%. A clear view is obtained by projecting these couples onto the structure and plotting a sphere at the mean point between the Cα of the two residues involved.

## 3 RESULTS

To explore the molecular determinants governing GR activation, we employed the original cryo‐EM reconstructions of the assemblies and strategically perturbed models in which the steroid ligand dexamethasone (DEX) was either removed (if present in the cryo‐EM structure) or introduced (if absent in the original cryo‐EM structure) into the GR binding pocket. These perturbations were designed to probe how substrate occupancy may influence the conformational landscape of GR and, in turn, modulate the dynamics and organization of its multi‐protein assemblies. In doing so, we seek to bridge structural observations with the dynamic interplay between ligand availability, abundance, and cellular regulation. The plausibility of ligand‐free starting states is supported by in vivo crosslinking experiments showing that GR–FKBP51/52 interactions in the absence of ligand (Baischew et al., 2023) are nearly indistinguishable from those captured in cryo‐EM structures (Noddings et al., 2023). Moreover, evidence suggests that apo complexes are indeed sampled in cryo‐EM datasets, albeit with reduced structural order compared to fully ligand‐bound states (Noddings et al., 2022; Noddings et al., 2023; Wang et al., 2022).

Given the large number of systems analyzed and the diversity of conditions under which they are studied, we will first present pairwise comparisons of the different models, and then discuss general mechanistic implications. In this framework, we start by analyzing the dynamic differences between a certain complex in its originally resolved cryo‐EM conditions and in its perturbed version (with or without bound DEX). Specifically, in the case of the Loading complex, this entails studying the original complex in which the steroid is not bound to the GR active site, and the complex in which DEX has been artificially docked to GR. Similarly, DEX is artificially removed from the active site of GR in the Maturation + DEX, Post‐maturation‐FKBP51 + DEX, and Post‐maturation‐FKBP52 + DEX complexes, and their dynamics are compared with those of the cognate complexes, whose original CryoEM structures were resolved with DEX bound. Finally, we compare the Post‐maturation‐FKBP51 + DEX and Post‐maturation‐FKBP52 + DEX complexes to investigate the basis for the intriguing differences in function between two proteins that are, on first inspection, highly similar.

The presence or absence of DEX encodes a perturbation aimed at unveiling the relationships between the steroid‐binding region in GR and the regulation of the internal dynamics of the GR‐processing complexes, which underlie the progress along the different steps of the chaperoning cycle. We anticipate that the ligand, despite being smaller than the full assembly, significantly affects the dynamical behavior of the complexes. From the comparative analyses of multiple trajectories, we extract insights into the long‐range communication mechanisms and the dynamic cross‐talk among the different constituents of the assemblies, which ultimately determine their functional roles.

For each pair of structures, we compare the pairwise amino acid (Distance Fluctuation) DF matrices, which highlight the portions of the complex that move in a mechanically coordinated manner (see Section 2). In general, low pairwise DF values between (groups of) distal residues, which may even be located in different proteins, are indicative of allosteric coordination. DF analysis is intended to identify the common and differential networks of internal dynamic interactions that can be reconnected to the functionally oriented motions of the various complexes.

DF analysis is complemented by RMSF analysis (see Section 2). Next, to analyze collective and macroscopic motions, we introduce the novel TOG analysis (see Section 2), together with elucidation of differences in probability contact maps.

Next, we integrate data from equilibrium simulations with the results of different out‐of‐equilibrium D‐NEMD simulations. D‐NEMD tracks the diffusion of the structural responses introduced by a specific perturbation (in this case, introducing or removing the ligand from GR, or hydrolyzing ATP within the chaperones), providing additional information on the allosteric response to different stimuli (see Section 2). In detail, where possible, for each system, we employ two different perturbations: one forcing alternating nucleotide hydrolysis in each respective Hsp90 binding site (ATP in NTD_A_ and NTD_B_), and the other removing the DEX ligand from the GR binding pocket.

To retain consistency, presentation of the results will follow the same scheme for all the investigated systems: effects on GR, effects on the GR‐Hsp90 dynamic network, effects on Hsp90, and effects on the cochaperones network.

### 3.1 Loading‐DEX versus loading + DEX

In the cryo‐EM structure of the Loading‐DEX complex (L‐DEX; Figure 1b), GR is not bound to any steroid ligand. To generate a DEX‐bound model (Loading + DEX; L + DEX), we use an induced‐fit docking (IFD) strategy (see Section 2). Here, the goal of artificially inserting DEX into GR is to investigate whether interaction with the substrate (whose local concentrations can be high under stress conditions) could be a factor in pushing the cycle towards the subsequent stages. Interestingly, while the IFD protocol was designed to accommodate DEX in the otherwise empty and ligand‐incompetent structure of the Loading complex, it is interesting to observe that ligand‐competent GR conformations (potentially prone to recognize the steroid and with an RMSD of the GR core backbone ~1.7 Å with respect to the steroid bound ones) indeed exist on the energy landscape of partially unfolded GR in complex with Hsp90. This is a qualitative observation which, however, supports the viability of our hypothesis according to which a chemical perturbation may help reshape the equilibrium dynamics of the chaperone machinery.

**FIGURE 4:**
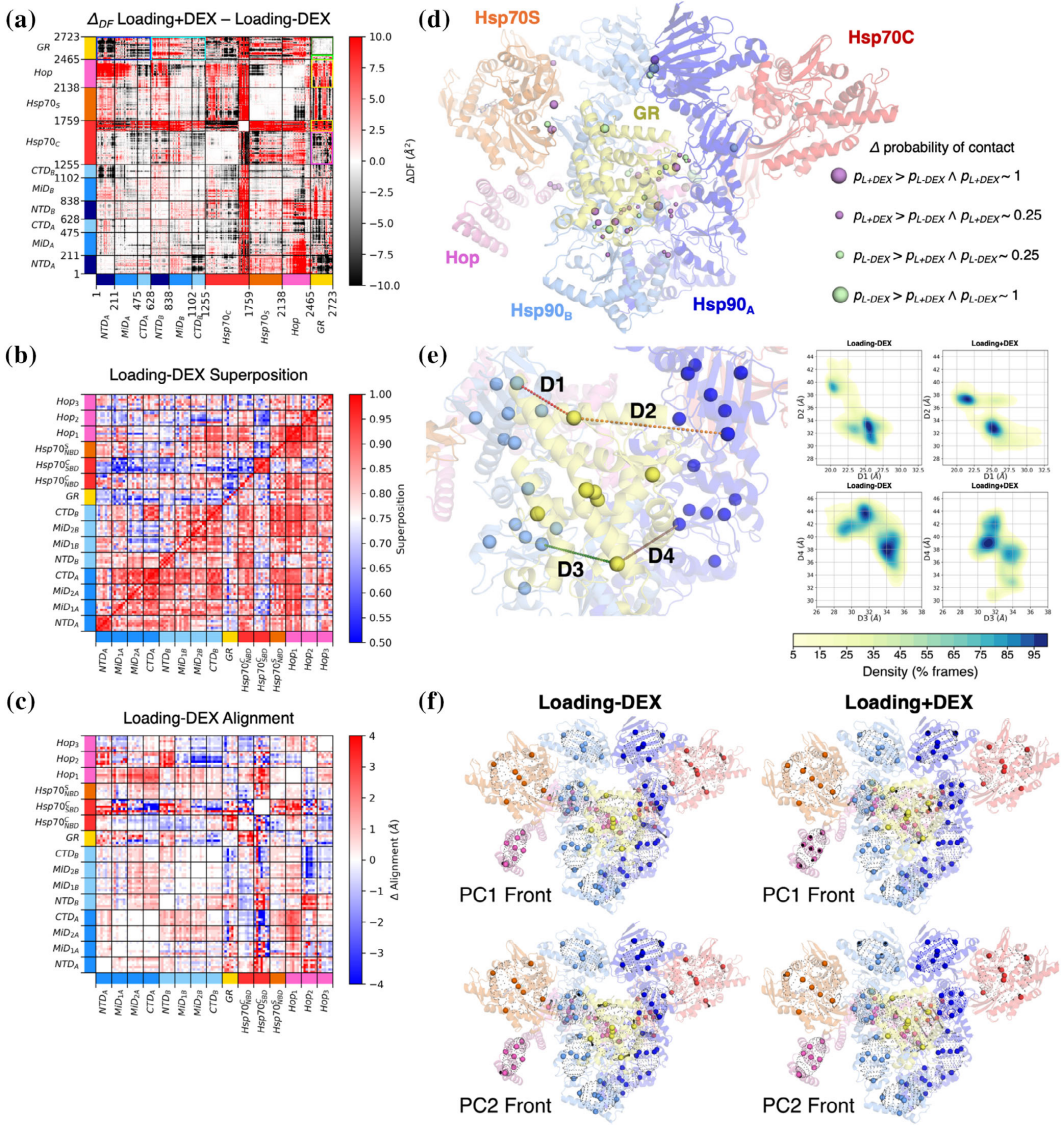
Dynamic analysis of the Loading complex in different ligand states. (a) ΔDF matrix for the Loading complex: The matrix is obtained by subtracting the equilibrium matrix (Loading‐DEX) from the perturbed one (Loading + DEX). Black regions indicate that the DF value is higher in the equilibrium matrix (coordination gain after DEX insertion), while red areas mean the opposite (coordination loss after DEX insertion). (b) The superposition map contains the overlap integral values for each pairwise distance distribution. The upper diagonal depicts values before superposition, while the lower diagonal represents the values after superposition. Red values represent an overlap of 80% or higher, indicating a similar dynamical behavior between the unperturbed Loading and perturbed Loading + DEX MDs. White/blue values underlie a non‐optimal superposition due to the different shapes of the distributions and, thus, a marked dynamical difference. (c) The alignment map represents the Δ value pertaining to the translation with the highest degree of overlap for each reduced point pairwise distance distribution between Loading‐DEX and (perturbed) Loading + DEX trajectories. Blue spots (Δ < 0 Å) mean that the perturbation has increased the distance between reduced points, while red spots (Δ > 0 Å) mean the opposite. (d) Projection of differences in probability of contact onto the Loading complex structure. Each sphere indicates the presence of a statistically significant modified interaction between each inter‐protein residue pair contained in the unperturbed and perturbed contact matrices. Spheres mark the midpoint between the two Cα atoms involved in each interaction, identifying the approximate contact location. Contacts more likely in the Loading‐DEX complex are shown in green; those more likely in Loading + DEX are in violet. Sphere radius reflects the maximum likelihood (probability) of the corresponding contact. (e) On the right, 2D histograms of two key distance pairs of TOG reduced points (D1‐ D2 and D3‐D4); On the left, their representation on the structure of the Loading complex. The TOG reduced points reflect the color assigned to each domain (dark blue for protomer A, light blue for protomer B, yellow for GR). For the D1‐D2 pair, the analysis of Loading‐DEX and Loading + DEX distributions show that DEX (dexamethasone) insertion causes GR and MiD_B_ to drift apart, but brings GR and MiD_A_ closer. D3‐D4 plots, instead, show a similar, but more nuanced behavior, with a reduced exploration of D3 distance. Overall, the GR is prone to rotate around its terminal tail loop approaching protomer A with its upper part and protomer B with the lower part. (f) Projection of the first two PCA eigenvectors (vectors as arrows) onto structures of the Loading complex, from unperturbed (Loading‐DEX; left) and perturbed simulations (Loading + DEX; right). Spheres represent the TOG‐derived reduced points, while dots outline the shape of the identified ellipsoids that include each cluster of spheres.

Interestingly, DEX binding causes modifications in the overall dynamical behavior of the entire complex. While in general the DF matrices for L‐DEX and L + DEX show a similar alternation of blocks of high and low coordination, which reflect the domain organization of the partners, a closer inspection and the analysis of the difference matrix ΔDF underscores interesting differences in the internal coordination of the two systems, suggesting that DEX insertion remodels the dynamic network of the complex (Figure 4a).

Indeed, upon sudden insertion of DEX, the core of GR displays a generalized gain of coordination (Figure 4a; dark green box), while the unfolded tail enhances its fluctuations with respect to the rest of the protein (Figure 4a; green box). DEX‐induced rigidification of the receptor motion is also confirmed by RMSF analysis (Figure S3A,B).

Importantly, DEX insertion causes visible modifications in the TOG superposition map and alignment (Figure 4b,c), where, even after the alignment procedure, intra‐protein points display distinct behaviors within GR.

With regards to interactions with Hsp90, ΔDF indicates increased coordination with protomer A upon DEX insertion (Figure 4a; blue box). The opposite occurs with protomer B, where GR loses coordination with most regions except CTD_B_ (Figure 4a; cyan box).

The variation in the internal dynamics between L‐DEX and L + DEX is also reflected in the differences in contact maps projected onto the structure (Figures 4d and S4). DEX causes modifications on the contact surfaces between GR and Hsp90. In particular, GR partially loses an interaction with the upper part of Hsp90 MiD_B_ domain (between Q404 of Hsp90 and K703 of GR), while the majority of reshaped interactions involve the interface with protomer A (in the region 319–355 of Hsp90_A_ with 580–590 of GR) and with protomer B (between CTD_B_ and both the GR tail and loop around residue 625). Interestingly, the modulation of the interaction spectrum reflects the variations in coordination patterns revealed by the ΔDF matrix.

DEX insertion causes a clamp‐like closure of the Hsp90 protomers, as evidenced by a generalized shortening of pairwise distances in TOG points, especially in the region between MiD_2A_‐CTD_A_ and MiD_2B_‐CTD_B_ (Figure 4c). The beginning of the domain closure process is also visible in the probability contact map (Figures 4d and S4). Consistent with these observations, TOG analysis confirms that the behavior of the system's subdomains is remodeled (Figure 4b). Indeed, the superposition matrix shows a generally low value for almost all GR‐Hsp90 interactions, indicating that the steroid significantly modifies client dynamics.

To better analyze the differences in behavior between the L‐DEX and L + DEX systems, we compute the distance distributions between the center of mass of the Hsp90's MiDs and the center of mass of the GR (only the compact globular portion) (Figure S5A–C). L‐DEX simulations always show a narrower distribution compared to the L + DEX ones. This tendency remains consistent even when considering the two protomers separately (center of mass of MiD_A_ and MiD_B_). The absence of DEX thus appears to restrain the fluctuation of the GR with respect to Hsp90 MiDs.

Interestingly, DEX stimulates GR to move closer to Hsp90, especially to Hsp90 MiD_A_. Additionally, two kernel density estimate (KDE) plots (2D histograms) on two pairs of different distances (calculated using TOG points in MiD_A_, GR, and MiD_B_, see Figure 4e) highlight that the insertion of DEX determines the onset of a rotational motion, which significantly enables GR to explore a different conformational space around MiD domains (Figure 4e). RMSF analysis of Hsp90 is, in general, similar to the one described above (Figure S6A,B).

Modification to the GR coordination network also affects the other components of the assembly. Specifically, the mechanical connection between Hsp70C_SBD_ and Hop with GR is enhanced in the L‐DEX simulation (Figure 4a; yellow boxes), while it decreases between Hsp70C_NBD_ and GR (Figure 4a; pink box). Such differences are also present in the TOG (Figure 4b,c) and contact analysis (Figure 4d). In particular, Hsp70C_SBD_ exhibits a completely different dynamical behavior compared to all other compact domains in the system, with a poor overall superposition in the distance distribution matrix. Hsp70C_NBD_ tends to move slightly away from Hsp90 NTDs, exhibiting a mixed pattern of increase/decrease in coordination. Consistent with this, Hop's mechanical coordination is modified upon ligand insertion. Its degree of connection is higher in L‐DEX simulations with Hsp90 (NTD_A_, MiD_A_, and NTD_B_) and Hsp70C (especially with the NBD domain). The impact of DEX insertion on Hop is particularly noticeable in the Hop_1_ domain (Table S1): this domain mutates its contact network, losing contacts in the GR tail region, but increasing interactions with the MiD_B_ domain. In fact, a general closure towards the lumen is observed for Hop_1_, together with a tendency to form contacts with MiD_B_, via the helix connecting Hop_2_ and Hop_3_.

Finally, the modification of collective motions caused by DEX insertion is analyzed using PCA on TOG trajectories (Figures 4f, S7, and S8A), with results aligning well with previous ones. Movies for the first and second principal components (PC1 and PC2) are provided at the link: https://doi.org/10.5281/zenodo.18267647. In the case of L‐DEX, the PC1 is associated with wide movements of both Hsp70C domains, together with a displacement of Hop_2_. In the L + DEX complex, the movement of Hsp70C_NBD_ disappears, replaced by the introduction of a rotational movement of GR around its disordered tail axis. In PC2, this rotational movement is also observed in the L‐DEX complex, together with some sparse motions on Hsp90_A_ NTD and Hsp70C_NBD_. Again, Hop_2_ domain displays wide motions. In the L + DEX complex, instead, larger movements involve only Hsp70C_SBD_.

Taken together, these data reveal interesting biological insights. DEX binding to the active site of GR causes long‐range interaction network remodeling with Hsp90. Coordination variations also include the interplay between GR and both Hsp70C_SBD_ and Hop, which are the protein components in direct contact with the GR unfolded tail. In particular, Hsp70C_SBD_ shows strong coordination with all members of the assembly in the absence of steroid inside GR, suggesting a pivotal role for this specific Hsp70 domain in organizing the platform for GR loading and processing. The same occurs with Hop, which displays generally better coordination with almost the whole complex when DEX is not bound (i.e., in L‐DEX). The dynamics of these cochaperones in the L‐DEX simulation prompt them to respond to perturbations in client conditions, sensing changes in both the internal dynamics and the large‐scale motions of GR. Indeed, the widespread loss of coordination upon steroid binding may trigger a signal that disrupts interactions and coordination with Hsp70C_SBD_ and Hop, favoring their detachment and ultimately setting the stage for progression to the Maturation + DEX complex.

### 3.2 Maturation + DEX versus maturation‐DEX

**FIGURE 5:**
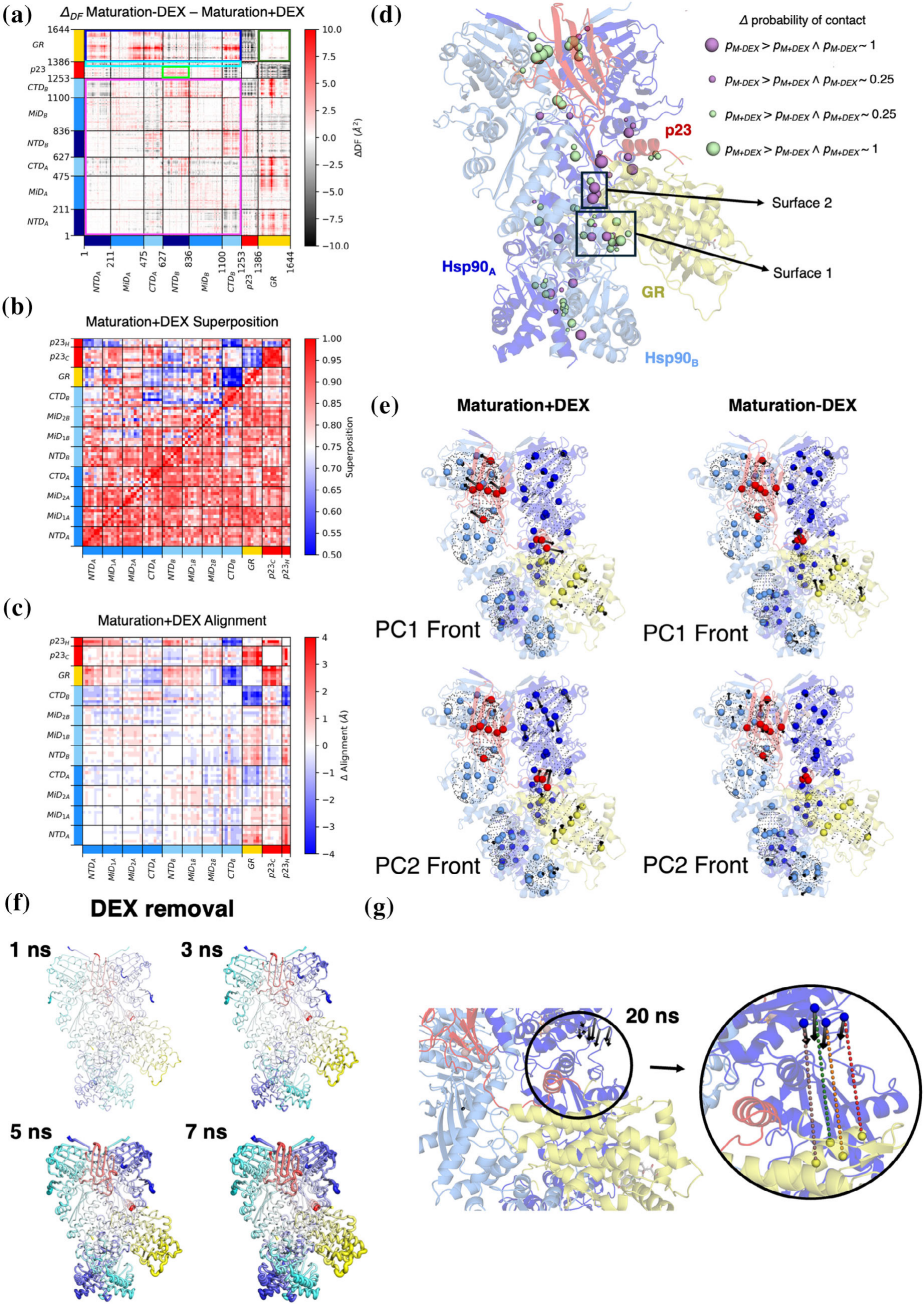
Dynamic analysis of the Maturation complex in different ligand states. (a) ΔDF matrix for the Maturation complex: Subtraction of the Maturation + DEX DF matrix (unperturbed) from the Maturation‐DEX (perturbed by instantaneous dexamethasone removal). (b) The superposition map contains the overlap integral values for each pairwise distance distribution. The upper diagonal depicts values before superposition, while the lower diagonal represents the values after superposition. Red values represent an overlap of 80% or higher, indicating a similar dynamical behavior between the unperturbed Maturation + DEX and perturbed Maturation‐DEX MDs. White/blue values underlie a non‐optimal superposition, due to the different shapes of the distributions and, thus, a marked dynamical difference. (c) The alignment map represents the Δ value pertaining to the translation with the highest degree of overlap for each reduced point pairwise distance distribution between Maturation + DEX and (perturbed) Maturation‐DEX trajectories. Blue spots (Δ < 0 Å) mean that the perturbation has increased the distance between reduced points, while red spots (Δ > 0 Å) mean the opposite. (d) Projection of differences in probability of contact onto the Maturation complex structure. Each sphere indicates the presence of a statistically significant modified interaction between each inter‐protein residue pair contained in the unperturbed and perturbed contact matrices. Spheres mark the midpoint between the two Cα atoms involved in each interaction, identifying the approximate contact location. Contacts more likely in the Maturation + DEX complex are shown in green; those more likely in Maturation‐DEX are in violet. Sphere radius reflects the maximum likelihood (probability) of the corresponding contact. Black rectangles depict the points corresponding to the surfaces discussed in the main text. (e) Projection of the first two PCA eigenvectors (vectors as arrows) onto structures of the Maturation complex, from unperturbed (Maturation + DEX; left) and perturbed simulations (Maturation‐DEX; right). Spheres represent the TOG‐derived reduced points, while dots outline the shape of the identified ellipsoids that include each cluster of spheres. (f) External D‐NEMD simulation results for DEX (dexamethasone) removal from the Maturation + DEX complex after 1, 3, 5, 7 ns from the perturbation instant. The radius assigned to the putty representation is proportional to the average degree of perturbation felt by each region once the ligand is removed. (g) Internal D‐NEMD simulation results for the Maturation + DEX complex upon dexamethasone removal after 20 ns from the perturbation. Arrows indicate the average overall direction and magnitude of the captured perturbed motion. In the circle, the magnification of the area interested by the perturbation with arrows indicating the four most relevant amino acids displacements. For a clarified view, dotted lines connect each affected residue with the Cα of the first amino acid ideally intercepted along the direction of motion.

In contrast to the Loading‐DEX complex, DEX is already bound to GR in the cryo‐EM structure of the Maturation + DEX complex. Here, we run simulations in which the ligand is artificially removed from the almost folded, active receptor. As in the previous case, the individual DF matrices from the M + DEX and M‐DEX simulations share an overall block character consistent with the domain organization of the constitutive partners. Differences emerge from the analysis of the ΔDF matrices (Figure 5a). Sudden DEX removal causes a minor loss of GR internal coordination, as shown by the mild increase of pair fluctuations in the client (Figure 5a; dark green box). Such changes are also present in the RMSF plots (Figure S3). Importantly, DEX removal does not cause any important modification in the 3D organization of GR, as visible from TOG analysis (Figure 5b,c), where a good overlap is present among intra‐GR points in the superposition matrix.

In terms of interactions with Hsp90, ΔDF shows that GR loses coordination with almost the entire Hsp90 dimer upon DEX removal, except for a slight increase in the connection between GR and both NTD_B_ and MiD_B_ (Figure 5a; blue box). This is accompanied by a reshaping of the contact probability at the GR/Hsp90 interfaces (Figure 5d). In detail, in M‐DEX there is a generalized loss of contact probability between the GR terminal tail and the luminal amino acids, with an interesting remodeling of the two surfaces in contact between GR and Hsp90_B_. The first surface is mainly located around the partially folded ɑ‐helix of the GR tail (residues 533–539 of GR) and two portions of Hsp90_B_ client binding site, positioned around CTD_B_ and MiD_B_ domains (residues 530–534 and 605–637 of Hsp90_B_). Consistent with these observations, the TOG alignment map for the GR/Hsp90 pair (Figure 5c) indicates that GR globally tends to detach from CTD_B_, increasing all the pairwise distances between GR and CTD_B_ points upon DEX removal.

The second surface remodeling involves a more localized area, encompassing residues 453–454 of Hsp90_B_ and 699–703 of GR (Figure 5d). Upon DEX removal, a constant increase in contact probability between GR and the Hsp90 MiD_1B_ domain is observed. In parallel, TOG alignment supports global modifications with GR moving towards Hsp90 MiD_1B_‐NTD_B_ domains, together with an increase in coordination with the chaperone. Overall, DEX removal induces a movement of GR towards the chaperone NTDs (Figure 5c). DEX removal also causes a tightening of the distance distribution and a shift towards lower values, reflecting a rigidification of the interaction and a tighter approach to MiD_B_ (Figure S5C).

The effects of DEX removal extend to Hsp90 inter‐protomer interactions. The ΔDF matrix shows widespread alterations in the coordination patterns between protomers A and B (Figure 5a; pink box), with several residue pairs changing their contact probability inside all domains (Figure 5d). Despite this internal rigidification, TOG analysis captures only minor distance changes among all Hsp90 subdomains, indicating that globally the closed state of Hsp90 in this complex is mainly unperturbed. Hsp90 is thus in its activated state in the Maturation + DEX complex.

Interestingly, DEX removal also affects the p23 coordination network. The absence of DEX causes a loss in the coordination between p23 and the directly bound NTD_B_ (Figure 5a; green box). Contact probability analysis is consistent with this, as the interactions between p23 and NTDs decrease in frequency (Figure 5d). Additionally, the TOG analysis further reinforces this observation since the p23 core and the NTD_B_ domain undergo a detaching motion (Figure 5c). In contrast, the mechanical connection between p23 and GR is strengthened when DEX is not bound. Indeed, the TOG analysis unveils a tightening of the GR‐p23 cores, along with an increase in contact probability between GR and the p23 ⍺‐helix tail. It is worth noting that both the GR loop, residing in the Hsp90's lumen, and the p23 ⍺‐helix tail decrease their coordination with the whole complex in the M + DEX simulation with respect to the M‐DEX (Figure 5a; cyan box).

The general trend shows that the DEX‐bound Maturation + DEX complex is more rigid than the unbound one, while significant reorganization of interactions among GR, Hsp90, and p23 emerges as the ligand is removed, with GR‐p23 contacts increasing.

Consistent with these data, PCA of the reduced TOG system shows that DEX removal causes several changes in the principal components of motion (Figures 5e, S8B, and S9). We provide PC1 and PC2 movies at the link: https://doi.org/10.5281/zenodo.18267647.

The main effect on PC1 reflects the results of previous analyses. Indeed, the absence of DEX mainly affects GR, causing a tilting motion that is propagated to p23 ⍺‐helix tail and, to a minor extent, to both the p23 core and Hsp90/NTD_A_. Nevertheless, such movement is completely opposite to that when DEX is bound, where GR tends to move towards CTDs, accompanied by a p23 detaching motion. PC2 highlights a difference between the native cryo‐EM model and perturbed systems: the M‐DEX PC2 is characterized by a collection of dispersed movements on Hsp90, whereas the PC2 of M + DEX dynamics is similar to PC1 of the M‐DEX simulation. Thus, we can conclude that DEX removal promotes tilting of GR towards NTDs, disfavoring the movement towards CTDs, an effect that is significant when DEX is bound.

Overall, DEX appears to facilitate stabilization of the complex between the folded GR and the chaperone machinery, represented by Hsp90 and p23.

Nonequilibrium simulations using the D‐NEMD approach were also performed to gain additional insight into the dynamical changes occurring within the complexes. In this case, the external trigger applied in the D‐NEMD simulations consists of ATP hydrolysis taking place in only one of the Hsp90's NTD active sites at a time within the Maturation + DEX complex. As shown previously (Castelli, Magni, et al., 2024; Castelli, Marchetti, et al., 2024), for both the NTD_A_ and NTD_B_, the signal caused by the instantaneous perturbation accumulates in the key regions of the maturation allosteric pathway. Signal communication includes cross‐talk between the two NTDs, where the active sites reside, and strong communication with the p23 cochaperone, both CTDs and, notably, the active site‐containing region of GR. Importantly, these results are entirely consistent with the signal transduction caused by the DEX instant removal (Figure 5f). Indeed, when the perturbation is caused by the instantaneous removal of the steroid, the most affected regions are again the entire p23, the CTDs, and both NTDs, which comprise the active sites.

The “internal” analysis method is applied to D‐NEMD to investigate the dynamical responses of systems to instantaneous DEX removal. This analysis highlights one small, interesting motion involving one loop of Hsp90_A_ (residues 222–226, the remodeled charged linker) approaching GR (Figures 5g and S10A–C). Four distances, aligned with the main direction of motion, are linearly fitted to show the extent of the motion (Figures 5g and S10B).

Overall, the D‐NEMD responses show a delicate yet extensive dynamic interplay between GR, its ligand state, and the Hsp90‐p23 complex. The substrate in the folded and binding‐competent client protein helps stabilize an extensive network of contacts and affects the dynamic behavior of the different domains, with an impact that pervades the whole complex, well beyond the GR binding site. Key functional regions of Hsp90 (catalytic NTDs, client binding region) and of p23 (the long helix expected to sense the state of the client) are indeed affected by the DEX removal from the client.

Our findings suggest that the interplay between assembly dynamics and ligand binding in the GR carries significant biological implications. Complex stability emerges as a key determinant of steroid processing capacity: a more stable Maturation + DEX complex could favor the production of functional GR, enabling enhanced accommodation and metabolism of steroid substrates. At elevated steroid concentrations, stability and functional competence become essential to sustain efficient processing. Within this framework, we propose a model in which dynamic coupling between the GR active site and its chaperone partners provides a platform for conformational remodeling and stabilization of the client protein. Moreover, the intrinsic plasticity of the Maturation + DEX complex likely facilitates its assembly and disassembly, ensuring adaptability throughout the successive stages of the folding cycle.

### 3.3 Post‐maturation‐FKBP51 + DEX versus post‐maturation‐FKBP51‐DEX

**FIGURE 6:**
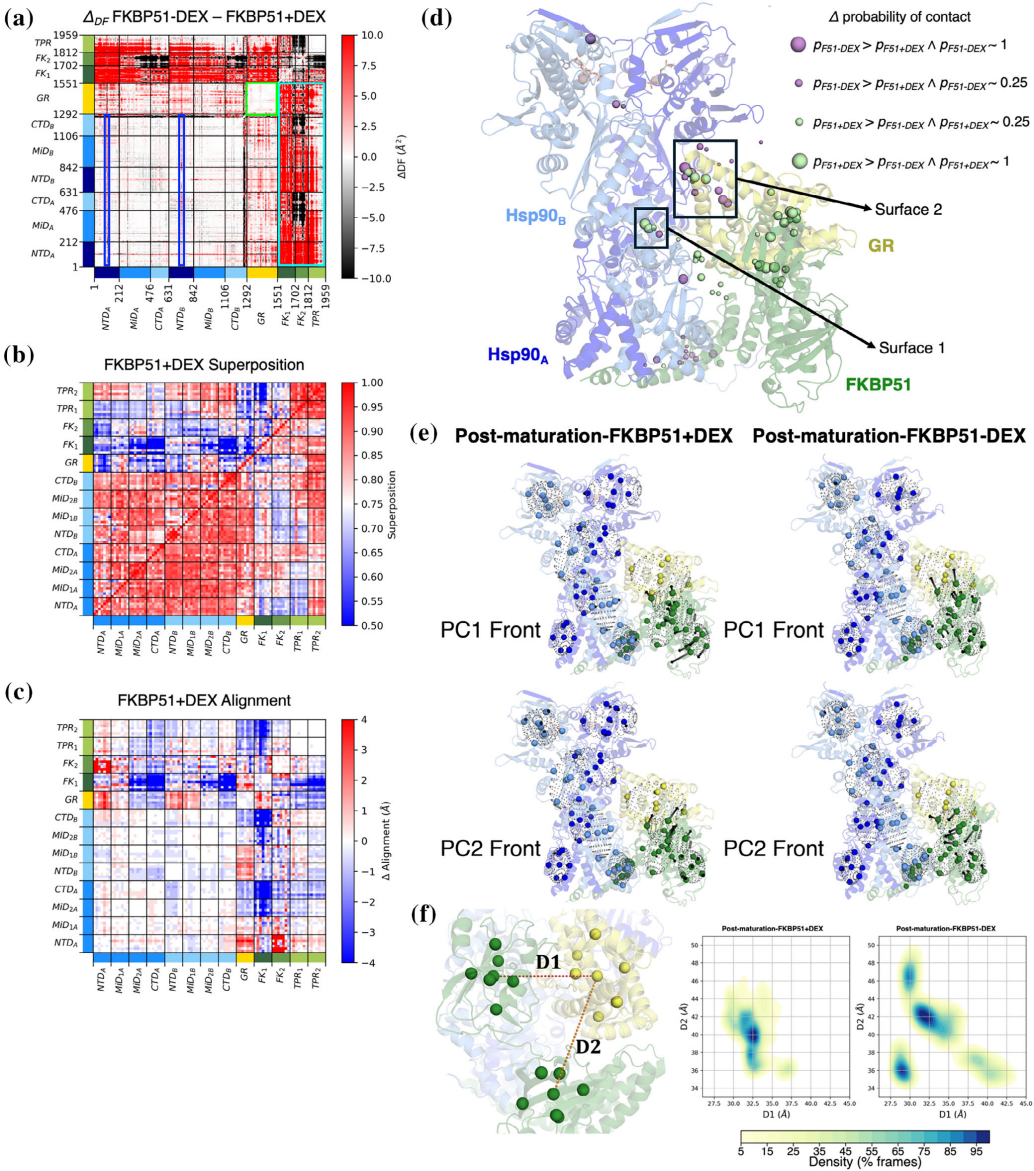
Dynamic analysis of the Post‐maturation‐FKBP51 complex in different ligand states. (a) ΔDF matrix for the Post‐maturation‐FKBP51 complex: Subtraction of the Post‐maturation‐FKBP51 + DEX DF matrix (unperturbed) from the Post‐maturation‐FKBP51‐DEX (perturbed by instantaneous dexamethasone removal). (b) The superposition map contains the overlap integral values for each pairwise distance distribution. The upper diagonal depicts values before superposition, while the lower diagonal represents the values after superposition. Red values represent an overlap of 80% or higher, indicating a similar dynamical behavior between the unperturbed Post‐maturation‐FKBP51 + DEX and perturbed Post‐maturation‐FKBP51‐DEX MDs. White/blue values underlie a non‐optimal superposition, due to the different shapes of the distributions and, thus, a marked dynamical difference. (c) The alignment map represents the Δ value pertaining to the translation with the highest degree of overlap for each reduced point pairwise distance distribution between Post‐maturation‐FKBP51 + DEX and (perturbed) Poat‐maturation‐FKBP51‐DEX trajectories. Blue spots (Δ < 0 Å) mean that the perturbation has increased the distance between reduced points, while red spots (Δ > 0 Å) mean the opposite. (d) Projection of differences in probability of contact onto the Post‐maturation‐FKBP51 complex structure. Each sphere indicates the presence of a statistically significant modified interaction between each inter‐protein residue pair contained in the unperturbed and perturbed contact matrices. Spheres mark the midpoint between the two Cα atoms involved in each interaction, identifying the approximate contact location. Contacts more likely in the Post‐maturation‐FKBP51 + DEX complex are shown in green; those more likely in Post‐maturation‐FKBP51‐DEX are in violet. Sphere radius reflects the maximum likelihood (probability) of the corresponding contact. Black rectangles depict the points corresponding to the surfaces discussed in the main text. (e) Projection of the first two PCA eigenvectors (vectors as arrows) onto structures of the Post‐maturation‐FKBP51 complex, from unperturbed (Post‐maturation‐FKBP51 + DEX; left) and perturbed simulations (Post‐maturation‐FKBP51‐DEX; right). Spheres represent the TOG‐derived reduced points, while dots outline the shape of the identified ellipsoids that include each cluster of spheres. (f) On the right, the KDE plot of distances between the center of mass of GR core and centers of mass of FK_1_ (D1) and FK_2_ (D2) domains respectively, in the Post‐maturation‐FKBP51 equilibrium simulation with (and without) DEX ligand. When the DEX is bound, the distances weakly fluctuate in a single state, in a rigid situation. Annihilation of the ligand reshapes the interactions. Indeed, three additional separated states appear, describing two different motions. The former is a contextual approach between the GR and the two FK domains, the latter is a bouncing movement in which GR moves between the two domains. On the left, the representation of the investigated distances onto the structure of the Post‐maturation‐FKBP51 complex.

Similar to the Maturation + DEX complex, the WT Post‐maturation‐FKBP51 + DEX has DEX bound to the GR binding site. As for the above‐described systems, the ΔDF difference matrix between the DEX‐bound and unbound models indicates that the internal residue‐pair fluctuation network of GR is remodeled by the perturbation, with DEX presence coherently favoring GR internal coordination (Figure 6a; green box). A rigidification/stabilization of GR is also visible in the RMSF analysis (Figure S3A,B), and the TOG data indicate minor internal shifts in GR in the absence/presence of the ligand (Figure 6b,c).

DEX removal causes a generalized loss of coordination between GR and Hsp90, as corroborated by TOG and contact analysis. Indeed, DEX removal reshapes two contact surfaces between Hsp90 and GR (Figure 6d). The first surface is located within the Hsp90 lumen at the interface with the GR tail loop, where most of the interactions decrease in contact probability. The second surface lies between the GR core and Hsp90 MiD_1A_ domain, where the DEX removal induces an overall increase in the number of contacts and their frequency. This increased contact probability reflects the rigid‐body motion of the globular region of GR towards the upper domains of Hsp90 (NTDs and MiD_1_s), contextually detaching from CTDs and MiD_2_s (Figure 6b,c). Ligand removal exerts an asymmetric effect on Hsp90, with a mixed, non‐homogeneous pattern of increases and decreases in coordination, particularly evident at the two active sites of Hsp90 (Figure 6a; blue boxes). The TOG and the contact analysis follow the ΔDF trend, with diffuse changes in contact interactions.

Strikingly, DEX removal has a major impact on FKBP51. A loss of mechanical coordination in the F51‐DEX simulations with all the assembly members is, in fact, noticeable compared to the F51 + DEX simulations (Figure 6a; cyan box). The loss of coordination for FKBP51 is accompanied by a reduced probability of contacts between GR and FKBP51 (Figure 6d). In the perturbed system, the dynamics of FKBP51 are significantly different from the unperturbed ones (low values in the superposition matrix, especially in FK1‐FK2 domains, Figure 6b). Indeed, all domains tend to dissociate from the Hsp90‐GR complex, showing enhanced fluctuations. The change in the contact network also involves the Hsp90‐FKBP51 interaction, with the immunophilin detachment from the CTD_B_ (Figure 6d). Nevertheless, an increase in contact probability is observed between the CTD_B_ C‐terminal loop and the TPR_2_ domain of immunophilin. Additionally, although the previously mentioned CTD_B_ interaction remains stable, the TPR_2_ domain shows the most significant superposition trend, identifying a global detachment motion from the other complex components (Figure 6b).

PCA on the reduced TOG trajectories (Figures 6e, S8C, and S11) highlights differences in principal component patterns between the original cryo‐EM and the perturbed models. PC1 and PC2 movies are provided at the link: https://doi.org/10.5281/zenodo.18267647. This analysis confirms that DEX removal impacts global dynamics. Differences involve all FKBP51 domains, and the motions of immunophilin emerge as the dominant component of dynamics. In general, motions identified by both PC1 and PC2 in F51‐DEX indicate a significant remodeling of interaction networks, which ultimately weaken the connection between the immunophilin and GR. Those movements are localized on FK1, FK2, and TPR_1_. The global effect can be represented by a 2D KDE histogram of the distance between the GR and FK1‐FK2 centers of mass (Figure 6f). This plot explicitly shows that removal of DEX widely affects the motion of immunophilins, mainly broadening the explored distances and, at the same time, causing a loss of FKBP51‐GR connection.

Overall, the observed increase in mobility is consistent with the presence of less well‐ordered images for apo GR:Hsp90:FKBP51 complexes in the cryo‐EM particle dataset.

**FIGURE 7:**
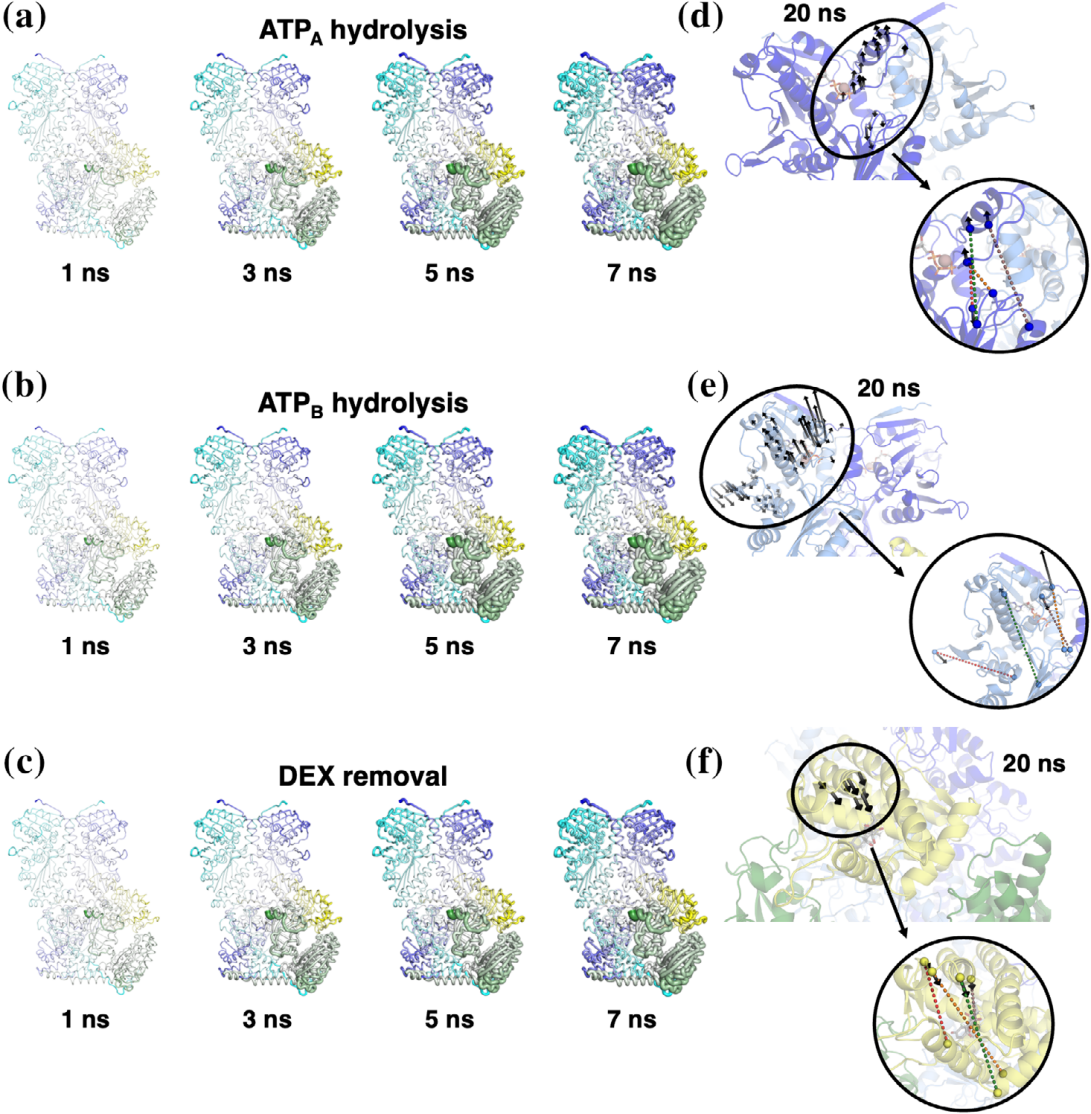
Nonequilibrium dynamics of the Post‐maturation‐FKBP51 complex. (a) D‐NEMD responses to ATP hydrolysis within the NTD_A_ of the Post‐maturation‐FKBP51 + DEX complex after 1, 3, 5, and 7 ns from the perturbation instant. The radius assigned to the putty representation is proportional to the average degree of perturbation felt by each region once the ligand is hydrolyzed. (b) D‐NEMD responses to ATP hydrolysis within the NTD_B_ of the Post‐maturation‐FKBP51 + DEX complex after 1, 3, 5 and 7 ns from the perturbation instant. (c) D‐NEMD responses to DEX (dexamethasone) removal from the GR's active site of the Post‐maturation‐FKBP51 + DEX complex after 1, 3, 5 and 7 ns from the perturbation instant. (d) Direction of the D‐NEMD responses of the Post‐maturation‐FKBP51 + DEX complex upon ATP hydrolysis within the protomer A of Post‐maturation‐FKBP51 + DEX complex after 20 ns from the perturbation. The amplitude and direction of motion were obtained from the analysis of the intra‐complex pairwise Cα distances matrices between the equilibrium and nonequilibrium trajectories after 20 ns. Arrows indicate the average overall direction and magnitude of the captured motion associated with the dynamical response to ATP hydrolysis within the protomer A. In the circle, the magnification of the area interested by the perturbation with arrows indicating the four most relevant amino acids displacements. For a clarified view, dotted lines connect each affected residue with the Cα of the first amino acid ideally intercepted along the direction of motion. (e) Direction of the D‐NEMD responses of the Post‐maturation‐FKBP51 + DEX complex to ATP hydrolysis within the protomer B of Post‐maturation‐FKBP51 + DEX complex after 20 ns from the perturbation. (f) Direction of the D‐NEMD responses of the Post‐maturation‐FKBP51 + DEX complex to DEX removal from the GR of Post‐maturation‐FKBP51 + DEX complex after 20 ns from the perturbation.

Next, we apply D‐NEMD to characterize the dynamical effect arising from ATP hydrolysis and, separately, from DEX removal (Figure 7a–f). In these systems, the hydrolysis‐induced changes reverberate on the same regions as the Maturation + DEX complex, but with different extents (Figure 7a,b). The NTD cross communication is still present, but with a significantly reduced impact. The same occurs with signal transduction towards the GR active site area. The CTDs show an asymmetric accumulation of the allosteric signal, with a stronger effect in CTD_A_ than in CTD_B_. The largest responses involve the entire immunophilin FKBP51, which is affected more than any other region. Similar patterns in dynamic responses are observed for DEX removal (Figure 7c). Indeed, most structural changes are concentrated on FKBP51, with considerably fewer occurring in the other components (NTDs and CTDs).

The “internal” analysis of the D‐NEMD simulations reveals that only small movements are caused by the external perturbations. Indeed, the effects of ATP hydrolysis are concentrated around the active site, remodeling it and favoring phosphate release (Figures 7d,e, S12A–C, and S13A–C). An interesting asymmetric protomer behavior emerges from this analysis. Hsp90_A_ is less affected by hydrolysis, with the largest responses localized on the active site loop (residues 113–133 of protomer A), involving a slight rotation towards the NTD_A_ core (Figures 7d and S12A,B). Conversely, Hsp90_B_ hydrolysis has a wider impact, affecting not only the same active site loop (residues 113–133 of protomer B) but also a long helix close to the ATP active site (residues 54–64) and the modeled charged linker (around residue 220) (Figures 7e and S13A,B). The protomer B motion involves an opening of the active site, which then triggers the displacement of the charged linker. Upon DEX removal from GR, a closure of the GR active site is observed (Figures 7f and S14A–C), mainly involving a loop in the active site region (GR residues 635–640). The remodeling of the DEX binding site is faster and more significant than the one occurring on the nucleotide binding site upon ATP hydrolysis. This is shown in the distance plots, where the initial exponential increase produces a larger effect (Figure S14B).

To summarize, the results above show the remarkable plasticity of the FKBP51 system, both in the presence and absence of DEX, and the tendency of the cochaperone to detach from the client complex, particularly when the ligand is absent. Cochaperone detachment is expected to cause client release in the cytosol for recycling, with a consequent inactivation of the steroid receptor.

### 3.4 Post‐maturation‐FKBP52 + DEX versus post‐maturation‐FKBP52‐DEX

The Post‐maturation‐FKBP52 + DEX structure is part of an alternative pathway for GR activation compared to the Post‐maturation‐FKBP51 + DEX complex. As in the previous assembly, the original Post‐maturation‐FKBP52 + DEX complex contains DEX in the GR active site. Interestingly, our comparative analyses of their dynamics show that the behavior of the FKBP52‐ and FKBP51‐containing simulations differs, despite the two cochaperones' high sequence and structural similarities.

**FIGURE 8:**
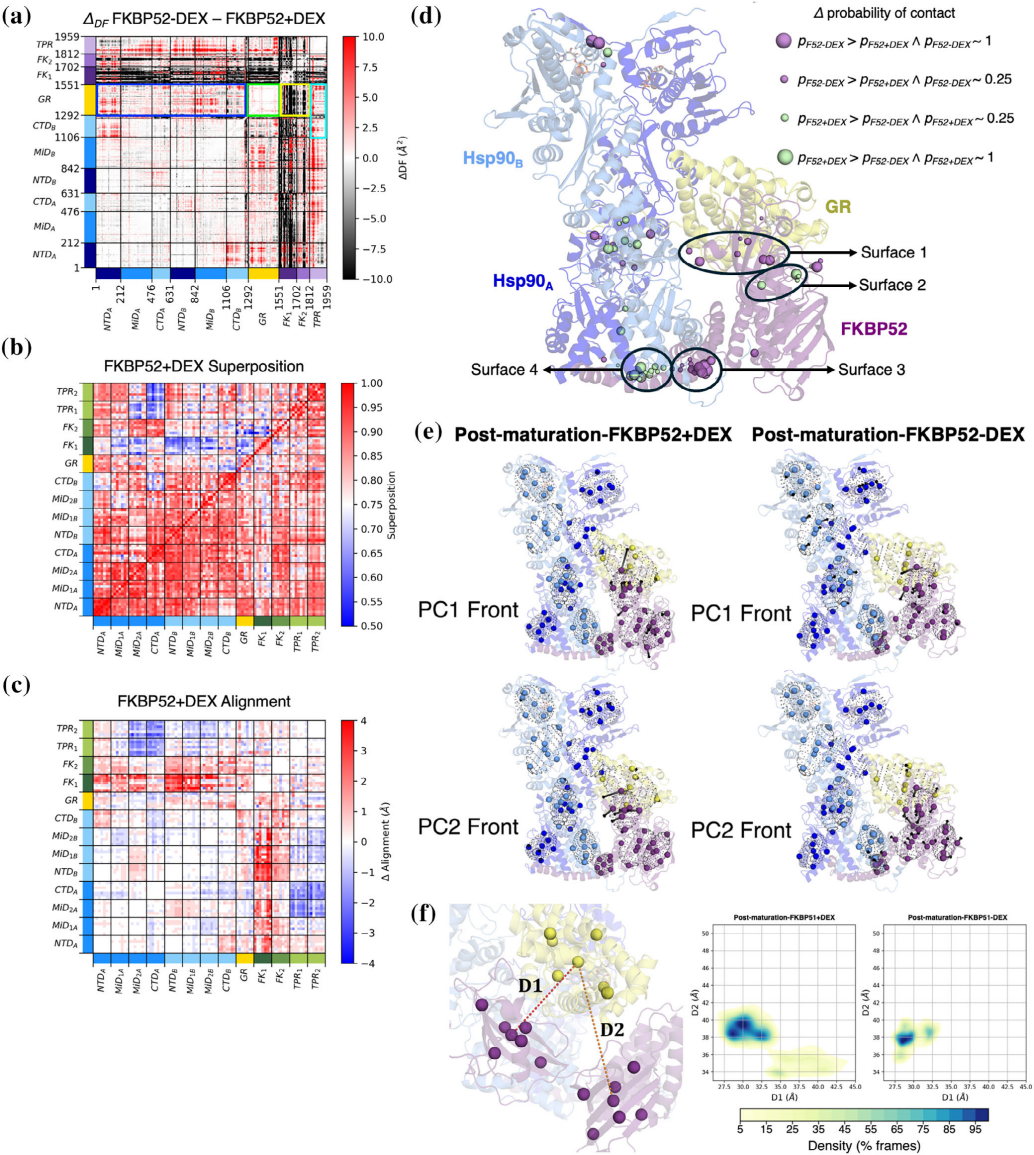
Dynamic analysis of the Post‐maturation‐FKBP52 complex in different ligand states. (a) ΔDF matrix for the Post‐maturation‐FKBP52 complex: Subtraction of the Post‐maturation‐FKBP52 + DEX DF matrix (unperturbed) from the Post‐maturation‐FKBP52‐DEX (perturbed by instantaneous dexamethasone removal). (b) The superposition map contains the overlap integral values for each pairwise distance distribution. The upper diagonal depicts values before superposition, while the lower diagonal represents the values after superposition. Red values represent an overlap of 80% or higher, indicating a similar dynamical behavior between the unperturbed Post‐maturation‐FKBP52 + DEX and perturbed Post‐maturation‐FKBP52‐DEX MDs. White/blue values underlie a non‐optimal superposition, due to the different shapes of the distributions and, thus, a marked dynamical difference. (c) The alignment map represents the Δ value pertaining to the translation with the highest degree of overlap for each reduced point pairwise distance distribution between Post‐maturation‐FKBP52 + DEX and (perturbed) Post‐maturation‐FKBP52‐DEX trajectories. Blue spots (Δ < 0 Å) mean that the perturbation has increased the distance between reduced points, while red spots (Δ > 0 Å) mean the opposite. (d) Projection of differences in probability of contact onto the Post‐maturation‐FKBP52 complex structure. Each sphere indicates the presence of a statistically significant modified interaction between each inter‐protein residue pair contained in the unperturbed and perturbed contact matrices. Spheres mark the midpoint between the two Cα atoms involved in each interaction, identifying the approximate contact location. Contacts more likely in the Post‐maturation‐FKBP52 + DEX complex are shown in green; those more likely in Post‐maturation‐FKBP52‐DEX are in violet. Sphere radius reflects the maximum likelihood (probability) of the corresponding contact. Black rectangles depict the points corresponding to the surfaces discussed in the main text. (e) Projection of the first two PCA eigenvectors (vectors as arrows) onto structures of the Post‐maturation‐FKBP52 complex, from unperturbed (Post‐maturation‐FKBP52 + DEX; left) and perturbed simulations (Post‐maturation‐FKBP52‐DEX; right). Spheres represent the TOG‐derived reduced points, while dots outline the shape of the identified ellipsoids that include each cluster of spheres. (f) On the right, the KDE plot of distances between the center of mass of GR core and centers of mass of FK_1_ (D1) and FK_2_ (D2) domains respectively, in the Post‐maturation‐FKBP52 complex equilibrium simulation with (and without) DEX ligand. Here, the DEX presence tightens the interaction between GR and the immunophilin, evident from the narrower fluctuations along both D1 and D2. On the left, the representation of the investigated distances onto the structure of the Post‐maturation‐FKBP52 complex.

In contrast to what is observed in the FKBP51 case, DEX removal from the FKBP52‐containing complex determines a decrease in GR internal pairwise coordination (Figure 8a; green box) together with an increase in RMSF fluctuations (Figure S3A,B). The TOG analysis (Figure 8b,c) does not display a clear change in pairwise reduced point distance, suggesting that the absence of the ligand primarily affects internal GR coordination rather than the receptor's overall shape.

After DEX removal, the interaction between Hsp90 and GR is slightly modified (different from F51 + DEX simulations). The most interesting effect occurs within the lumen, where the GR tail reorganizes its contact network (Figure 8d). Moreover, the GR core increases its contact probability with two residues in CTD_B_ (residues 602 and 624). These altered interactions produce minor variations in the TOG analysis (Figure 8b,c). Indeed, after DEX removal, the GR‐Hsp90 interface is not majorly altered. Accordingly, the ΔDF matrix identifies few changes in the GR‐Hsp90 coordination network, indicating a minor impact from the absence of DEX (Figure 8a; blue box). The same occurs for Hsp90 inter‐protomer interactions. Consistently, only minor differences in both the contact analysis and the TOG alignment map are observed (Figure 8c,d).

Considering the GR relationship with FKBP52, F52 + DEX simulations generally show tighter coordination than the F52‐DEX ones (Figure 8a; yellow box). Contact analysis highlights four distinct surface reshapings: two involve interactions between GR and FKBP52, and the other two involve the interactions between the CTD_B_ loop and the immunophilin TPR_2_ domain (Figure 8d). The first change is located on the surface between the GR and FK1 domain, with a global increase in interaction probability for all the contacts upon DEX removal. This is confirmed by both the TOG analysis (Figure 8b,c), where the overall distance between reduced points decreases, and by the ΔDF matrix (Figure 8a; yellow box), where the coordination increases after DEX removal. The second surface is localized between the FK1‐FK2 connecting loop and the GR core. Here, the increased contact probability brings the FK2 domain closer to GR (Figure 8c), reflecting a stronger connection between monomers (Figure 8a; yellow box). The remodeling of the other two surfaces supports the TPR_1_ and TPR_2_ domains’ detachment from Hsp90. Indeed, the TPR_2_ terminal helix decreases its contact probability with the lower part of CTD_B_, while enhancing it with the terminal part of the CTD_B_ loop. FKBP52 changes are also observed in the TOG (with TPRs detaching from both the MiD_2_s and CTDs) (Figure 8b,c) and ΔDF matrix (Figure 8a; cyan box) analyses, both suggesting a generalized loss of coordination.

Additionally, the coordination between GR and FKBP52 is significantly lower in the F52 + DEX simulation compared to F52‐DEX, although this difference does not involve the TPR domain (Figure 8a; cyan box). Moreover, the TOG matrix highlights a general increase in the proximity between FKs and Hsp90 (especially NTDs). FKBP52 is less internally mechanically connected (excluding the TPR domain) in the F52 + DEX simulation, and the TOG alignment matrix shows a minor modification of its intra‐protein distances (Figure 8c).

PCA of the TOG‐reduced trajectories captures the global motions (Figures 8e, S8D, and S15). For better visualization, we provided PC movies at the link: https://doi.org/10.5281/zenodo.18267647. For the F52‐containing systems, the principal component projections highlight a difference in the complex after the DEX removal. PC1 in both the original and perturbed systems encode two distinct movements. Firstly, the FK1 domain moves towards GR in the DEX‐bound complex, while it performs a rotational motion in the F52‐DEX system. Then, NTD_A_ and MiD_1B_ domains undergo a joint closing motion that is not visible in the F52 + DEX system. Additionally, PC2 for F52 + DEX displays the same rotational movement mentioned before for PC1 of the F52‐DEX system, while PC2 of F52‐DEX exhibits a dispersed motion around FKs and TPR_1_ domains.

The net effect of FKs on GR after DEX removal is depicted in the KDE plot of distances between centers of mass (Figure 8f). The already identified increased coordination results in a conformational closure of the FKs around GR, locking the receptor and consequently decreasing FKs' mobility.

**FIGURE 9:**
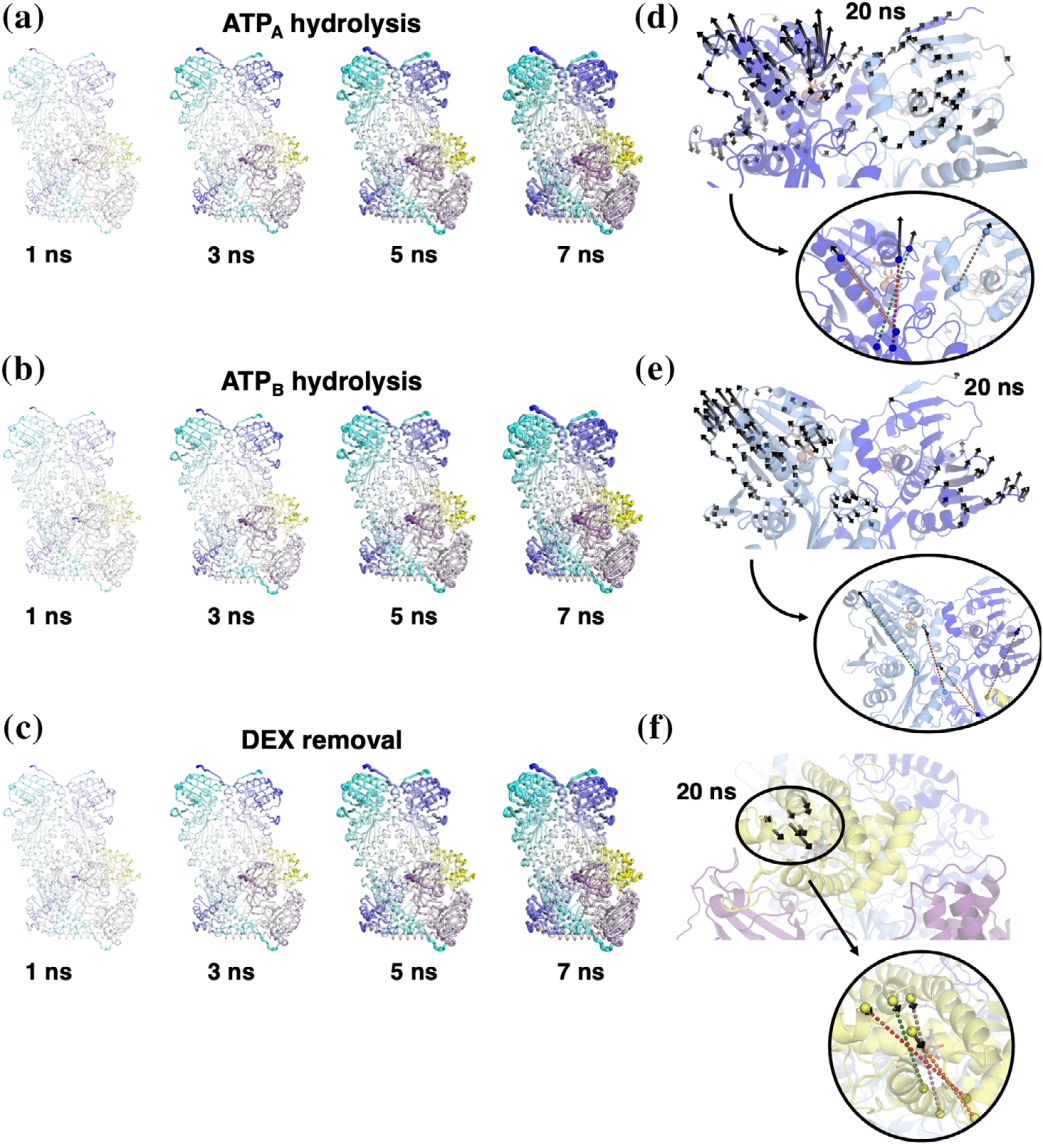
Nonequilibrium dynamics of the Post‐maturation‐FKBP52 complex. (a) D‐NEMD responses to ATP hydrolysis within the NTD_A_ of Post‐maturation‐FKBP52 + DEX complex after 1, 3, 5 and 7 ns from the perturbation instant. The radius assigned to the putty representation is proportional to the average degree of perturbation felt by each region once the ligand is hydrolyzed. (b) D‐NEMD responses to ATP hydrolysis within the NTD_B_ of Post‐maturation‐FKBP52 + DEX complex after 1, 3, 5 and 7 ns from the perturbation instant. (c) D‐NEMD responses to DEX (dexamethasone) removal from the GR's active site of Post‐maturation‐FKBP52 + DEX complex after 1, 3, 5 and 7 ns from the perturbation instant. (d) Direction of the D‐NEMD responses of the Post‐maturation‐FKBP52 + DEX complex to ATP hydrolysis within the protomer A of Post‐maturation‐FKBP52 + DEX complex after 20 ns from the perturbation. The amplitude and direction of motions were obtained from the analysis of the intra‐complex pairwise Cα distances matrices between the equilibrium and nonequilibrium trajectories after 20 ns. Arrows indicate the average overall direction and magnitude of the motions associated with the dynamical response to ATP hydrolysis within the protomer A. In the circle, the magnification of the area interested by the perturbation with arrows indicating the four most relevant amino acids displacements. For a clarified view, dotted lines connect each affected residue with the Cα of the first amino acid ideally intercepted along the direction of motion. (e) Direction of the D‐NEMD responses of the Post‐maturation‐FKBP52 + DEX complex to ATP hydrolysis within the protomer B of Post‐maturation‐FKBP52 + DEX complex after 20 ns from the perturbation. (f) Direction of the D‐NEMD responses of the Post‐maturation‐FKBP52 + DEX complex to DEX removal from the GR of Post‐maturation‐FKBP52 + DEX complex after 20 ns from the perturbation.

As for the complexes above, we have used D‐NEMD to identify the dynamic response of the FKBP52 systems to ATP hydrolysis from both the NTD active sites and to DEX removal from GR. Upon ATP hydrolysis, the regions affected largely overlap with those described above. Specifically, the cross‐talk between the NTDs is accompanied by communication with the GR active‐site region, CTD_A_, CTD_B_ (to a lesser extent, as in the Post‐maturation‐FKBP51 + DEX complex), and with the whole immunophilin FKBP52 (although the latter is substantially weaker than in the Post‐maturation‐FKBP51 + DEX system) (Figure 9a,b).

DEX removal induces changes in behavior that are similar to those of ATP hydrolysis, with the structural responses from ligand removal affecting the same regions (Figure 9c).

Also, for this system, the “internal” analysis of the D‐NEMD simulations reveals that the effects of ATP hydrolysis remain confined to the regions around the active sites. Hydrolysis within the NTD_A_ produces strong local responses, affecting almost the entire domain, with a rotational movement that opens the space for HPO_4_^2−^ release (Figures 9d,e, S16, and S17). The most affected regions are the active site loop (residues 113–133), the long helix forming the ATP binding site (residues 54–64), and the modeled charged linker (around residue 220), which tend to move away from their starting positions. Some structural effects are transmitted to protomer B, although to a lesser extent (Figure S16B), with a maximum pairwise atomic displacement of ~1 Å after 20 ns. ATP hydrolysis in protomer B induces an active site response that propagates in a manner similar to that observed for protomer A, with an even stronger influence on the modeled charged loop (Figures 9e and S17A,B). Overall, the regions involved in the response to hydrolysis are the same as previously identified, with the addition of a displacing effect on the MiD_B_ loop in the Hsp90 lumen (residues 394–405) towards CTD_B_. The results from instantaneous DEX removal align closely with the previous F51 + DEX simulations (Figures 9f and S18), exhibiting the same closing motion of the GR active‐site loop (residues 635–640).

In summary, the Post‐maturation‐FKBP52 + DEX complex displays greater internal coordination and overall stability compared to the Post‐maturation‐FKBP51 + DEX system. Notably, while ligand absence does not markedly alter Hsp90 dynamics, it appears to stabilize cochaperone–client interactions further. The extensive communication observed between the assembly and the GR active site in FKBP52 systems suggests that this complex promotes a steroid‐binding–competent pre‐organization of GR. This may enhance ligand affinity, stabilize the chaperone complex, and facilitate efficient GR translocation to the nucleus via dynein.

Like FKBP51, FKBP52 emerges as a highly responsive component of the machinery, consistent with a model in which rapid detachment and substitution of immunophilins enable adaptation to changing cellular demands.

### 3.5 Post‐maturation‐FKBP51 + DEX versus post‐maturation‐FKBP52 + DEX

**FIGURE 10:**
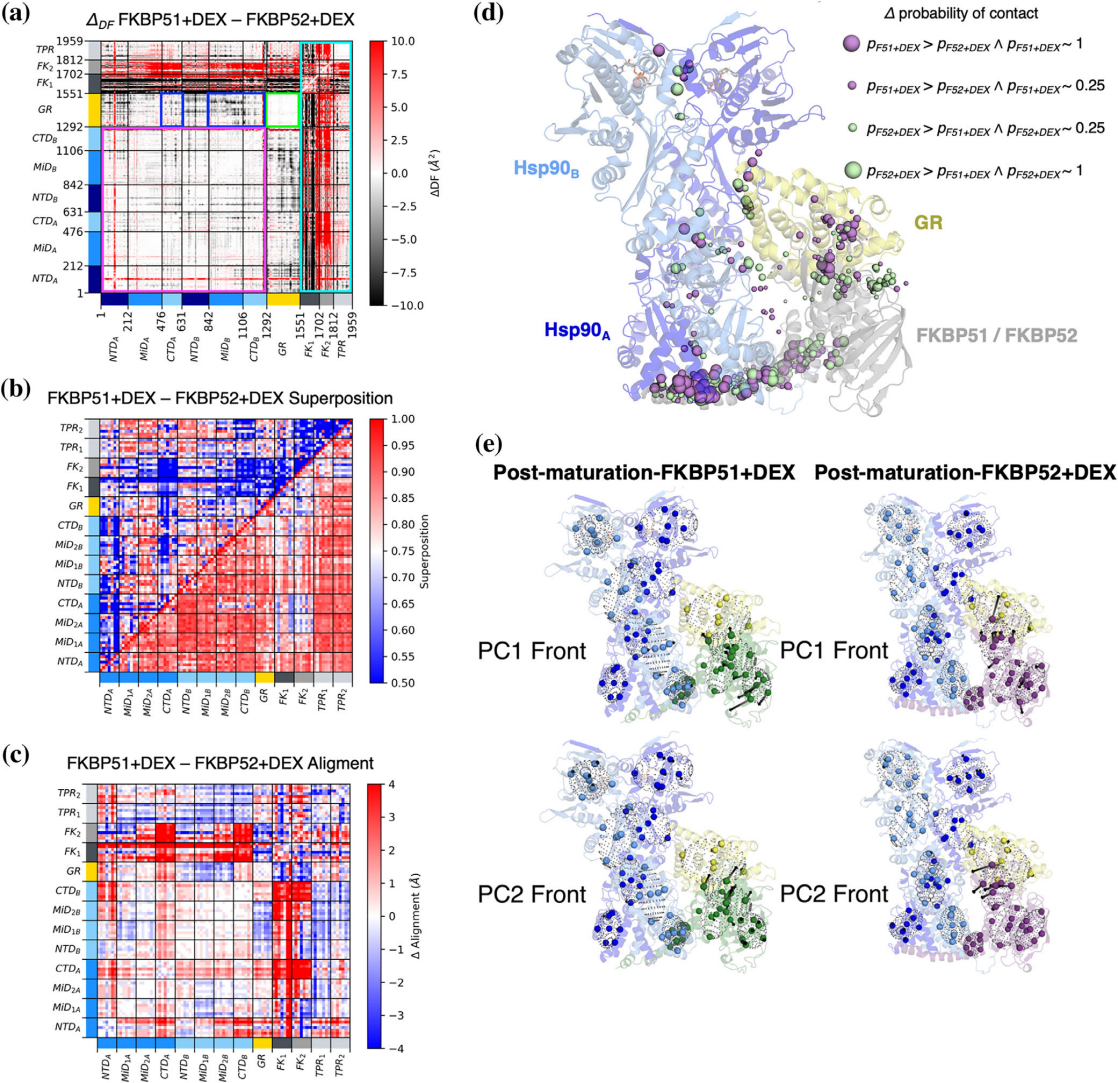
Differential dynamics of the two DEX‐containing immunophilin complexes. (a) ΔDF matrix for the Post‐maturation‐FKBP52 + DEX—Post‐maturation‐FKBP51 + DEX complex: Subtraction of the Post‐maturation‐FKBP52 + DEX DF matrix from the Post‐maturation‐FKBP51 + DEX. Red color indicates a gain of coordination for the FKBP52‐containing complex (or a loss of coordination for the FKBP51‐containing structure), while the black color indicates the opposite. (b) The superposition map contains the overlap integral values for each pairwise distance distribution. The upper diagonal depicts values before superposition, while the lower diagonal represents the values after superposition. Red values represent an overlap of 80% or higher, indicating a similar dynamical behavior between the Post‐maturation‐FKBP51 + DEX and Post‐maturation‐FKBP52 + DEX MDs. White/blue values underlie a non‐optimal superposition, due to the different shapes of the distributions and, thus, a marked dynamical difference. (c) The alignment map represents the Δ value pertaining to the translation with the highest degree of overlap for each reduced point pairwise distance distribution between Post‐maturation‐FKBP51 + DEX and Post‐maturation‐FKBP52 + DEX trajectories. Blue spots (Δ < 0 Å) mean an increased distance between reduced points, while red spots (Δ > 0 Å) mean the opposite. (d) Projection of contact probability differences (Post‐maturation‐FKBP51 + DEX vs. Post‐maturation‐FKBP52 + DEX) onto the Post‐maturation‐FKBP52 complex structure (as reference). Each sphere indicates the presence of a relevantly modified interaction between each inter‐protein residue pair contained in the two contact matrices. Spheres mark the midpoint between the two Cα atoms involved in each interaction, identifying the approximate contact location. Contacts more probable in the Post‐maturation‐FKBP52 + DEX complex are shown in green; those more probable in Post‐maturation‐FKBP51 + DEX are in violet. Sphere radius reflects the maximum probability of the corresponding contact. (e) Projection of the first two PCA eigenvectors (vectors as arrows) onto the relative structures of the immunophilin complex, from Post‐maturation‐FKBP51 + DEX (left) and Post‐maturation‐FKBP52 + DEX (right) simulations. Spheres represent the TOG‐derived reduced points, while dots outline the shape of the identified ellipsoids that include each cluster of spheres.

The behavior of immunophilins FKBP51 and FKBP52 is still enigmatic in the regulation of GR folding and Hsp90 chaperoning cycle (Noddings et al., 2023). To better compare the effects of the presence of immunophilins FKBP51 and FKBP52 on the internal dynamics of the two complexes, we analyze the differences in DF matrices for F51 + DEX and F52 + DEX simulations (Figure 10a) together with the other analyses explained above. For these systems, the TOG analyses are less comparable than previously described complexes due to their sequence differences. Indeed, the different proteins involved cause intrinsic variations in the system's dynamics, leading to distinct ensembles of internal distances and, consequently, to different atomic selections across models, which is already an indication of substantial underlying dynamic differences for the client in the two complexes. Nevertheless, GR (which is in the folded state) displays similar coordination patterns and internal distances distributions (Figure 10b,c). GR shows a coordination increase that is widespread throughout the protein in the F51 + DEX simulations compared to F52 + DEX (Figure 10a; green box). Moreover, TOG analysis highlights GR's different motions around Hsp90. Indeed, in the presence of FKBP51, GR tends to stay closer to the CTDs, but it moves apart from the MiD_B_ domains (Figure 10b,c). The receptor increases its coordination with both the mentioned Hsp90 domains (Figure 10a; blue boxes). The contact map shows differences in the contact probability between the GR tail loop with both the lumen and Hsp90_A_ MiD domain. However, a global trend of increased/decreased number of contacts cannot be easily established (Figure 10d).

Moreover, Hsp90 gains internal coordination when FKBP51 is bound (Figure 10a; pink box), generally favoring protomers to approach each other. MiD domains, however, tend to move apart, partially deforming the client binding site (Figure 10b,c). The Hsp90 active site of protomer A shows a markedly opposite behavior, being strongly coupled to all the protein components in Post‐maturation‐FKBP52 + DEX simulations, including GR.

The FKBP portion shows the strongest differences in ΔDF between the two simulations (Figure 10a; cyan box). There is a clear distinction in coordination for the FK_1_ and the FK_2_ immunophilin domains. Indeed, FK_1_ shows stronger coordination with Hsp90 and GR in F51 + DEX with respect to F52 + DEX. On the other hand, FK_2_ displays greater coordination with other complex members in the F52 + DEX simulation. This trend suggests that the distinct pattern in coordination for FKBPs domains could be linked to the distinct actions that occur in the two complexes. This change in coupling is also evident in the TOG analysis (Figure 10b,c). The superposition of FKs with the other compact domains remains poor even after the alignment (Figure 10c). Regarding the immunophilins, contact analysis indicates a stronger interaction between the FK1 domain and GR in the F51 + DEX system compared with F52 + DEX. The opposite stands for the FK_2_ domain, which tends to stay closer to GR when FKBP52 is present. This effect may be mediated by an extended patch of increased interactions located near the interface between GR and FK_2_ (Figure 10d). Regarding TPRs, a complex pattern of interaction probability with the long CTD_B_ disordered loop is evident. In general, due to differences in sequence, the two immunophilins behave differently, with a slight tendency of FKBP52 TPRs to stay closer to the CTD_B_ of Hsp90. PCA of TOG points (Figures 10e and S19) and related movies (https://doi.org/10.5281/zenodo.18267647) confirm this observation and identify the main behavior difference in the FK domains. Their motions differ in both direction and amplitude. Interestingly, FK_1_ moves towards GR in F51 + DEX and F52 + DEX, while also approaching the MiD_B_ domain in F52 + DEX. FK_2_ shows a broader structural change in the Post‐maturation‐FKBP51 + DEX system compared with the Post‐maturation‐FKBP52 + DEX one.

D‐NEMD analyses of the Post‐maturation‐FKBP51 + DEX and Post‐maturation‐FKBP52 + DEX assemblies uncover important differences, evident under both ATP hydrolysis and DEX‐removal perturbations (Figures 7 and 9). Across the two D‐NEMD perturbations, a common pattern in dynamic responses emerges, with remarkable differences involving the immunophilin proteins. They share an evident communication network that involves NTDs, the GR active site area, the CTD of protomer B, and the relative immunophilin. The difference in the systems' responses mainly concerns the extent of their connection with the immunophilins. For Post‐maturation‐FKBP51 + DEX, FKBP51 shows a strong accumulation of the structural responses across its entire structure. The structural changes to the perturbations also diffuse into FKBP52, but to a lesser extent. Interestingly, the allosteric signal is not equally distributed on the immunophilin, being more concentrated on its FK_1_ domain. This difference in behavior highlights the diverse biological relevance of the two alternative steps of the GR activation cycle. For the Post‐maturation‐FKBP51 + DEX, the observed role of the chaperone complex is to keep GR in the cytosol and release it to recycle it back and restart the cycle: the extended and efficient communication network of the immunophilin with the other hotspots in the assembly could facilitate this process, making FKBP1 poised to respond to nucleotide changes or other variations in Hsp90 or in the client ligand state.

In the Post‐maturation‐FKBP52 + DEX complex, the GR must be translocated to the nucleus. Here, the restrained response of Hsp90 to ligand binding/unbinding may be critical for maintaining GR in the proper orientation for efficient steroid binding, subsequent recognition by dynein, and eventual dimerization, rather than for promoting release from the chaperone assembly. Analyses of D‐NEMD following DEX removal highlight key differences between the two immunophilins: FKBP52 maintains GR in a conformationally locked, binding‐prone state, consistent with a role in preorganizing the receptor for transport and activation. Additionally, D‐NEMD reveals divergent behavior upon ATP hydrolysis, with the Hsp90_A_ active site in the Post‐maturation‐FKBP51 + DEX assembly being less perturbed by phosphate cleavage, suggesting a more catalytically competent state. Together with NTD_A_‐derived DF scores (Figure S20), these findings suggest that FKBP51 promotes more efficient ATP turnover, enhancing catalytic activity and ultimately favoring GR release into the cellular environment.

### 3.6 MiD–GR distances

We compare the distances between the centers of mass of the MiDs and that of GR (excluding the unfolded tail loop), revealing how cochaperone engagement shapes receptor positioning. Analysis of MiD_B_–GR distances (Figure S5C) provides a coarse, yet biologically meaningful, metric of Hsp90's stabilizing influence. Simulations of the original cryo‐EM structures show a clear trend: the Loading‐DEX complex exhibits the smallest mean distance, followed by the Maturation + DEX and then the Post‐maturation + DEX assemblies, indicating a progressive increase in inter‐domain separation as the chaperone cycle advances. This trend reflects the sequential actions of cochaperones, with no direct interaction in the loading stage; stabilization by Hsp90 closure and the p23 tail helix during maturation; and further displacement upon immunophilin binding, as the FK1 domain inserts between Hsp90_B_ and GR.

Notably, the two Post‐maturation + DEX complexes diverge in line with their distinct functions. The Post‐maturation‐FKBP51 + DEX‐containing system, responsible for GR release into the cytoplasm, displays the largest mean distances, consistent with loosening of GR contacts. By contrast, the FKBP52 + DEX‐containing complex, which mediates nuclear translocation, shows a broader distance distribution with a lower mean value, in some cases overlapping with the Maturation + DEX state, consistent with tighter retention of GR during transport.

Analyses of systems perturbed by either insertion or removal of DEX into the original models reinforce these trends. Adding DEX to the Loading‐DEX complex increases GR fluctuations, favoring conformations compatible with progression to Maturation + DEX. Conversely, DEX‐removal (in the Maturation‐DEX complex) narrows the distance distribution and shifts it towards Loading‐like configurations. Similar ligand‐dependent effects are observed in both post‐maturation states. Together, these results highlight how changes in ligand conditions are directly encoded in the spatial relationship between GR and its chaperone partners, shaping the progression of the folding and activation cycle.

### 3.7 Comparison of simulation results with experimental characterization of points of regulation

Overall, the simulations above enable us to identify changes in dynamic states induced by distinct ligands and conditions that reshape biologically important regions of the assemblies. Interestingly, comparison with literature data shows that the regions identified (vide infra) are highly regulated by key Post‐Translational Modifications (PTMs) (Backe et al., 2020; Truman et al., 2021). Such covalent modifications introduce a further level of control over the mechanisms of chaperone assemblies, likely shaping the complex interplay between different sites and the functional hierarchy involved. In general, the presence of PTMs defines a chaperone code, extending the folding assembly's capability to respond to changing conditions and stresses. Thanks to improvements in analytical and proteomic technologies, a large number of PTMs have in fact been uncovered on chaperones, including phosphorylation, acetylation, methylation, SUMOylation, and ubiquitination (Truman et al., 2021).

Specifically, DEX presence/absence reshapes the GR‐Hsp90 contact surface in a protein‐assembly‐specific fashion. This particular GR surface contains the only known SUMOylation site in the Ligand Binding Domain (LBD) (K703) and promotes receptor degradation (Hua et al., 2016). In the Maturation Complex (where the fate of GR is still unknown) and in the Post‐maturation‐FKBP51 system (where GR cytosolic recycling is promoted), the contact probability redistribution strongly affects this region. In contrast, for the Post‐maturation‐FKBP52 assembly, which promotes nuclear translocation of active (mature) GR, a modification in the LBD binding site does not perturb the GR‐Hsp90 binding area. Together, these observations may suggest a looser protein interaction in the former case, evolved to facilitate GR degradation, and a tighter interaction in the latter case, when the GR activity is required in the cell nucleus, and the receptor needs to be protected from further modifications (Oakley & Cidlowski, 2011).

Furthermore, K38 phosphorylation in yeast Hsp90 is known to impact cell growth, chaperoning of steroid hormone receptors, and interaction profiles with cochaperones Hop and p23. This residue lies at the interface between the two NTDs of Hsp90. In all models used in this work, except for the post‐maturation‐FKBP52 system, perturbation of the GR ligand‐binding pocket induced by DEX addition or removal results in remodeling of the NTD_A_‐NTD_B_ interface relative to the equilibrium simulations: K38 is a key part of this interface. This suggests that the receptor's active site is involved in dynamic cross‐talk with the NTD‐NTD interface at the K38 position, thereby modulating interactions with cochaperones and Hsp90 function (Backe et al., 2025).

Another important modification of key residues is the methylation of the conserved K594 of yeast yHsp90 (K615 in human Hsp90ɑ) (Rehn et al., 2020), which is known to alter ATP hydrolysis, p23 binding and GR client regulation. The surface contact probability highlights interesting results for this region: in the Loading complex, after adding DEX, there is no significant network remodeling around K615; however, in the Maturation complex, GR ligand removal induces a structural response at the Hsp90_A_‐Hsp90_B_ interface around K615. Similarly, even in the FKBP51‐containing complex, GR perturbation promotes remodeling involving the portion of Hsp90_A_ near K615 and the GR loop within the chaperone lumen (Backe et al., 2025). These observations are consistent with the region of K615 becoming allosterically involved only in the later maturation stages and becoming functionally relevant as a function of the ligand and co‐chaperone make‐up of the assembly.

In addition to PTMs, we also probed the allosterically connected regions identified above by mapping mutations that are thought to affect function and are reported in pathological disorders. In the GR, the T556I mutation is reported to disrupt a pivotal hydrogen bond that affects the receptor's 3D structure, impairs ligand affinity, cytoplasmic‐to‐nuclear translocation, and transactivation of glucocorticoid‐responsive genes (Nicolaides et al., 2016). A similar or even stronger effect is observed for the I559N (Kino et al., 2001) and F737L substitutions (Charmandari et al., 2007). All three sites lie in the same GR region, close to the GR‐binding pocket of the LBD and on the opposite side of the Hsp90‐GR interface.

Importantly, our analyses clearly identify this region as a hotspot of the assemblies' allosteric networks. This is primarily reflected in differences in contact probability in the Loading system when DEX is present or absent in the GR‐binding pocket. Indeed, a significant remodeling of interactions occurs at the interface between T556 and Hsp90's MiD of protomer B (residues 602–604 and 627–628). Importantly, D‐NEMD captures these effects in detail; for example, examining GR extraction from the Post‐Maturation‐FKBP51 + DEX and Post‐Maturation‐FKBP52 + DEX assemblies shows a pronounced buildup of structural responses within the regions identified by the mutations above. Furthermore, the responses triggered by ATP hydrolysis in the Hsp90 active sites consistently travel toward the terminal GR region across all models, suggesting that it is a hotspot for Hsp90‐GR communication. A particularly noteworthy hotspot is the T773C mutation, known to impair both the signal transduction and ligand‐binding affinity (Charmandari et al., 2005). Although located at the C‐terminal end of GR in our models, it lies close to the p23‐tail‐helix in the Maturation complex, where pronounced remodeling of the GR‐p23 interface was observed in D‐NEMD.

## 4 DISCUSSION

Multicomponent machineries are increasingly recognized as the key determinants of biological function (Gestwicki, 2022; Johnson & Gestwicki, 2022; Sabbagh et al., 2018; Sali, 2021). Parts of the assemblies can act as enzymes whereas others can be kinetic modulators of reactions, binders of ligands, adapters that regulate substrate selection, or signaling factors that determine subcellular localization and turnover. In this framework, overall function emerges through the collaborative action of all components, while control over it depends on the detailed composition of each complex.

In many cases, functional assemblies can be represented as modular networks in which hub proteins connect and integrate different biomolecules from distinct pathways. The Hsp90 chaperone machinery is a paradigmatic example: Hsp90 acts as a central hub, and variation in the composition of interactors around it engenders the emergence of context‐specific functions in response to specific cellular conditions (Laine & Carbone, 2017). Conformational dynamics (Nussinov et al., 2021) is one of the factors that allow Hsp90 and its structural motifs to be used in multiple contexts, facilitating the binding and unbinding of specific cochaperones and substrates at different stages of the chaperone cycle (Mader et al., 2020; Sahasrabudhe et al., 2017; Schmid & Hugel, 2020; Schopf et al., 2017; Sohmen et al., 2023; Wolf et al., 2020). The adaptability, plasticity, and versatility of the biological components of multi‐protein machineries are common traits of central cellular complexes, such as the ribosome (Ferguson et al., 2015), the proteasome (Tanaka, 2009), or the spliceosome (Pokorná et al., 2025).

Understanding how local modifications, such as switching interaction partners or binding small ligands, can trigger and regulate functionally‐oriented motions in these large protein assemblies and shedding light on the mechanisms underlying their adaptability and response to different conditions is a major emerging issue in structural and chemical biology (Nussinov et al., 2019; Wodak et al., 2019).

In this study, we investigate the molecular mechanisms underlying the folding and activation of the GR (Chen et al., 2023; Dahiya et al., 2022; Noddings et al., 2022; Noddings et al., 2023; Sabbagh et al., 2018; Wang et al., 2022), a prototypical client of the Hsp90 chaperone machinery. To this end, we integrate equilibrium and D‐NEMD simulations. Comparative analyses are performed across a series of Hsp90–cochaperone–client assemblies, in the presence of their physiological ligands—nucleotides at the chaperone active sites and a steroid within the GR ligand‐binding pocket—and under perturbed conditions. In the latter, the steroid DEX was either introduced into the ligand‐binding domain of GR in the Loading‐DEX complex, where it is absent in the corresponding cryo‐EM structure, or removed from the Maturation + DEX complex and Post‐maturation + DEX assemblies with FKBP51 and FKBP52, where it is bound in the original cryo‐EM structures.

To analyze the dynamics of these multicomponent systems, we introduce novel analysis approaches that, starting from atomistic‐resolution descriptions, efficiently reveal the rigid‐body movements of the partners or their regulatory subdomains. The results reveal exquisite plasticity in all complexes and in the specific substructures of their components, helping to shed light on possible mechanistic differences in the regulation of GR post‐maturation by the two highly similar immunophilins FKBP51 and FKBP52. The activation cycle of the GR is initiated by the formation of the Loading‐DEX complex. In this state, DEX binding to the receptor's binding site promotes the release of Hop and Hsp70 cochaperones, thereby facilitating a tighter closure of Hsp90 around its central lumen and enhancing Hsp90 intramolecular coordination. Notably, the cochaperones exhibit dynamic features that appear predisposed to such motions even in the Loading‐DEX complex, where DEX is absent.

Steroid binding is revealed as a critical determinant of Maturation + DEX complex stability, with DEX reinforcing the interaction network that bridges GR with the Hsp90–p23 system. Removal of the steroid molecule disrupts this connectivity, with the most pronounced effects observed in the GR‐bound C‐terminal α‐helical tail of p23. This heightened sensitivity suggests that p23 may act as a molecular sensor of client occupancy, selectively recognizing ligand‐engaged substrates as competent for progression while diverting states that are not prone to bind towards stalling or alternative outcomes. Such a mechanism would position p23 as a regulatory node in the Hsp90 cycle, capable of integrating client‐ and ligand‐dependent cues to help dictate chaperone progression.

The combination of equilibrium MD and D‐NEMD simulations delineates the mechanistic basis for the divergent behaviors of the Post‐maturation + DEX complexes containing Hsp90, DEX‐bound GR, and either FKBP51 or FKBP52. Although structurally similar in the cryo‐EM structures, the two immunophilins exert strikingly distinct functional influences. FKBP52 enhances GR binding and nuclear translocation in vivo, whereas FKBP51 promotes cytosolic release. Consistent with these roles, our simulations reveal that FKBP51 and FKBP52 establish different coordination patterns through their FK domains, thereby impacting distinct GR interfaces. D‐NEMD further demonstrates that FK domains act as allosteric hubs, integrating signals derived from ATP hydrolysis in Hsp90 or ligand removal from GR. Notably, FKBP51 is more responsive to the external triggers, leading to distinct remodeling of the GR–immunophilin interface, particularly through dynamic interactions involving the FK1 and FK2 substructures. These differential responses extend to the regulation of Hsp90 itself. FKBP51 stabilizes the ATP‐binding pocket of Hsp90 protomer A, favoring a catalytically competent state that may accelerate ATP hydrolysis and thereby facilitate GR release in the cytoplasm (Noddings et al., 2023). By contrast, FKBP52 appears to restrain ATPase activity, maintaining GR in a ligand‐competent configuration while loosening GR–complex protein–protein interactions (Noddings et al., 2023). Such a less‐activated state could favor conformational exploration and broaden the conformational space accessible to GR, exposing surfaces required for dynein engagement and nuclear translocation.

Together, these findings suggest that FKBP paralogs act not merely as accessory factors but as selective modulators of the Hsp90 chaperone cycle, fine‐tuning client fate through allosteric mechanisms. This functional bifurcation underscores an evolutionary strategy in which closely related cochaperones diversify client outcomes by rewiring interaction networks.

Interestingly, asymmetric regulation of Hsp90 enzymatic activity is consistently observed in overall complex dynamics analysis, in the remodeling of interaction contacts, and in the reorganization of the nucleotide‐binding site upon phosphate release or DEX addition/removal.

ATP hydrolysis in Hsp90 is known to be sequential: we indeed observe distinct dynamic states of Hsp90 protomers that depend on ligand conditions and client/co‐chaperone context. In the maturation and FKBP52‐containing complexes, ATP remains stably coordinated to Mg^2+^ through all three phosphate groups during the simulations (Figure S21). Conversely, in the FKBP51‐containing complex, ATP shows reduced Mg^2+^ coordination stability and repeatedly shifts toward a two‐phosphate coordination mode (Figure S21). We, therefore, interpret the FKBP51 context as destabilizing the canonical ATP‐Mg^2+^ coordination geometry, which may be relevant for hydrolysis. At the same time, in the Post‐maturation‐FKBP51 complex, the effects on protomer A due to ATP_A_ hydrolysis are significantly more limited than the displacements caused on protomer B by the ATP_B_ reaction. In contrast, for the Post‐maturation‐FKBP52 complex, the inter‐protomer response is significantly less pronounced and affects only the directions of the displacements.

The bound GR and cochaperones establish preferential contacts with one of the two protomers of Hsp90. Based on our results, we propose a model for the chaperone cycle in which asymmetry cooperates with the modular assembly mechanism to expand the space of configurations and dynamic states displayed by Hsp90 during client interaction. This emerging structural complexity in the assembly and regulation of the chaperone machinery that recruits the client may help provide selectivity for the substrate and directionality to client‐remodeling reactions. This is exemplified here by the cases of FKBP51 and FKBP52.

As a caveat, we note that our simulations, based on a classical‐physics representation of different nucleotide states, are not conclusive in identifying the Hsp90 protomer that hydrolyzes ATP first. A full clarification of this would require a complete QM/MM investigation to extract the reactivity and energetic features that drive hydrolysis, while simultaneously computing the energy differences for reactive poses from our classical MD trajectories.

Finally, our findings here underscore the previously unexplored role of client ligands as active participants in chaperone‐mediated folding. In the case of GR, steroid binding not only stabilizes distinct chaperone assemblies but also modulates the allosteric communication within the Hsp90 machinery. This suggests a direct link between cellular metabolism and chaperone function, whereby fluctuations in metabolite levels can dynamically influence client maturation and fate. For instance, we suggest a mechanism in which excess of steroids could promote premature engagement of the Loading‐DEX complex, enhance stability of the Maturation + DEX complex, and bias FKBP52 sensing toward nuclear translocation. Indeed, a recent study provided experimental confirmation of DEX's influence on GR expression by investigating, in vitro, the effects of DEX treatment on airway smooth muscle cells (ASMC) derived from patients with chronic obstructive pulmonary disease (Zhou et al., 2024). In previous studies, GR levels in HeLa cells were shown to be dependent on the type and amount of steroid being used to treat them (Shimojo et al., 1995). Additionally, different steroids were shown to induce distinct GR conformations and subcellular dynamics (Vicent et al., 2002), with specific ligand‐dependent conformational changes altering how tightly GR was retained on chromatin (Vicent et al., 2002). Overall, ligands can induce specific GR conformations, affecting its stability, the amount of GR bound to target promoters, interactions with DNA and co‐factors, thereby tuning the transcriptional response in the long run (Hadley et al., 2011; Vicent et al., 2002).

More broadly, our results are consistent with a model in which chemical entities and metabolites act as structural encoders of chaperone regulation, coupling chemical cues to the assembly and remodeling of protein‐folding complexes. Beyond elucidating GR regulation, cochaperones emerge as dynamic checkpoints whose specific actions and dynamics may be targeted to selectively bias client fate, highlighting new opportunities for therapeutic intervention in proteostasis‐related pathologies.

## 5 CONCLUSIONS

The biological function of multicomponent protein machineries emerges from a delicate balance of enzymatic activities, recognition events and the assembly of finely tuned, context‐dependent complexes. This interplay favors the adaptation of key molecular machineries to changing cellular conditions. Our integrative simulations reveal how these principles operate in the Hsp90–GR system at different stages of substrate maturation. We show that nucleotides and cochaperone identity actively model the dynamic states and the allosteric landscape of the chaperone cycle. Importantly, ligand binding in the client on their path to full maturation, exemplified in this work by steroids, emerges for the first time as an important modulator of complex stability and progression. At the same time, the closely related immunophilins FKBP51 and FKBP52 rewire interaction networks to bias GR fate toward cytosolic release or nuclear translocation. Within this model, asymmetric dynamics and sequential ATPase regulation further expand the repertoire of dynamic states accessible to Hsp90, enhancing its ability to adapt to the client, while co‐chaperone regulation imparts selectivity and directionality to client remodeling by differentially interfering with the distinct allosteric pathways.

The integration of chemical and structural information suggests a new model in which complex chaperone machineries are regulated by the cross‐talk among their different components. Ligands (nucleotides or client‐substrates) encode chemical information that translates into the modulation of the stability and functionally oriented dynamics of client‐maturation complexes, while cochaperone recruitment defines selectivity in substrate recognition and directionality in its processing. Although future experiments are required to precisely define the progression of conformational states and the roles of the metabolites shown here, our findings provide a new hypothesis for how the chaperone machinery controls client fate.

## AUTHOR CONTRIBUTIONS

**Andrea Magni:** Investigation; methodology; validation; visualization; writing – review and editing; writing – original draft. **Giorgio Bonollo:** Investigation; writing – original draft; methodology; validation; visualization; writing – review and editing. **Gauthier Trèves:** Investigation; writing – original draft; validation; visualization. **Francesco Frigerio:** Investigation; writing – original draft; resources. **Fabrizio Cinquini:** Investigation; writing – original draft; resources. **Silvia Pavoni:** Investigation; writing – original draft; resources. **A. Sofia F. Oliveira:** Investigation; writing – original draft; methodology. **Adrian J. Mulholland:** Investigation; writing – original draft; methodology. **Stefano A. Serapian:** Investigation; writing – original draft; writing – review and editing; methodology; validation; visualization; formal analysis; supervision. **Giorgio Colombo:** Conceptualization; investigation; funding acquisition; writing – original draft; methodology; writing – review and editing; validation; visualization; formal analysis; project administration; supervision; resources.

## Supporting information


**Data S1:** Supporting Information.

## Data Availability

The data that support the findings of this study are openly available in Zenodo at https://doi.org/10.5281/zenodo.18267647. All code used in this study is publicly available at: https://github.com/colombolab.

## References

[pro70543-bib-0001] Albakova Z , Mangasarova Y , Albakov A , Gorenkova L . HSP70 and HSP90 in cancer: cytosolic, endoplasmic reticulum and mitochondrial chaperones of tumorigenesis. Front Oncol. 2022;12:829520. 35127545 10.3389/fonc.2022.829520 PMC8814359

[pro70543-bib-0002] Ali MMU , Roe SM , Vaughan CK , Meyer P , Panaretou B , Piper PW , et al. Crystal structure of an Hsp90‐nucleotide‐p23/Sba1 closed chaperone complex. Nature. 2006;440(7087):1013–1017. 16625188 10.1038/nature04716 PMC5703407

[pro70543-bib-0003] Allnér O , Nilsson L , Villa A . Magnesium ion–water coordination and exchange in biomolecular simulations. J Chem Theory Comput. 2012;8(4):1493–1502. 26596759 10.1021/ct3000734

[pro70543-bib-0004] Artur M , de Saulo O , Aram D , Tigran A , Gregory RB , van den Henry B . Discovery of a cryptic pocket in the AI‐predicted structure of PPM1D phosphatase explains the binding site and potency of its allosteric inhibitors. bioRxiv 2023.2003.2022.533829. 2023. 10.3389/fmolb.2023.1171143 PMC10151774 37143823

[pro70543-bib-0005] Backe SJ , Heritz JA , Mollapour M . Hsp70 and Hsp90 post‐translational modifications and translating the chaperone code. Cell Stress Chaperones. 2025;30(6):100118. 40976416 10.1016/j.cstres.2025.100118 PMC12513286

[pro70543-bib-0006] Backe SJ , Sager RA , Woodford MR , Makedon AM , Mollapour M . Post‐translational modifications of Hsp90 and translating the chaperone code. J Biol Chem. 2020;295(32):11099–11117. 32527727 10.1074/jbc.REV120.011833 PMC7415980

[pro70543-bib-0007] Baischew A , Engel S , Taubert MC , Geiger TM , Hausch F . Large‐scale, in‐cell photocrosslinking at single‐residue resolution reveals the molecular basis for glucocorticoid receptor regulation by immunophilins. Nat Struct Mol Biol. 2023;30(12):1857–1866. 37945739 10.1038/s41594-023-01098-1

[pro70543-bib-0008] Balega B , Beer M , Spencer J , Colombo G , Serapian SA , Oliveira ASF , et al. Dynamical nonequilibrium molecular dynamics simulations reveal allosteric networks, signal transduction mechanisms, and sites associated with drug resistance in biomolecular systems. Mol Phys. 2025;123(7–8):e2428350.

[pro70543-bib-0009] Bayly CI , Cieplak P , Cornell W , Kollman PA . A well‐behaved electrostatic potential based method using charge restraints for deriving atomic charges: the RESP model. J Phys Chem. 1993;97(40):10269–10280.

[pro70543-bib-0010] Berendsen HJC , Postma JPM , van Gunsteren WF , Di Nola A , Haak JR . Molecular dynamics with coupling to an external bath. J Chem Phys. 1984;81:3684–3690.

[pro70543-bib-0011] Bhattacharya K , Picard D . The Hsp70–Hsp90 go‐between Hop/Stip1/Sti1 is a proteostatic switch and may be a drug target in cancer and neurodegeneration. Cell Mol Life Sci. 2021;78(23):7257–7273. 34677645 10.1007/s00018-021-03962-z PMC8629791

[pro70543-bib-0012] Brehme M , Voisine C , Rolland T , Wachi S , Soper James H , Zhu Y , et al. A chaperome subnetwork safeguards proteostasis in aging and neurodegenerative disease. Cell Rep. 2014;9(3):1135–1150. 25437566 10.1016/j.celrep.2014.09.042 PMC4255334

[pro70543-bib-0013] Bunne C , Roohani Y , Rosen Y , Gupta A , Zhang X , Roed M , et al. How to build the virtual cell with artificial intelligence: priorities and opportunities. Cell. 2024;187(25):7045–7063. 39672099 10.1016/j.cell.2024.11.015 PMC12148494

[pro70543-bib-0014] Castelli M , Magni A , Bonollo G , Pavoni S , Frigerio F , Oliveira ASF , et al. Molecular mechanisms of chaperone‐directed protein folding: insights from atomistic simulations. Protein Sci. 2024;33(3):e4880. 10.1002/pro.4880 PMC10895457 38145386

[pro70543-bib-0015] Castelli M , Marchetti F , Osuna S , F. Oliveira AS , Mulholland AJ , Serapian SA , et al. Decrypting allostery in membrane‐bound K‐Ras4B using complementary in silico approaches based on unbiased molecular dynamics simulations. J Am Chem Soc. 2024;146(1):901–919. 38116743 10.1021/jacs.3c11396 PMC10785808

[pro70543-bib-0016] Castelli M , Yan P , Rodina A , Digwal CS , Panchal P , Chiosis G , et al. How aberrant N‐glycosylation can alter protein functionality and ligand binding: an atomistic view. Structure. 2023;31(8):987–1004.e1008. 37343552 10.1016/j.str.2023.05.017 PMC10526633

[pro70543-bib-0017] Charmandari E , Kino T , Ichijo T , Jubiz W , Mejia L , Zachman K , et al. A novel point mutation in helix 11 of the ligand‐binding domain of the human glucocorticoid receptor gene causing generalized glucocorticoid resistance. J Clin Endocrinol Metab. 2007;92(10):3986–3990. 17635946 10.1210/jc.2006-2830

[pro70543-bib-0018] Charmandari E , Raji A , Kino T , Ichijo T , Tiulpakov A , Zachman K , et al. A novel point mutation in the ligand‐binding domain (LBD) of the human glucocorticoid receptor (hGR) causing generalized glucocorticoid resistance: the importance of the C terminus of hGR LBD in conferring Transactivational activity. J Clin Endocrinol Metab. 2005;90(6):3696–3705. 15769988 10.1210/jc.2004-1920

[pro70543-bib-0019] Chen Z , Shiozaki M , Haas KM , Skinner WM , Zhao S , Guo C , et al. De novo protein identification in mammalian sperm using in situ cryoelectron tomography and AlphaFold2 docking. Cell. 2023;186(23):5041–5053.e5019. 37865089 10.1016/j.cell.2023.09.017 PMC10842264

[pro70543-bib-0020] Chio CM , Wang F , Noddings CM , Agard DA . Hsp90‐dependent triage of the glucocorticoid receptor via the CHIP E3 ubiquitin ligase bioRxiv 2025.2005.2028.656676. 2025.

[pro70543-bib-0021] Chiosis G , Digwal CS , Trepel JB , Neckers L . Structural and functional complexity of HSP90 in cellular homeostasis and disease. Nat Rev Mol Cell Biol. 2023;24(11):797–815. 37524848 10.1038/s41580-023-00640-9 PMC10592246

[pro70543-bib-0022] Ciccotti G , Ferrario M . Non‐equilibrium by molecular dynamics: a dynamical approach. Mol Simul. 2016;42(16):1385–1400.

[pro70543-bib-0023] Ciechanover A , Kwon YT . Protein quality control by molecular chaperones in neurodegeneration. Front Neurosci. 2017;11:185. 28428740 10.3389/fnins.2017.00185 PMC5382173

[pro70543-bib-0024] Dahiya V , Rutz DA , Moessmer P , Mühlhofer M , Lawatscheck J , Rief M , et al. The switch from client holding to folding in the Hsp70/Hsp90 chaperone machineries is regulated by a direct interplay between co‐chaperones. Mol Cell. 2022;82(8):1543–1556.e1546. 35176233 10.1016/j.molcel.2022.01.016

[pro70543-bib-0025] Darden T , York D , Pedersen L . Particle mesh Ewald: an N‐log(N) method for Ewald sums in large systems. J Chem Phys. 1993;98:10089–10092.

[pro70543-bib-0026] Dollins DE , Warren JJ , Immormino RM , Gewirth DT . Structures of GRP94‐nucleotide complexes reveal mechanistic differences between the hsp90 chaperones. Mol Cell. 2007;28(1):41–56. 17936703 10.1016/j.molcel.2007.08.024 PMC2094010

[pro70543-bib-0027] Echeverria PC , Bernthaler A , Dupuis P , Mayer B , Picard D . An interaction network predicted from public data as a discovery tool: application to the Hsp90 molecular chaperone machine. PLoS One. 2012;6(10):e26044. 10.1371/journal.pone.0026044 PMC3195953 22022502

[pro70543-bib-0028] Ferguson A , Wang L , Altman Roger B , Terry DS , Juette MF , Burnett BJ , et al. Functional dynamics within the human ribosome regulate the rate of active protein synthesis. Mol Cell. 2015;60(3):475–486. 26593721 10.1016/j.molcel.2015.09.013 PMC4660248

[pro70543-bib-0029] Finka A , Goloubinoff P . Proteomic data from human cell cultures refine mechanisms of chaperone‐mediated protein homeostasis. Cell Stress Chaperones. 2013;18:591–605. 23430704 10.1007/s12192-013-0413-3 PMC3745260

[pro70543-bib-0030] Frisch MJ , Trucks GW , Schlegel HB , Scuseria GE , Robb MA , Cheeseman JR , et al. Gaussian 16 Rev. C.02. Wallingford, CT; 2016.

[pro70543-bib-0031] Gershenson A , Gierasch LM . Protein folding in the cell: challenges and progress. Curr Opin Struct Biol. 2011;21(1):32–41. 21112769 10.1016/j.sbi.2010.11.001 PMC3072030

[pro70543-bib-0032] Gestwicki JE . Multi‐protein complexes as drug targets. Cell Chem Biol. 2022;29(5):713–715. 35594848 10.1016/j.chembiol.2022.05.002

[pro70543-bib-0033] Goldberg AP , Szigeti B , Chew YH , Sekar JAP , Roth YD , Karr JR . Emerging whole‐cell modeling principles and methods. Curr Opin Biotechnol. 2018;51:97–102. 29275251 10.1016/j.copbio.2017.12.013 PMC5997489

[pro70543-bib-0034] Guarra F , Sciva C , Bonollo G , Pasala C , Chiosis G , Moroni E , et al. Cracking the chaperone code through the computational microscope. Cell Stress Chaperones. 2024;29(5):626–640. 39142378 10.1016/j.cstres.2024.08.001 PMC11399801

[pro70543-bib-0035] Hadley KE , Louw A , Hapgood JP . Differential nuclear localisation and promoter occupancy play a role in glucocorticoid receptor ligand‐specific transcriptional responses. Steroids. 2011;76(10):1176–1184. 21641918 10.1016/j.steroids.2011.05.007

[pro70543-bib-0036] Hartl FU . Molecular chaperones in cellular protein folding. Nature. 1996;381(6583):571–580. 8637592 10.1038/381571a0

[pro70543-bib-0037] Hartl FU , Bracher A , Hayer‐Hartl M . Molecular chaperones in protein folding and proteostasis. Nature. 2011;475:324–332. 21776078 10.1038/nature10317

[pro70543-bib-0038] Hua G , Paulen L , Chambon P . GR SUMOylation and formation of an SUMO‐SMRT/NCoR1‐HDAC3 repressing complex is mandatory for GC‐induced IR nGRE‐mediated transrepression. Proc Natl Acad Sci U S A. 2016;113(5):E626–E634. 26712002 10.1073/pnas.1522821113 PMC4747746

[pro70543-bib-0039] Johnson OT , Gestwicki JE . Multivalent protein–protein interactions are pivotal regulators of eukaryotic Hsp70 complexes. Cell Stress Chaperones. 2022;27(4):397–415. 35670950 10.1007/s12192-022-01281-1 PMC9346034

[pro70543-bib-0040] Johnston RC , Yao K , Kaplan Z , Chelliah M , Leswing K , Seekins S , et al. Epik: pKa and protonation state prediction through machine learning. J Chem Theory Comput. 2023;19(8):2380–2388. 37023332 10.1021/acs.jctc.3c00044

[pro70543-bib-0041] Jorgensen WL , Chandrasekhar J , Madura J , Impey RW , Klein ML . Comparison of simple potential functions for simulating liquid water. J Chem Phys. 1983;79:926–935.

[pro70543-bib-0042] Joung IS , Cheatham TE . Determination of alkali and halide monovalent ion parameters for use in explicitly solvated biomolecular simulations. J Phys Chem B. 2008;112(30):9020–9041. 18593145 10.1021/jp8001614 PMC2652252

[pro70543-bib-0043] Kadmiel M , Cidlowski JA . Glucocorticoid receptor signaling in health and disease. Trends Pharmacol Sci. 2013;34(9):518–530. 23953592 10.1016/j.tips.2013.07.003 PMC3951203

[pro70543-bib-0044] Kashefolgheta S , Vila Verde A . Developing force fields when experimental data is sparse: AMBER/GAFF‐compatible parameters for inorganic and alkyl oxoanions. Phys Chem Chem Phys. 2017;19(31):20593–20607. 28731091 10.1039/c7cp02557b

[pro70543-bib-0045] Kim YE , Hipp MS , Bracher A , Hayer‐Hartl M , Hartl FU . Molecular chaperone functions in protein folding and Proteostasis. Annu Rev Biochem. 2013;2013(82):323–355. 10.1146/annurev-biochem-060208-092442 23746257

[pro70543-bib-0046] Kino T , Stauber RH , Resau JH , Pavlakis GN , Chrousos GP . Pathologic human GR mutant has a Transdominant negative effect on the wild‐type GR by inhibiting its translocation into the nucleus: importance of the ligand‐binding domain for intracellular GR trafficking. J Clin Endocrinol Metab. 2001;86(11):5600–5608. 11701741 10.1210/jcem.86.11.8017

[pro70543-bib-0047] Kirschke E , Goswami D , Southworth D , Griffin PR , Agard DA . Glucocorticoid receptor function regulated by coordinated action of the Hsp90 and Hsp70 chaperone cycles. Cell. 2014;157(7):1685–1697. 24949977 10.1016/j.cell.2014.04.038 PMC4087167

[pro70543-bib-0048] Kolhe JA , Babu NL , Freeman BC . The Hsp90 molecular chaperone governs client proteins by targeting intrinsically disordered regions. Mol Cell. 2023;83(12):2035–2044.e2037. 37295430 10.1016/j.molcel.2023.05.021 PMC10297700

[pro70543-bib-0049] Kurop MK , Huyen CM , Kelly JH , Blagg BSJ . The heat shock response and small molecule regulators. Eur J Med Chem. 2021;226:113846. 34563965 10.1016/j.ejmech.2021.113846 PMC8608735

[pro70543-bib-0050] Laine E , Carbone A . Protein social behavior makes a stronger signal for partner identification than surface geometry. Proteins Struct Funct Bioinform. 2017;85(1):137–154. 10.1002/prot.25206 PMC5242317 27802579

[pro70543-bib-0051] Lavery LA , Partridge JR , Ramelot TA , Elnatan D , Kennedy MA , Agard DA . Structural asimmetry in the closed state of mitochondrial Hsp90 (TRAP1) supports a two‐step ATP hydrolysis mechanism. Mol Cell. 2014;53(2):330–343. 24462206 10.1016/j.molcel.2013.12.023 PMC3947485

[pro70543-bib-0052] Lee C , Yang W , Parr RG . Development of the Colle‐Salvetti correlation‐energy formula into a functional of the electron density. Phys Rev B. 1988;37(2):785–789. 10.1103/physrevb.37.785 9944570

[pro70543-bib-0053] Li J , Richter K , Reinstein J , Buchner J . Integration of the accelerator Aha1 in the Hsp90 co‐chaperone cycle. Nat Struct Mol Biol. 2013;20:326–331. 23396352 10.1038/nsmb.2502

[pro70543-bib-0054] Lim CM , Vendruscolo M . Proteostasis signatures in human diseases. PLoS Comput Biol. 2025;21(6):e1013155. 40526761 10.1371/journal.pcbi.1013155 PMC12173376

[pro70543-bib-0055] Loncharich RJ , Brooks BR , Pastor RW . Langevin dynamics of peptides: the frictional dependence of isomerization rates of N‐acetylalanyl‐N′‐methylamide. Biopolymers. 1992;32(5):523–535. 1515543 10.1002/bip.360320508

[pro70543-bib-0056] Lopez A , Dahiya V , Delhommel F , Freiburger L , Stehle R , Asami S , et al. Client binding shifts the populations of dynamic Hsp90 conformations through an allosteric network. Sci Adv. 2021;7(51):eabl7295. 34919431 10.1126/sciadv.abl7295 PMC8682993

[pro70543-bib-0057] Lorenz OR , Freiburger L , Rutz DA , Krause M , Zierer BK , Alvira S , et al. Modulation of the Hsp90 chaperone cycle by a stringent client protein. Mol Cell. 2014;53(6):941–953. 24613341 10.1016/j.molcel.2014.02.003

[pro70543-bib-0058] Mader SL , Lopez A , Lawatscheck J , Luo Q , Rutz DA , Gamiz‐Hernandez AP , et al. Conformational dynamics modulate the catalytic activity of the molecular chaperone Hsp90. Nat Commun. 2020;11(1):1410. 32179743 10.1038/s41467-020-15050-0 PMC7075974

[pro70543-bib-0059] Maier JA , Martinez C , Kasavajhala K , Wickstrom L , Hauser KE , Simmerling C . ff14SB: improving the accuracy of protein side chain and backbone parameters from ff99SB. J Chem Theory Comput. 2015;11(8):3696–3713. 26574453 10.1021/acs.jctc.5b00255 PMC4821407

[pro70543-bib-0060] Meagher KL , Redman LT , Carlson HA . Development of polyphosphate parameters for use with the AMBER force field. J Comput Chem. 2003;24(9):1016–1025. 12759902 10.1002/jcc.10262

[pro70543-bib-0061] Mecha MF , Hutchinson RB , Lee JH , Cavagnero S . Protein folding in vitro and in the cell: from a solitary journey to a team effort. Biophys Chem. 2022;287:106821. 35667131 10.1016/j.bpc.2022.106821 PMC9636488

[pro70543-bib-0062] Meli M , Morra G , Colombo G . Simple model of protein energetics to identify ab initio folding transitions from all‐atom MD simulations of proteins. J Chem Theory Comput. 2020;16(9):5960–5971. 32693598 10.1021/acs.jctc.0c00524 PMC8009504

[pro70543-bib-0063] Miles J , Scherz‐Shouval R , van Oosten‐Hawle P . Expanding the organismal Proteostasis network: linking systemic stress signaling with the innate immune response. Trends Biochem Sci. 2019;44:927–942. 31303384 10.1016/j.tibs.2019.06.009

[pro70543-bib-0064] Miyamoto S , Kollman PA . SETTLE: an analytical version of the SHAKE and RATTLE algorithms for rigid water models. J Comput Chem. 1992;13:952–962.

[pro70543-bib-0065] Moroni E , Agard DA , Colombo G . The structural asymmetry of mitochondrial Hsp90 (Trap1) determines fine tuning of functional dynamics. J Chem Theory Comput. 2018;14(2):1033–1044. 29320629 10.1021/acs.jctc.7b00766

[pro70543-bib-0066] Morra G , Potestio R , Micheletti C , Colombo G . Corresponding functional dynamics across the Hsp90 chaperone family: insights from a multiscale analysis of MD simulations. PLoS Comput Biol. 2012;8(3):e1002433. 22457611 10.1371/journal.pcbi.1002433 PMC3310708

[pro70543-bib-0067] Nicolaides NC , Skyrla E , Vlachakis D , Psarra A‐MG , Moutsatsou P , Sertedaki A , et al. Functional characterization of the hGRαT556I causing Chrousos syndrome. Eur J Clin Invest. 2016;46(1):42–49. 26541474 10.1111/eci.12563

[pro70543-bib-0068] Noddings CM , Johnson JL , Agard DA . Cryo‐EM reveals how Hsp90 and FKBP immunophilins co‐regulate the glucocorticoid receptor. Nat Struct Mol Biol. 2023;30:1867–1877. 37945740 10.1038/s41594-023-01128-y PMC10716051

[pro70543-bib-0069] Noddings CM , Wang RY‐R , Johnson JL , Agard DA . Structure of Hsp90–p23–GR reveals the Hsp90 client‐remodelling mechanism. Nature. 2022;601(7893):465–469. 34937936 10.1038/s41586-021-04236-1 PMC8994517

[pro70543-bib-0070] Nussinov R , Tsai C‐J , Jang H . Protein ensembles link genotype to phenotype. PLoS Comput Biol. 2019;15(6):e1006648‐e1006648. 31220071 10.1371/journal.pcbi.1006648 PMC6586255

[pro70543-bib-0071] Nussinov R , Tsai C‐J , Jang H . Signaling in the crowded cell. Curr Opin Struct Biol. 2021;71:43–50. 34218161 10.1016/j.sbi.2021.05.009 PMC8648894

[pro70543-bib-0072] Oakley RH , Cidlowski JA . Cellular processing of the glucocorticoid receptor gene and Protein: new mechanisms for generating tissue‐specific actions of glucocorticoids *. J Biol Chem. 2011;286(5):3177–3184. 21149445 10.1074/jbc.R110.179325 PMC3030321

[pro70543-bib-0073] Oliveira ASF , Ciccotti G , Haider S , Mulholland AJ . Dynamical nonequilibrium molecular dynamics reveals the structural basis for allostery and signal propagation in biomolecular systems. The European Physical Journal B. 2021;94(7):144. 34720710 10.1140/epjb/s10051-021-00157-0 PMC8549953

[pro70543-bib-0074] Oliveira ASF , Edsall CJ , Woods CJ , Bates P , Nunez GV , Wonnacott S , et al. A general mechanism for signal propagation in the nicotinic acetylcholine receptor family. J Am Chem Soc. 2019;141(51):19953–19958. 31805762 10.1021/jacs.9b09055

[pro70543-bib-0075] Oliveira ASF , Shoemark DK , Avila Ibarra A , Davidson AD , Berger I , Schaffitzel C , et al. The fatty acid site is coupled to functional motifs in the SARS‐CoV‐2 spike protein and modulates spike allosteric behaviour. Comput Struct Biotechnol J. 2022;20:139–147. 34934478 10.1016/j.csbj.2021.12.011 PMC8670790

[pro70543-bib-0076] Oliveira ASF , Shoemark DK , Campello HR , Wonnacott S , Gallagher T , Sessions RB , et al. Identification of the initial steps in signal transduction in the α4β2 nicotinic receptor: insights from equilibrium and nonequilibrium simulations. Structure. 2019;27(7):1171–1183.e1173. 31130483 10.1016/j.str.2019.04.008

[pro70543-bib-0077] Olsson MHM , Søndergaard CR , Rostkowski M , Jensen JH . PROPKA3: consistent treatment of internal and surface residues in empirical pKa predictions. J Chem Theory Comput. 2011;7(2):525–537. 26596171 10.1021/ct100578z

[pro70543-bib-0078] Pearl LH , Prodromou C . Structure and mechanism of the Hsp90 molecular chaperone machinery. Annu Rev Biochem. 2006;75:271–294. 16756493 10.1146/annurev.biochem.75.103004.142738

[pro70543-bib-0079] Pokorná P , Aupič J , Fica SM , Magistrato A . Decoding spliceosome dynamics through computation and experiment. Chem Rev. 2025;125:9807–9833. 41071962 10.1021/acs.chemrev.5c00374

[pro70543-bib-0080] Qu X , Wang S , Zhao S , Wan C , Xu W , Huang C . The dynamic triage interplay of Hsp90 with its chaperone cycle and client binding. Nat Commun. 2024;15(1):10661. 39663352 10.1038/s41467-024-55026-y PMC11634960

[pro70543-bib-0081] Rehn A , Lawatscheck J , Jokisch M‐L , Mader SL , Luo Q , Tippel F , et al. A methylated lysine is a switch point for conformational communication in the chaperone Hsp90. Nat Commun. 2020;11(1):1219. 32139682 10.1038/s41467-020-15048-8 PMC7057950

[pro70543-bib-0082] Rodina A , Xu C , Digwal CS , Joshi S , Patel Y , Santhaseela AR , et al. Systems‐level analyses of protein‐protein interaction network dysfunctions via epichaperomics identify cancer‐specific mechanisms of stress adaptation. Nat Commun. 2023;14(1):3742. 37353488 10.1038/s41467-023-39241-7 PMC10290137

[pro70543-bib-0083] Roe DR , Cheatham TE III . Parallelization of CPPTRAJ enables large scale analysis of molecular dynamics trajectory data. J Comput Chem. 2018;39(25):2110–2117. 30368859 10.1002/jcc.25382 PMC7313716

[pro70543-bib-0084] Sabbagh JJ , Cordova RA , Zheng D , Criado‐Marrero M , Lemus A , Li P , et al. Targeting the FKBP51/GR/Hsp90 complex to identify functionally relevant treatments for depression and PTSD. ACS Chem Biol. 2018;13(8):2288–2299. 29893552 10.1021/acschembio.8b00454 PMC6126901

[pro70543-bib-0085] Sahasrabudhe P , Rohrberg J , Biebl MM , Rutz DA , Buchner J . The plasticity of the Hsp90 Co‐chaperone system. Mol Cell. 2017;67(6):947–961.e945. 28890336 10.1016/j.molcel.2017.08.004

[pro70543-bib-0086] Sali A . From integrative structural biology to cell biology. J Biol Chem. 2021;296:100743. 33957123 10.1016/j.jbc.2021.100743 PMC8203844

[pro70543-bib-0087] Schmid S , Hugel T . Controlling protein function by fine‐tuning conformational flexibility. Elife. 2020;9:e57180. 32697684 10.7554/eLife.57180 PMC7375816

[pro70543-bib-0088] Schopf FH , Biebl MM , Buchner J . The HSP90 chaperone machinery. Nat Rev Mol Cell Biol. 2017;18(6):345–360. 28429788 10.1038/nrm.2017.20

[pro70543-bib-0089] Serapian SA , Marchetti F , Triveri A , Morra G , Meli M , Moroni E , et al. The answer lies in the energy: how simple atomistic molecular dynamics simulations may hold the key to epitope prediction on the fully glycosylated SARS‐CoV‐2 spike protein. J Phys Chem Lett. 2020;11:8084–8093. 32885971 10.1021/acs.jpclett.0c02341 PMC7491317

[pro70543-bib-0090] Serapian SA , Moroni E , Ferraro M , Colombo G . Atomistic simulations of the mechanisms of the poorly catalytic mitochondrial chaperone Trap1: insights into the effects of structural asymmetry on reactivity. ACS Catal. 2021;11(14):8605–8620.

[pro70543-bib-0091] Sherman W , Day T , Jacobson MP , Friesner RA , Farid R . Novel procedure for modeling ligand/receptor induced fit effects. J Med Chem. 2006;49(2):534–553. 16420040 10.1021/jm050540c

[pro70543-bib-0092] Shiau AK , Harris SF , Southworth DR , Agard DA . Structural analysis of E‐coli hsp90 reveals dramatic nucleotide‐dependent conformational rearrangements. Cell. 2006;127(2):329–340. 17055434 10.1016/j.cell.2006.09.027

[pro70543-bib-0093] Shimojo M , Hiroi N , Yakushiji F , Ueshiba H , Yamaguchi N , Miyachi Y . Differences in Down‐regulation of glucocorticoid receptor mRNA by cortisol prednisolone and dexamethasone in HeLa cells. Endocr J. 1995;42(5):629–636. 8574285 10.1507/endocrj.42.629

[pro70543-bib-0094] Silbermann L‐M , Vermeer B , Schmid S , Tych K . The known unknowns of the Hsp90 chaperone. Elife. 2024;13:e102666. 39737863 10.7554/eLife.102666 PMC11687934

[pro70543-bib-0095] Sohmen B , Beck C , Frank V , Seydel T , Hoffmann I , Hermann B , et al. The onset of molecule‐spanning dynamics in heat shock protein Hsp90. Adv Sci. 2023;10(36):2304262. 10.1002/advs.202304262 PMC10754087 37984887

[pro70543-bib-0096] Tanaka K . The proteasome: overview of structure and functions. Proceedings of the Japan Academy, Series B. 2009;85(1):12–36. 10.2183/pjab.85.12 PMC3524306 19145068

[pro70543-bib-0097] Truman AW , Bourboulia D , Mollapour M . Decrypting the chaperone code. J Biol Chem. 2021;296:100293. 33837727 10.1016/j.jbc.2021.100293 PMC7949055

[pro70543-bib-0098] Vicent GP , Pecci A , Ghini A , Piwien‐Pilipuk G , Galigniana MD . Differences in nuclear retention characteristics of agonist‐activated glucocorticoid receptor may determine specific responses. Exp Cell Res. 2002;276(2):142–154. 12027445 10.1006/excr.2002.5532

[pro70543-bib-0099] Wang J , Wolf RM , Caldwell JW , Kollman PA , Case DA . Development and testing of a general amber force field. J Comput Chem. 2004;25(9):1157–1174. 15116359 10.1002/jcc.20035

[pro70543-bib-0100] Wang RY‐R , Noddings CM , Kirschke E , Myasnikov AG , Johnson JL , Agard DA . Structure of Hsp90–Hsp70–hop–GR reveals the Hsp90 client‐loading mechanism. Nature. 2022;601(7893):460–464. 34937942 10.1038/s41586-021-04252-1 PMC9179170

[pro70543-bib-0101] Wodak SJ , Paci E , Dokholyan NV , Berezovsky IN , Horovitz A , Li J , et al. Allostery in its many disguises: from theory to applications. Structure (London, England: 1993). 2019;27(4):566–578. 30744993 10.1016/j.str.2019.01.003 PMC6688844

[pro70543-bib-0102] Wolf S , Sohmen B , Hellenkamp B , Thurn J , Stock G , Hugel T . Allosteric action of nucleotides on Hsp90 across several time‐ and length scales. bioRxiv 2020.2002.2015.950725. 2020.

[pro70543-bib-0103] Zhou L , Roth M , Papakonstantinou E , Tamm M , Stolz D . Expression of glucocorticoid receptor and HDACs in airway smooth muscle cells is associated with response to steroids in COPD. Respir Res. 2024;25(1):227. 38812021 10.1186/s12931-024-02769-3 PMC11137987

