# Supplementary material for: Ligand‐driven modulation of chaperone–cochaperone networks shapes proteostasis outcomes
Source: Protein Sci. 2026 Apr 8;35(5):e70543. doi: 10.1002/pro.70543 (PMC13059100; doi:10.1002/pro.70543)
Supplement: Supplementary file 1 — Data S1: Supporting Information. [file PRO-35-e70543-s001.pdf]

TABLE S1

Proteins	Domains	Selection
Hsp90A	NTD_A	[M30;D66] - [K74;G114] - [G132;A166] - [G168;T195]
	MiD_{1A}	[W297] - [R299;E332] - [Q334;D350] - [K358;N397] - [R400;L403] - [Q405;E422]
	MiD_{2A}	[S442;E537]
	CTD_A	[E547;L549] - [L551] - [E553;S602] - [K632;L671]
Hsp90B	NTD_B	[M30;P67] - [E75;I110] - [G114;E120] - [D127;A166] - [G168;T195]
	MiD_{1B}	[W297;P301] - [D303;E332] - [Q334;F349] - [K357;E432]
	MiD_{2B}	[S442;E537]
	CTD_B	[E547;L549] - [L551;P552] - [D554;S602] - [K632;L672]
GR	LBD	[R558;E631] - [L636;S765]
Hsp70C	NBD	[A4;R76] - [W90;R187] - [G191;G224] - [T226;S254] - [N256;I379]
	SBD	[D395;T502]
Hsp70S	NBD	[A4;I379]
Hop	Hop_1	[D217;E329]
	Hop_2	[L359;Q469]
	Hop_3	[D484] - [K486;L539]
p23	Core	[A4;D102]
	Tail α-helix	[M117;G131]
FKBP51	FK_1	[G32;V37] - [K52;D68] - [H71;R73] - [E75;F79] - [I87;K99] - [E101;K108] - [A126;L134]
	FK_2	[G147;N163] - [G165;R175] - [D182;G220] - [L241;E251]
	TPR_1	[T261;L346]
	TPR_2	[L362;K397]
FKBP52	FK_1	[G50;S69] - [L71;E110] - [N125;K138]
	FK_2	[I150;A226] - [A241;K254]
	TPR_1	[E264;E303] - [F306;D349]
	TPR_2	[G355;I400]

Table S1: A summary of the TOG domain division (final selection) for each protein belonging to the GR activation cycle multiprotein assemblies.

FIGURE S1

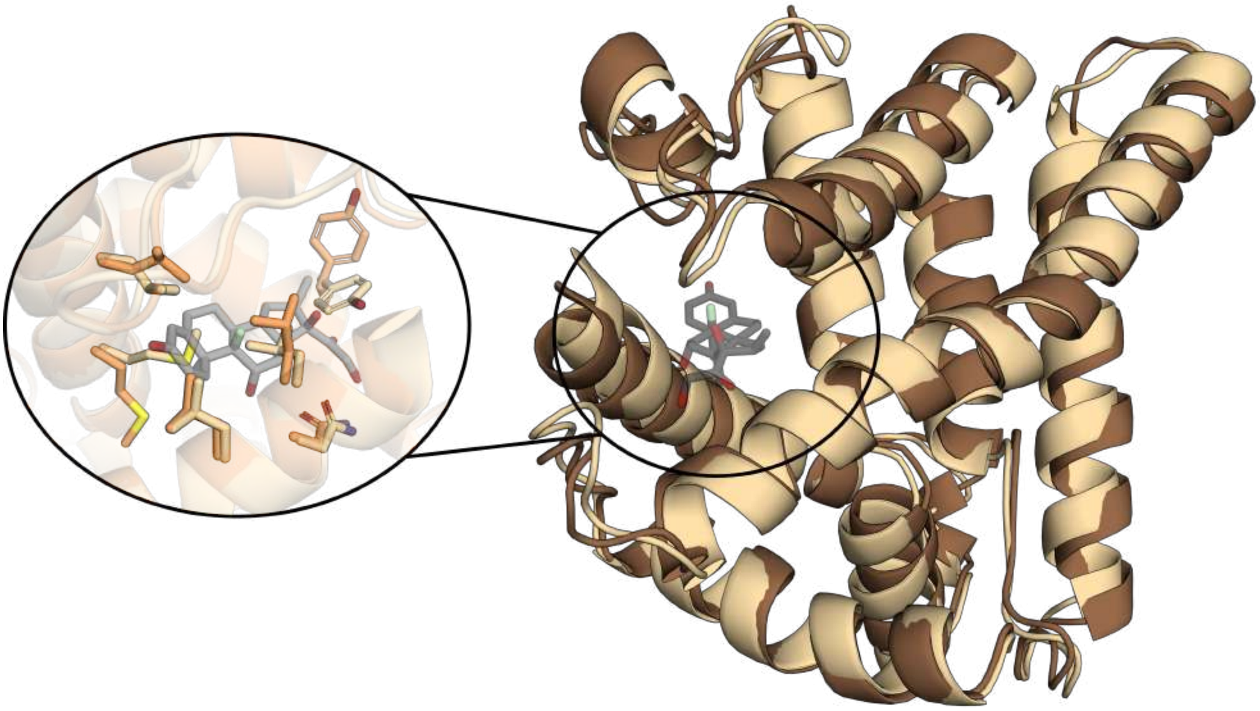

Figure S1: Synthetic agonist DEX (dexamethasone) docked into the GR active site. On the right, we superimpose the GR core backbone from frames isolated from MD simulations of the Loading-DEX complex (light yellow) and Maturation+DEX complex sharing the lowest RMSD pair. On the left, a zoomed view of the DEX docking pose (grey sticks) in the GR active site: light yellow sticks represent the residue side chains before trimming; while dark yellow sticks represent their new post-docking positions, highlighting the active site remodeling.

FIGURE S2

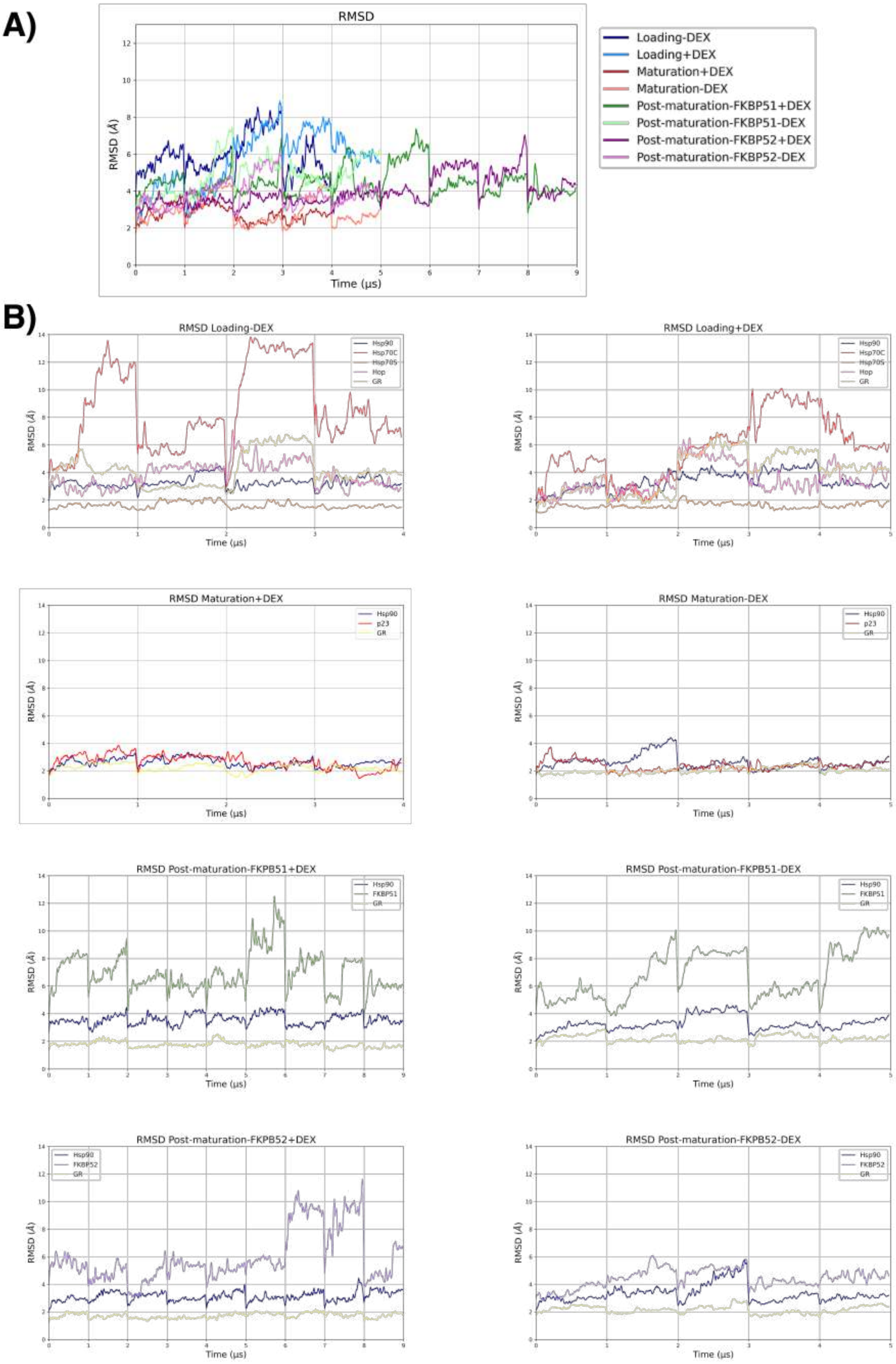

Figure S2: RMSD plots of **(A)** the Cαs of the secondary structures along each simulation and **(B)** the Cαs of each single protein component.

FIGURE S3

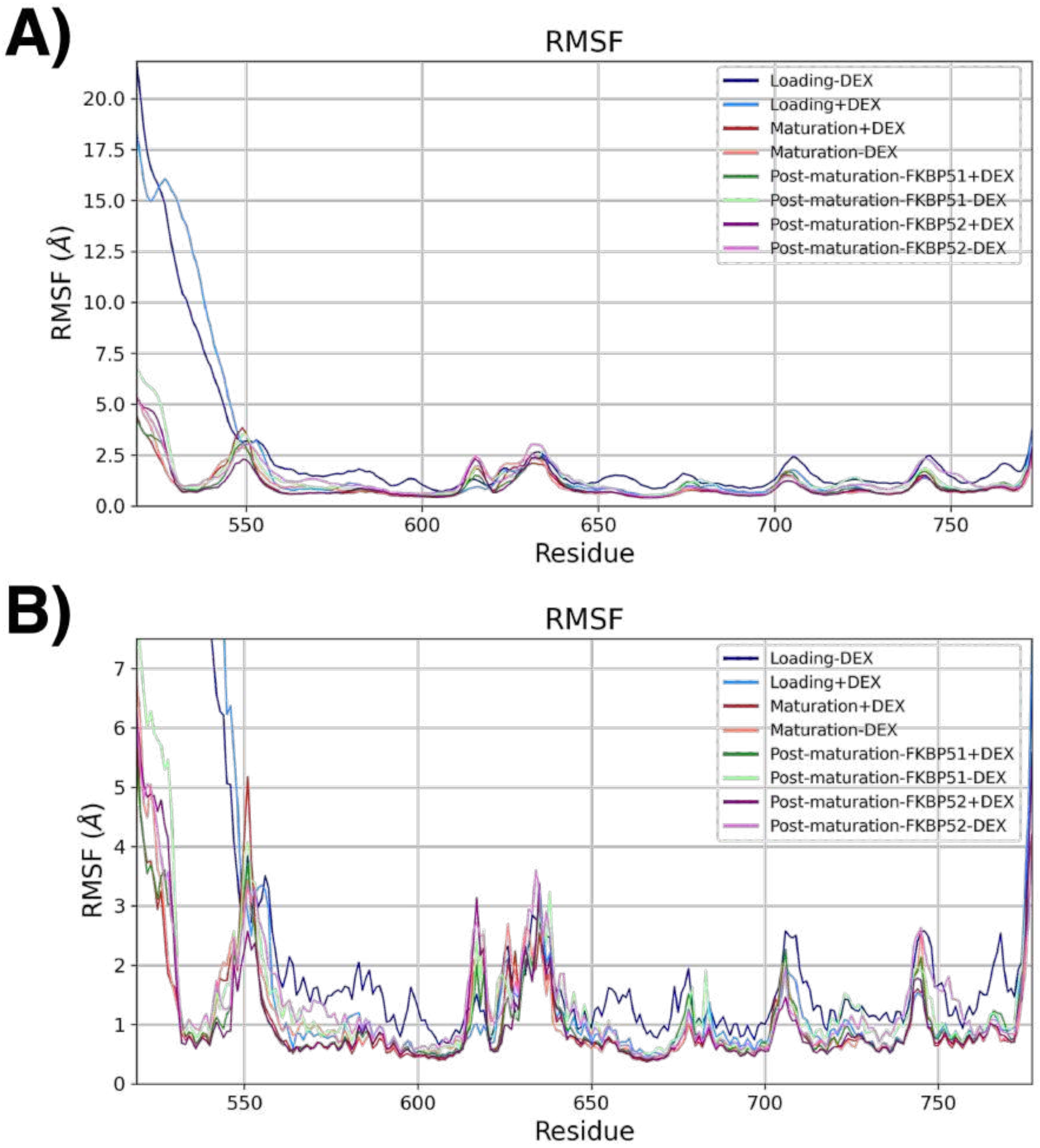

Figure S3: GR RMSF plot for all equilibrium simulations (equilibrium systems are in darker colors; perturbed systems in lighter colors). For better visualization, the plot lines are raw data convoluted over blocks of 5 residues. **(A)** Raw RMSF result. **(B)** A zoomed view in the 0-7.25 Å RMSF range.

FIGURE S4

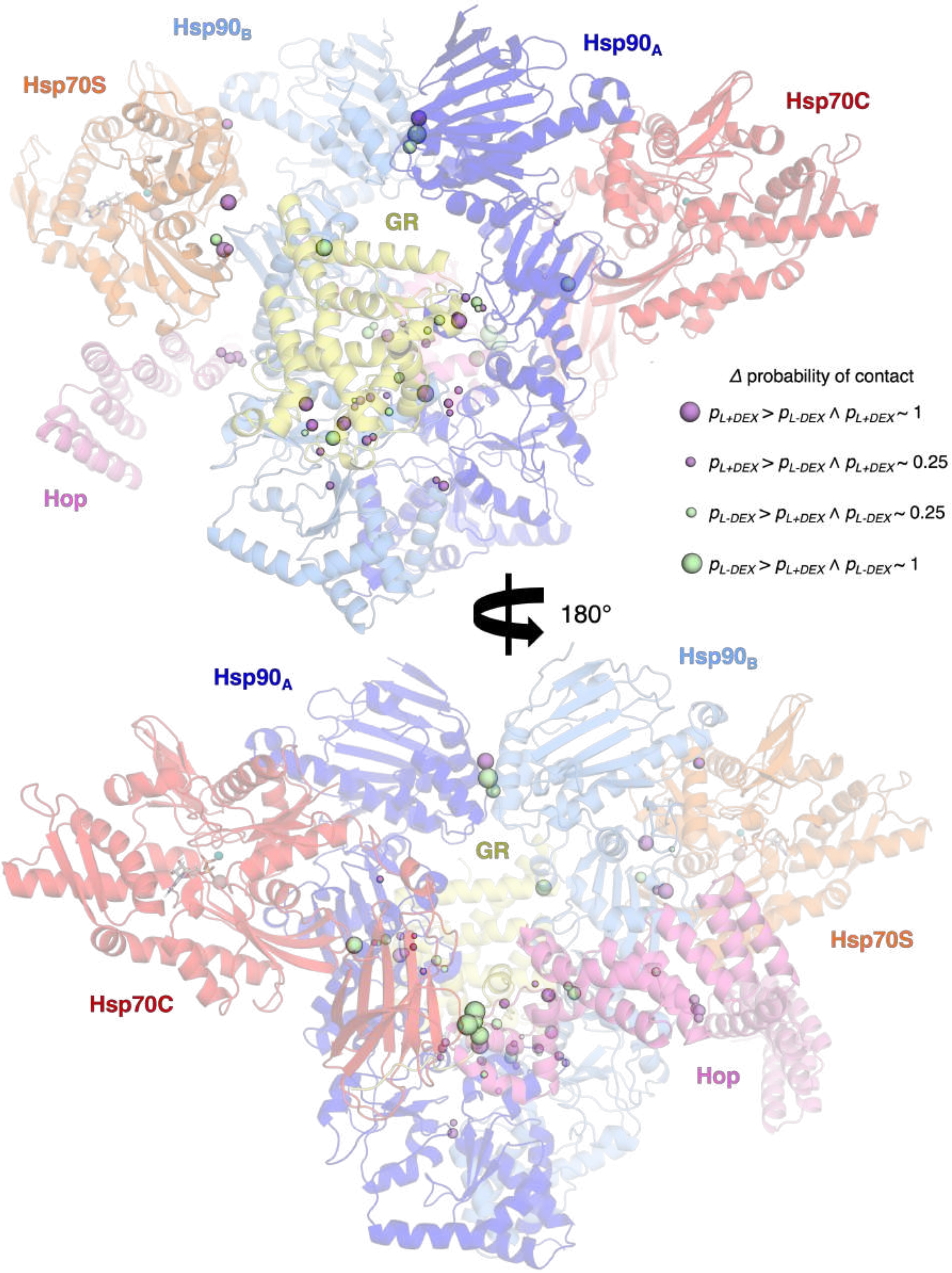

Figure S4: Projection of differences in probability of contact onto the Loading complex structure (the front view on the top; the rear view on the bottom). Each sphere indicates the presence of a statistically significant modified interaction between each inter-protein residue pair contained in the unperturbed and perturbed contact matrices. Spheres mark the midpoint between the two Cα atoms involved in each interaction, identifying the approximate contact location. Contacts more likely in the Loading-DEX complex are shown in green; those more likely in Loading+DEX are in violet. Sphere radius reflects the maximum likelihood (probability) of the corresponding contact.

FIGURE S5

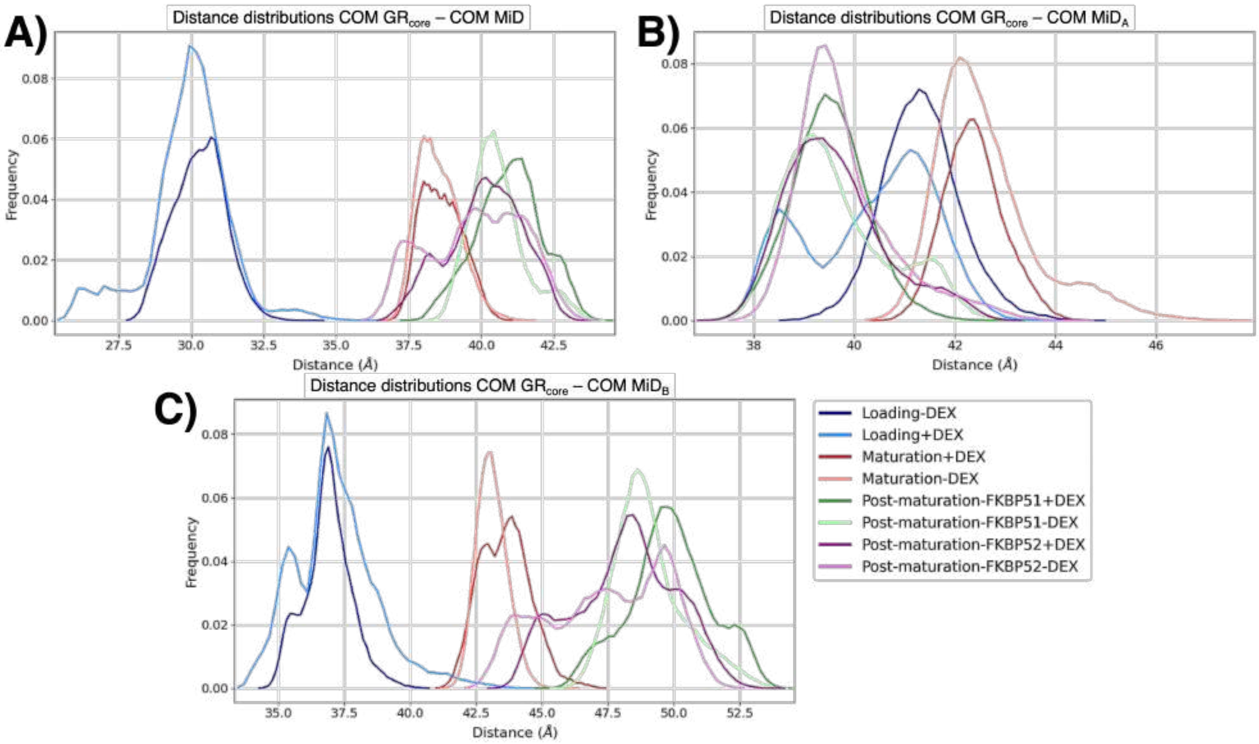

Figure S5: Distance distributions between the center of mass of the GR core (residues 561-777) and: **(A)** the center of mass of the entire MiD (residues 282-546; both protomers); **(B)** the center of mass of MiD_A; **(C)** the center of mass of MiD_B.

FIGURE S6

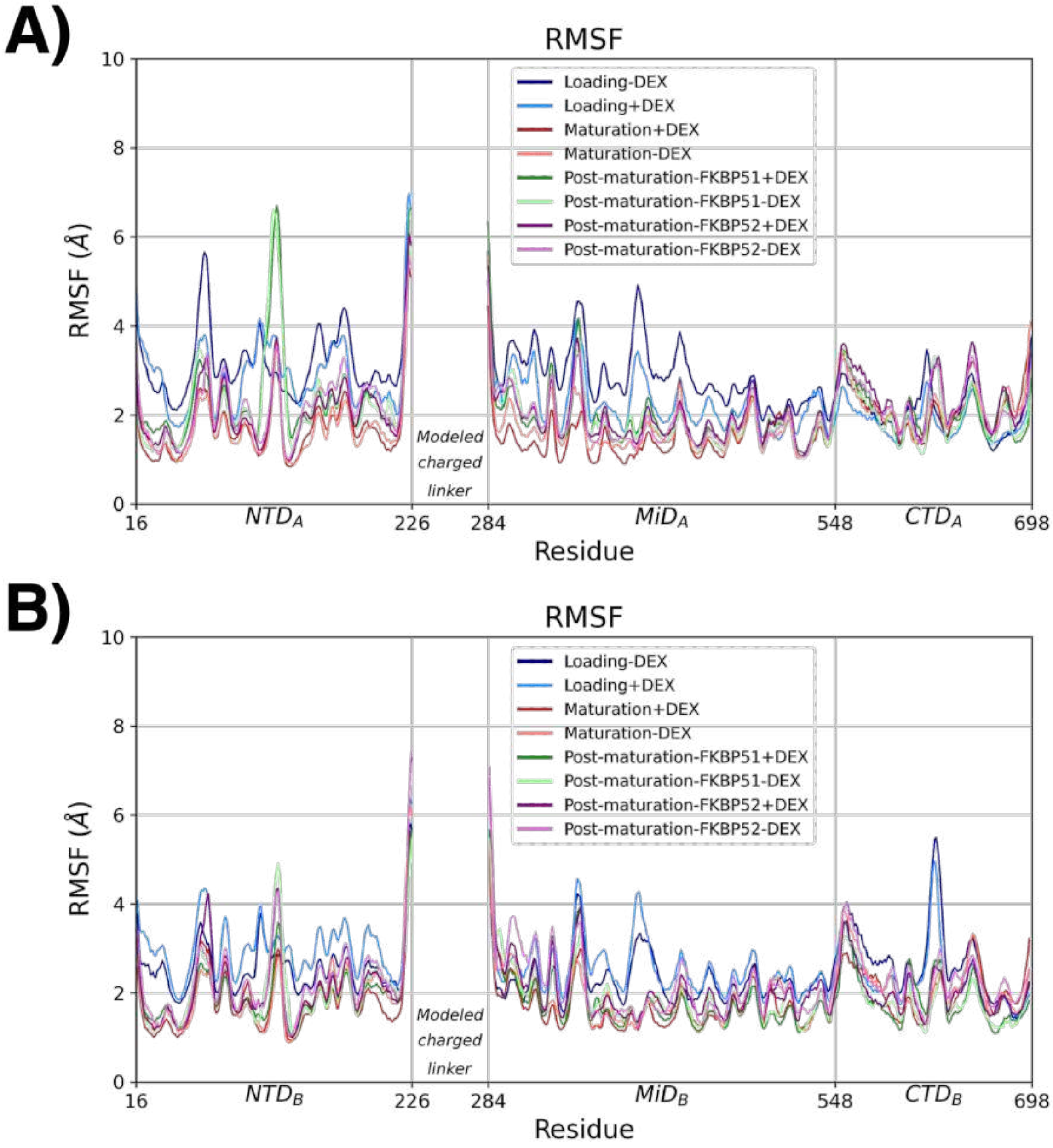

Figure S6: The RMSF of Hsp90 in equilibrium simulations of all complexes. Darker colors represent the WT simulations and lighter colors the perturbed ones. **(A)** Results for protomer A. **(B)** Results for protomer B.

FIGURE S7

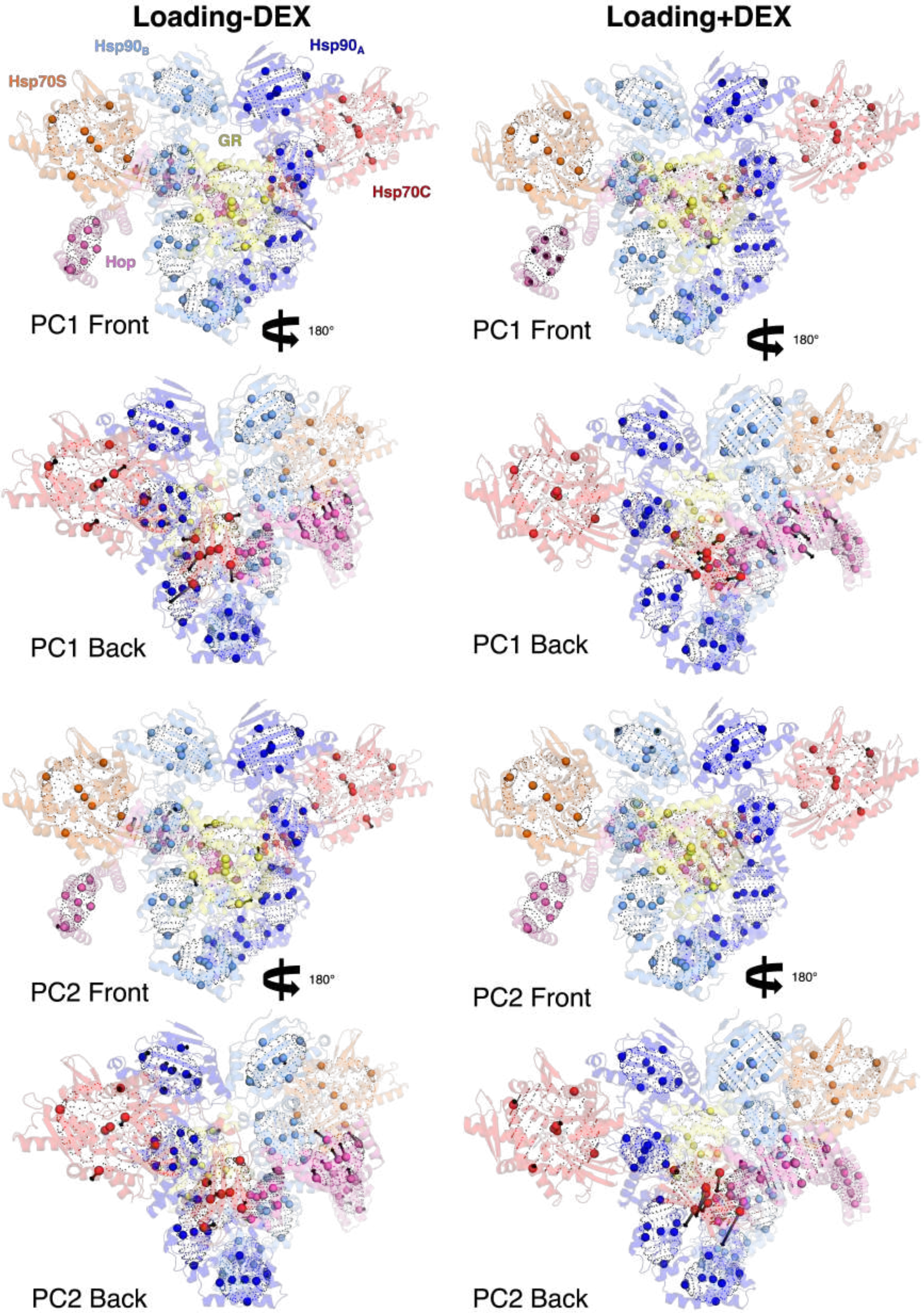

Figure S7: Projection of the first two PCA eigenvectors (vectors as arrows) onto structures of the Loading complex, from unperturbed (Loading-DEX; left) and perturbed simulations (Loading+DEX; right). Spheres represent the TOG-derived reduced points, while dots outline the shape of the identified ellipsoids that include each cluster of spheres. For each PC, the front and rear views are provided.

FIGURE S8

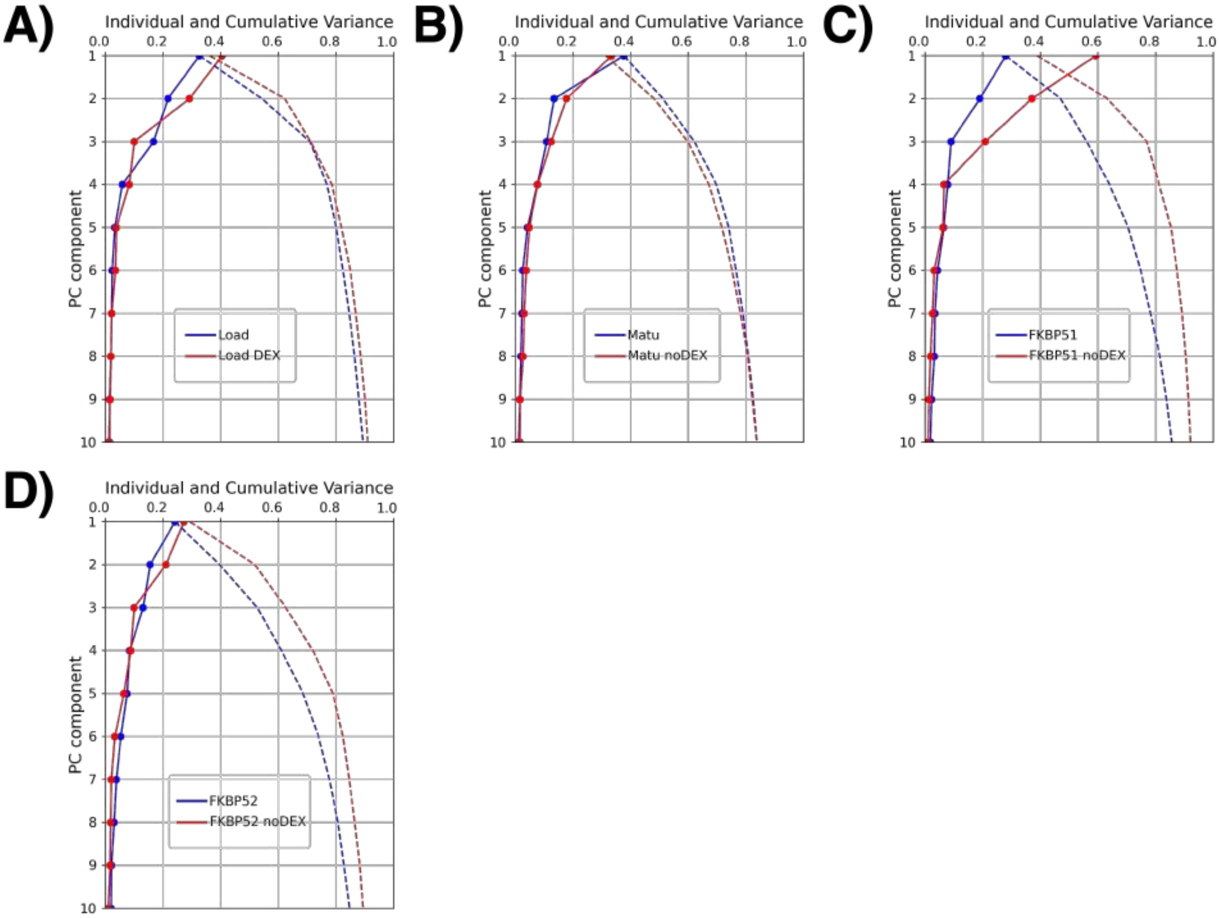

Figure S8: The plot of the first 10 principal components (PCs) from the TOG PCA eigenvalues (solid lines). Dashed lines represent the cumulative variance reached by each specific PC. The plots involve respectively: **(A)** the Loading-DEX complex and the perturbed Loading+DEX counterpart simulations; **(B)** the Maturation+DEX and Maturation-DEX; **(C)** Post-maturation-FKBP51+DEX and Post-maturation-FKBP51-DEX; **(D)** the Post-maturation-FKBP52+DEX and Post-maturation-FKBP52-DEX.

FIGURE S9

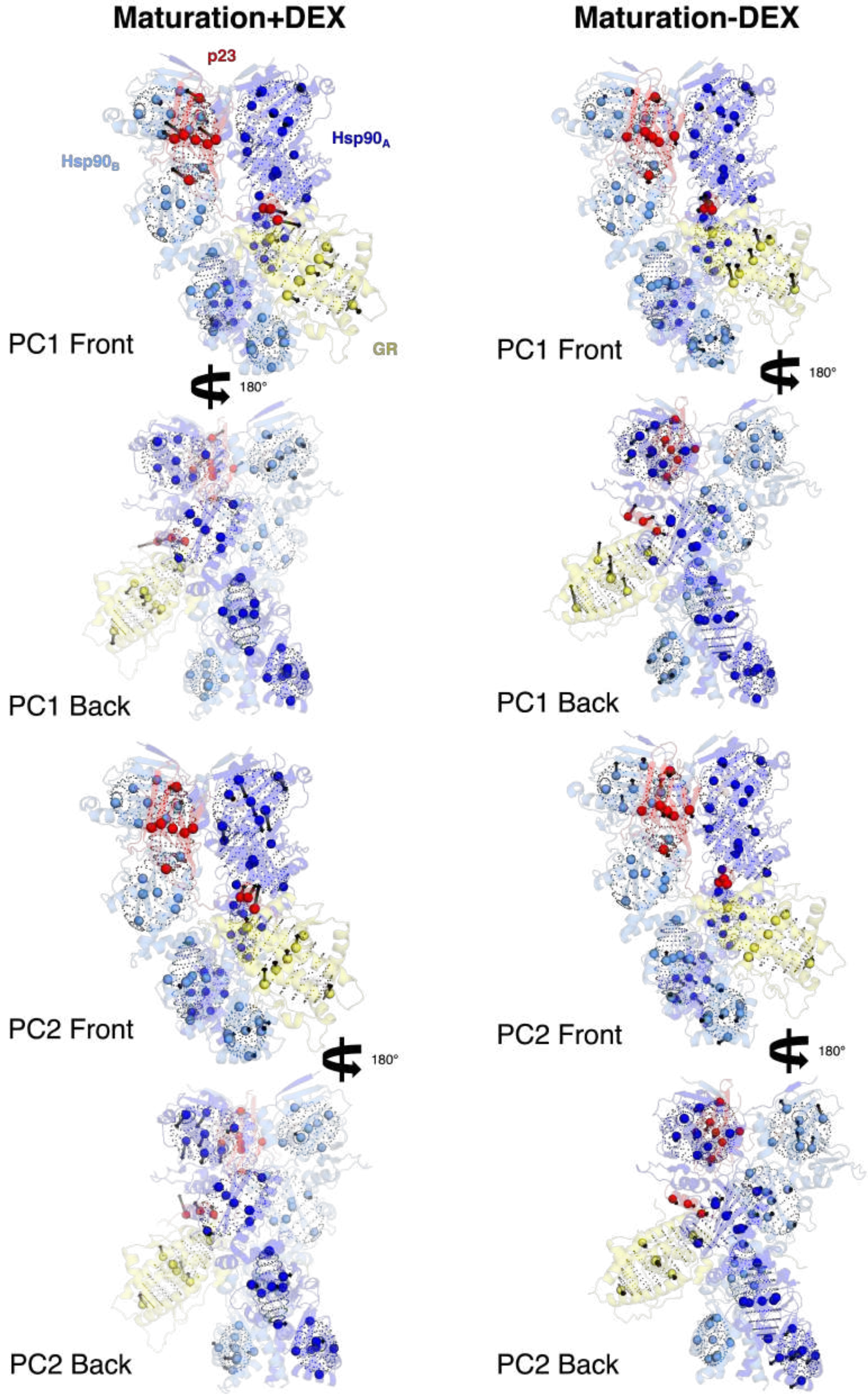

Figure S9: Projection of the first two PCA eigenvectors (vectors as arrows) onto structures of the Maturation complex, from unperturbed (Maturation+DEX; left) and perturbed simulations (Maturation-DEX; right). Spheres represent the TOG-derived reduced points, while dots outline the shape of the identified ellipsoids that include each cluster of spheres. For each PC, the front and rear views are provided.

FIGURE S10

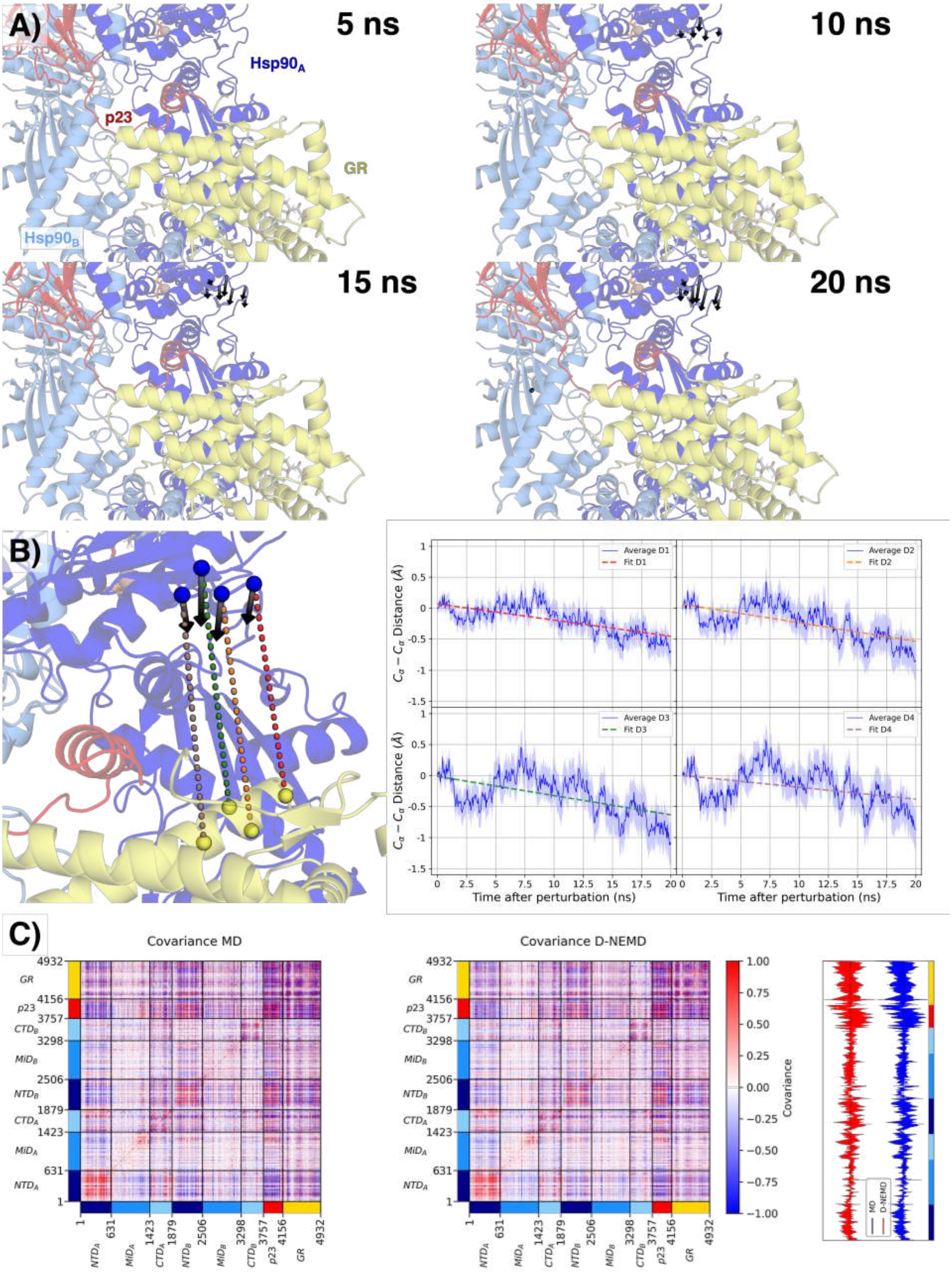

Figure S10: Internal D-NEMD simulation results for the Maturation+DEX complex upon dexamethasone removal. **(A)** The evolution over time of the displacement of four specific residues upon perturbation (magnified in panel **B**). Arrows indicate the average overall direction and

magnitude of the captured perturbed motion. **(B)** On the left, the four relevant residues whose motion is shown in **A** are represented in a similar fashion. Dotted lines connect each affected residue with the Cα of the first amino acid ideally intercepted along the direction of motion. On the right, the average distance over time plot (blue line) and its linear fit (dotted line) between each amino acid and its intercept. The color of the fit dotted line matches the distances selected on the structural representation. The light blue area dispersed along the plot is the error on the average distance. **(C)** Left: covariance matrix calculated for equilibrium MD trajectories; center: covariance matrix for D-NEMD at 20 ns after the perturbation. Right: the plot of the first Principal Components derived from these covariance matrices.

FIGURE S11

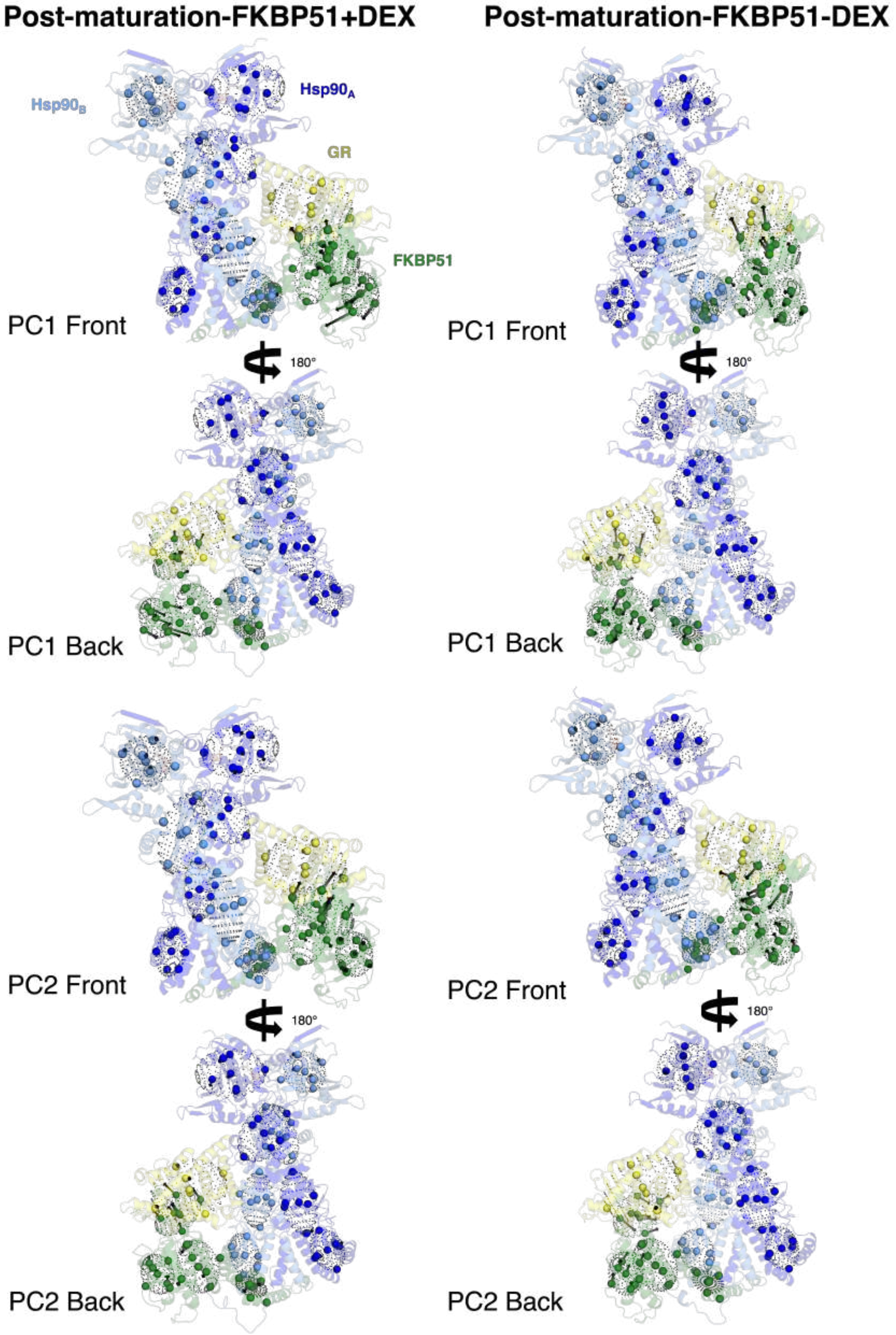

Figure S11: Projection of the first two PCA eigenvectors (vectors as arrows) onto structures of the Post-maturation-FKBP51 complex, from unperturbed (Post-maturation-FKBP51+DEX; left) and perturbed simulations (Post-maturation-FKBP51-DEX; right). Spheres represent the TOG-derived reduced points, while dots outline the shape of the identified ellipsoids that include each cluster of spheres. For each PC, the front and rear views are provided.

FIGURE S12

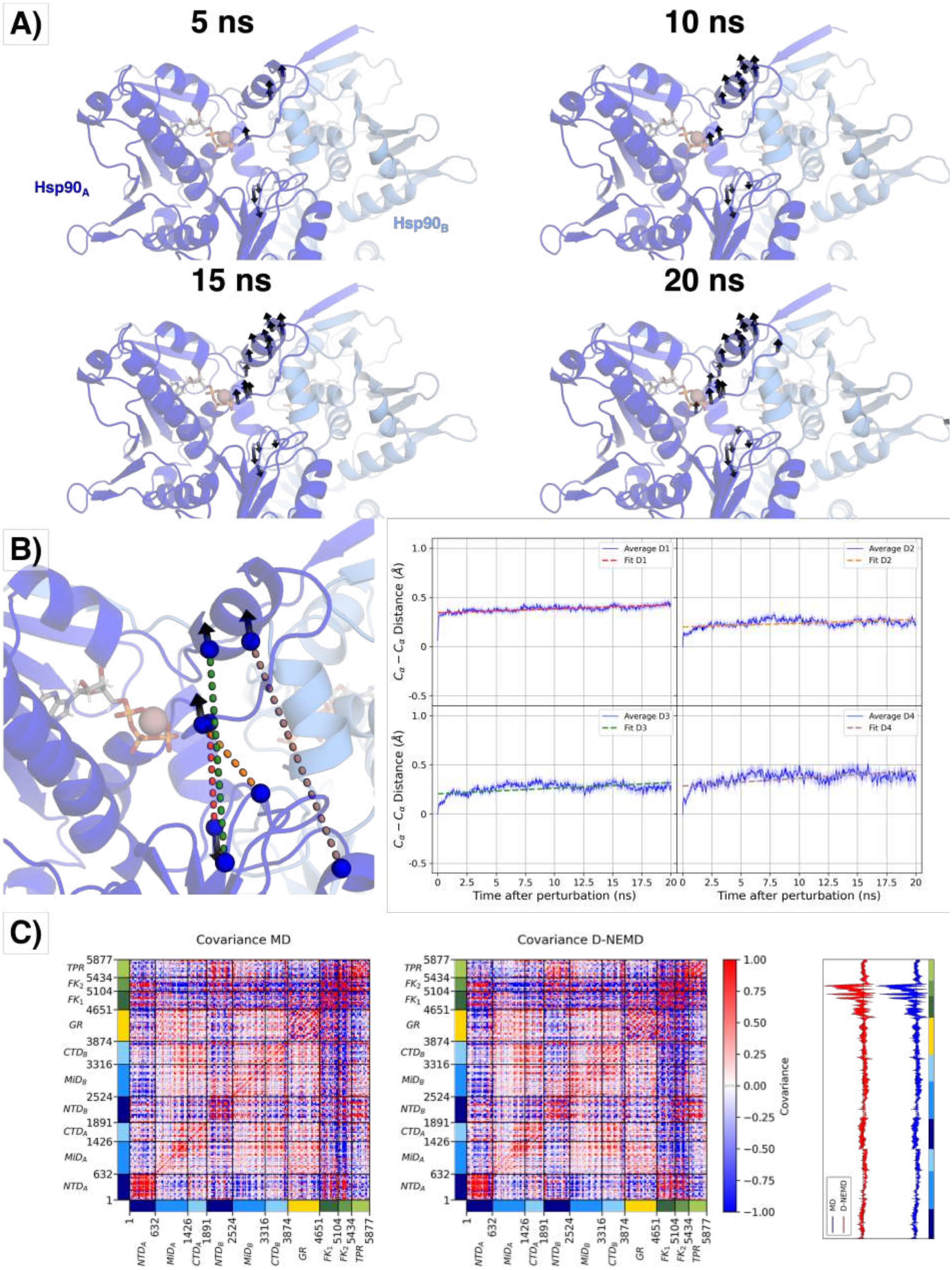

Figure S12: Internal D-NEMD analysis results for the Post-maturation-FKBP51+DEX complex ATP hydrolysis within protomer A conditions. **(A)** The evolution over time of the identified residue displacements. The arrows indicate the average direction and extent of the captured motion. **(B)** On

the left, four of the relevant residue movements are selected and represented with arrows as in panel A. The dotted lines connect each affected residue with the relevant amino acids along the direction of the motion. On the right, the average distance over time plot (blue line) and its linear fit (dotted line). The color of the fit dotted line matches the distances selected on the structural representation. The light blue area dispersed along the plot is the error on the average distance. **(C)** On the left, the maps for the covariance matrices calculated for equilibrium trajectories and D-NEMD at 20 ns after the perturbation. On the right, the plot of the first eigenvector components.

FIGURE S13

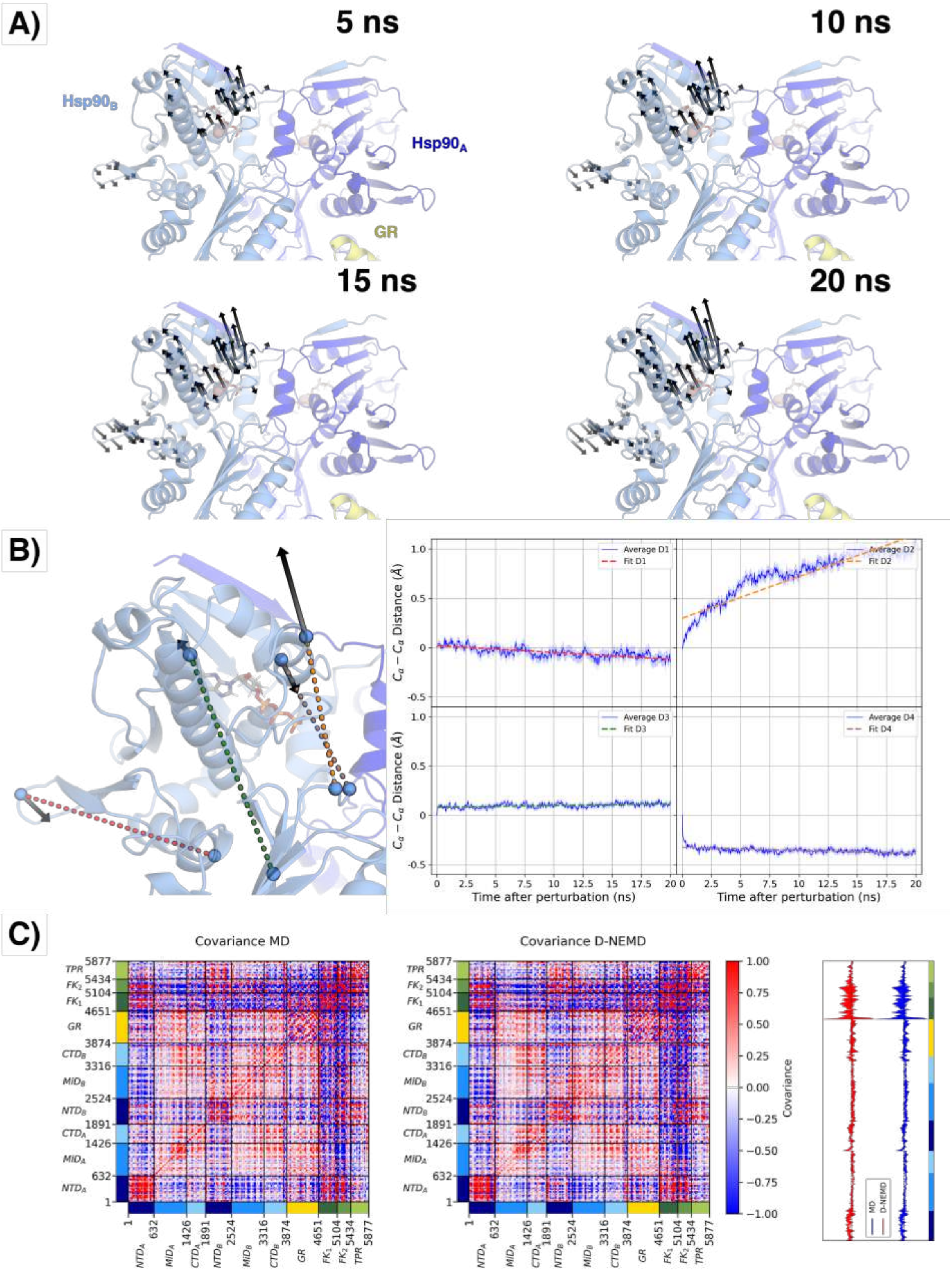

Figure S13: Internal D-NEMD analysis results for the Post-maturation-FKBP51+DEX complex ATP hydrolysis within protomer B conditions. **(A)** The evolution over time of the identified residue displacements. The arrows indicate the average direction and extent of the captured motion. **(B)** On

the left, four of the relevant residue movements are selected and represented with arrows as in panel A. The dotted lines connect each affected residue with the relevant amino acids along the direction of the motion. On the right, the average distance over time plot (blue line) and its linear fit (dotted line). The color of the fit dotted line matches the distances selected on the structural representation. The light blue area dispersed along the plot is the error on the average distance. **(C)** On the left, the maps for the covariance matrices calculated for equilibrium trajectories and D-NEMD at 20 ns after the perturbation. On the right, the plot of the first eigenvector components.

FIGURE S14

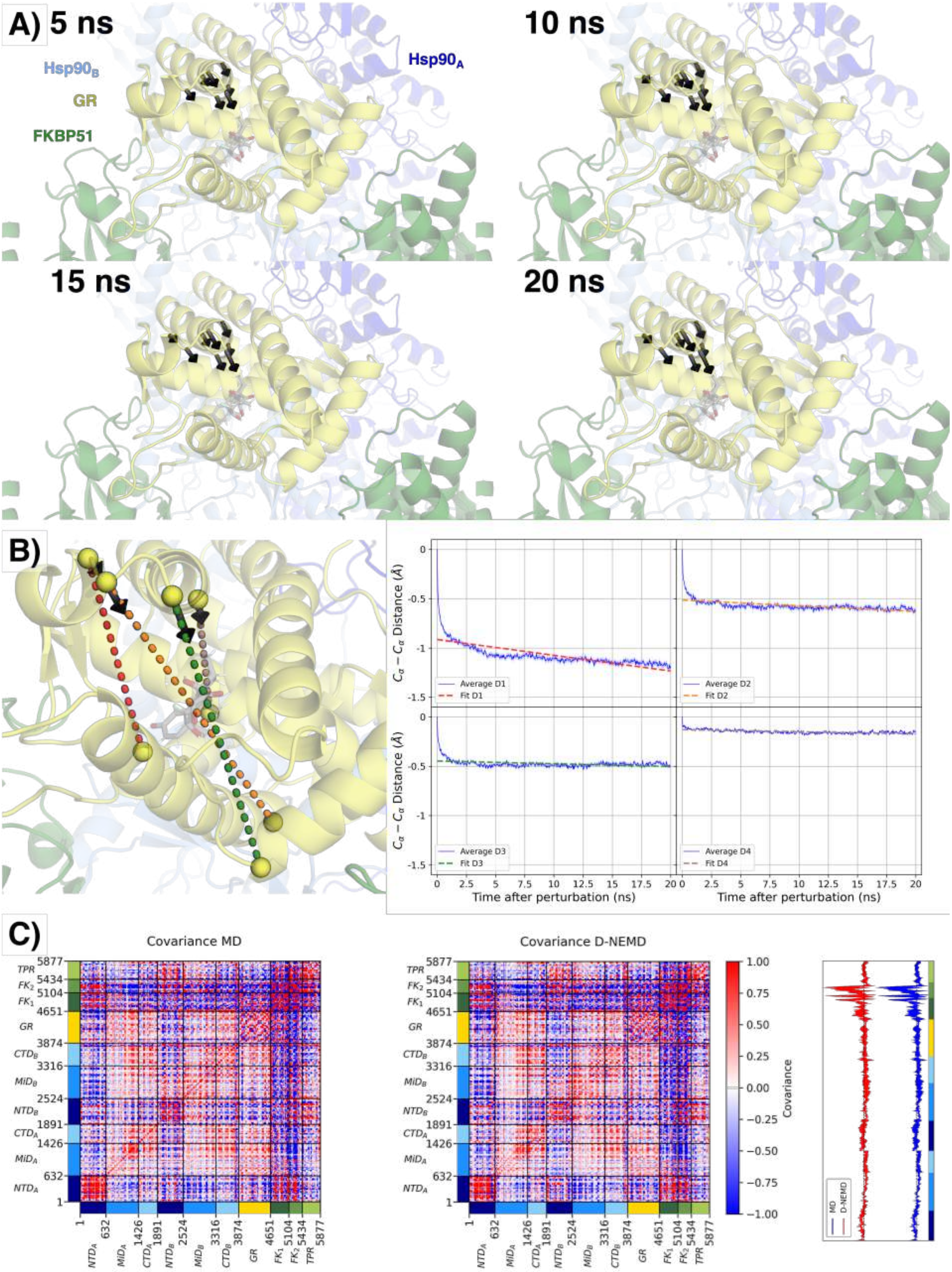

Figure S14: Internal D-NEMD analysis results for the Post-maturation-FKBP51+DEX complex upon DEX removal from GR. **(A)** The evolution over time of the identified residue displacements. The arrows indicate the average direction and extent of the captured motion. **(B)** On the left, four of

the relevant residue movements are selected and represented with arrows as in panel A. The dotted lines connect each affected residue with the relevant amino acids along the direction of the motion. On the right, the average distance over time plot (blue line) and its linear fit (dotted line). The color of the fit dotted line matches the distances selected on the structural representation. The light blue area dispersed along the plot is the error on the average distance. **(C)** On the left, the maps for the covariance matrices calculated for equilibrium trajectories and D-NEMD at 20 ns after the perturbation. On the right, the plot of the first eigenvector components.

FIGURE S15

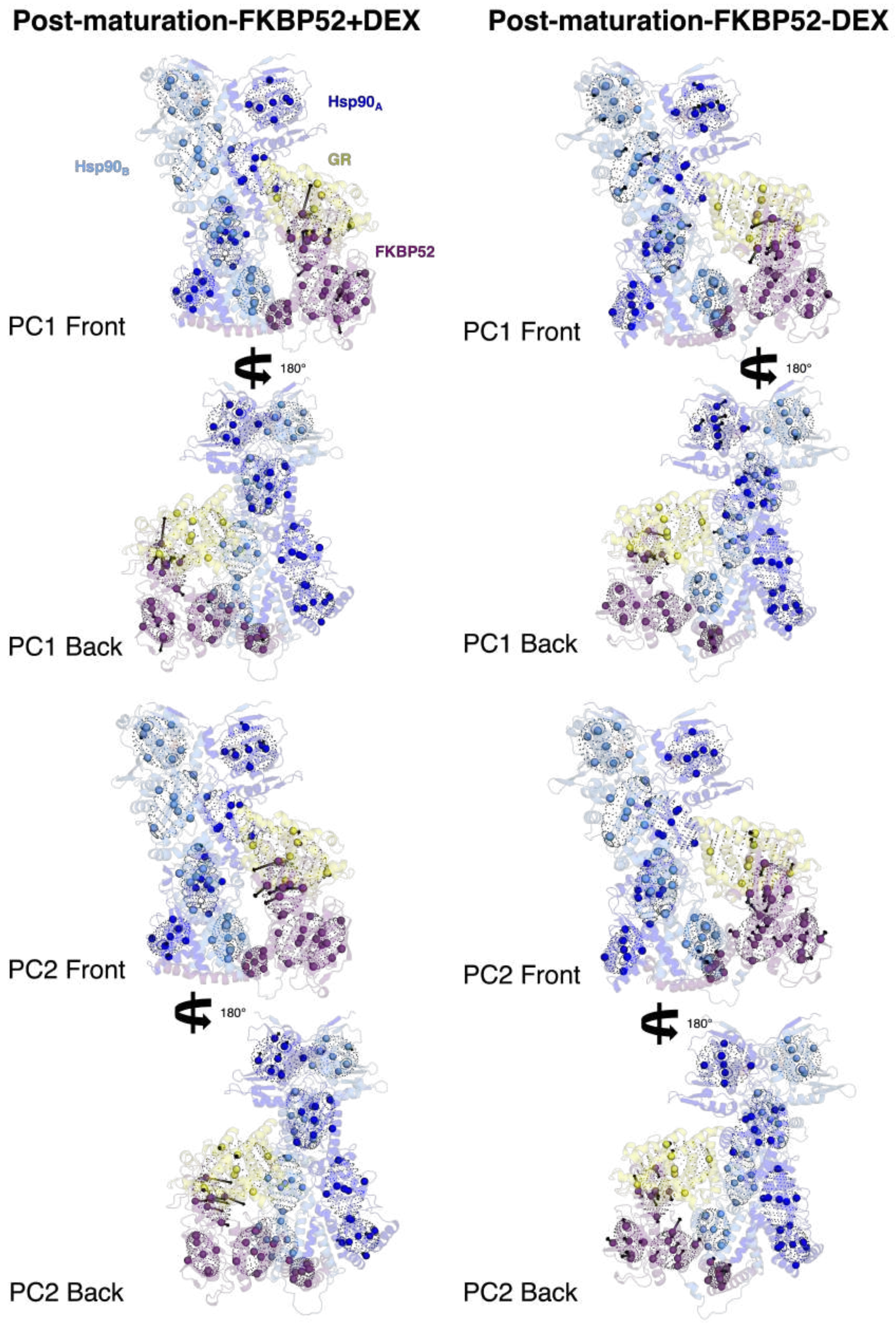

Figure S15: Projection of the first two PCA eigenvectors (vectors as arrows) onto structures of the Post-maturation-FKBP52 complex, from unperturbed (Post-maturation-FKBP52+DEX; left) and perturbed simulations (Post-maturation-FKBP52-DEX; right). Spheres represent the TOG-derived reduced points, while dots outline the shape of the identified ellipsoids that include each cluster of spheres. For each PC, the front and rear views are provided.

FIGURE S16

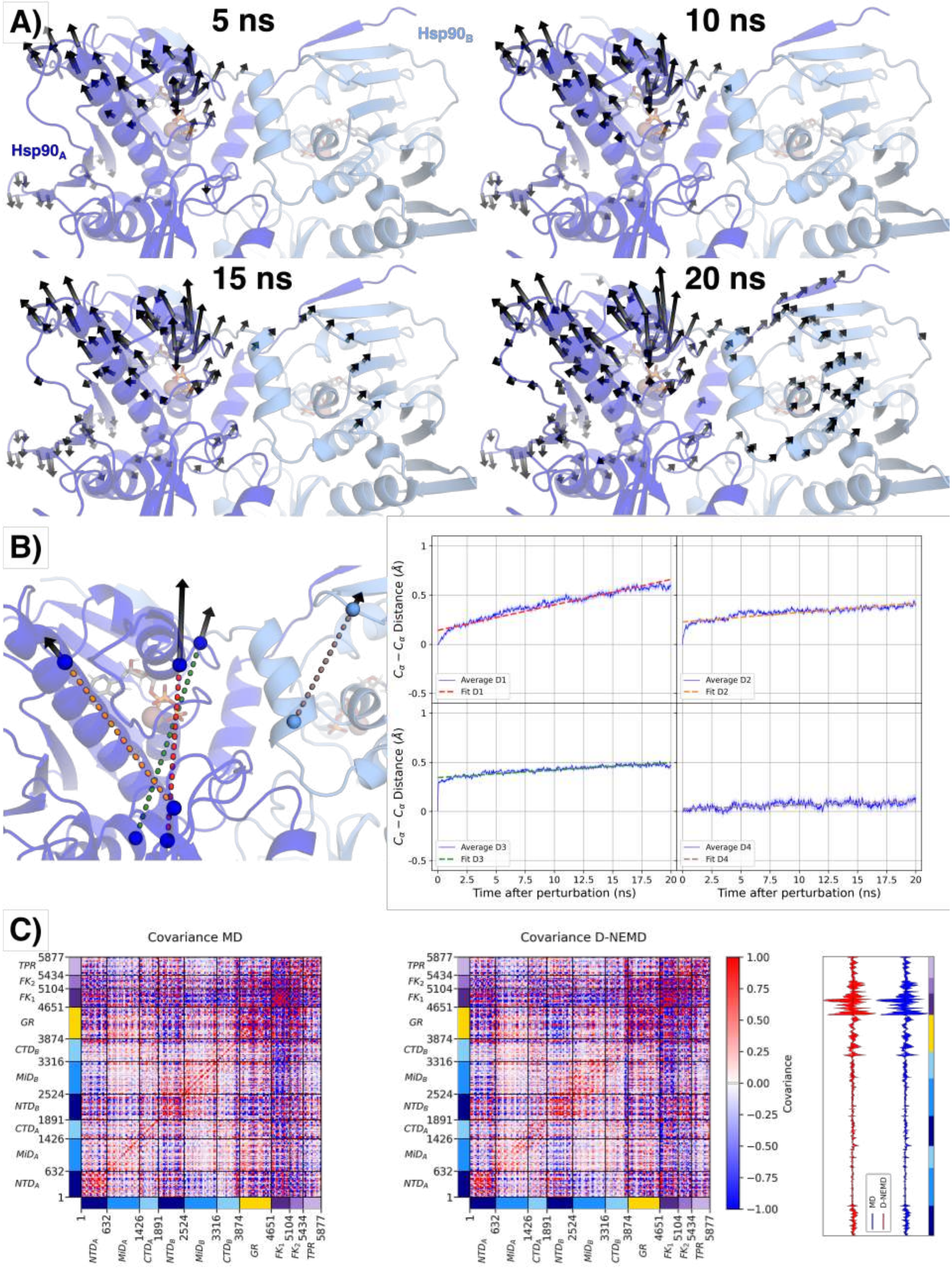

Figure S16: Internal D-NEMD analysis results for the Post-maturation-FKBP52+DEX complex ATP hydrolysis within protomer B conditions. **(A)** The evolution over time of the identified residue displacements. The arrows indicate the average direction and extent of the captured motion. **(B)** On

the left, four of the relevant residue movements are selected and represented with arrows as in panel A. The dotted lines connect each affected residue with the relevant amino acids along the direction of the motion. On the right, the average distance over time plot (blue line) and its linear fit (dotted line). The color of the fit dotted line matches the distances selected on the structural representation. The light blue area dispersed along the plot is the error on the average distance. **(C)** On the left, the maps for the covariance matrices calculated for equilibrium trajectories and D-NEMD at 20 ns after the perturbation. On the right, the plot of the first eigenvector components.

FIGURE S17

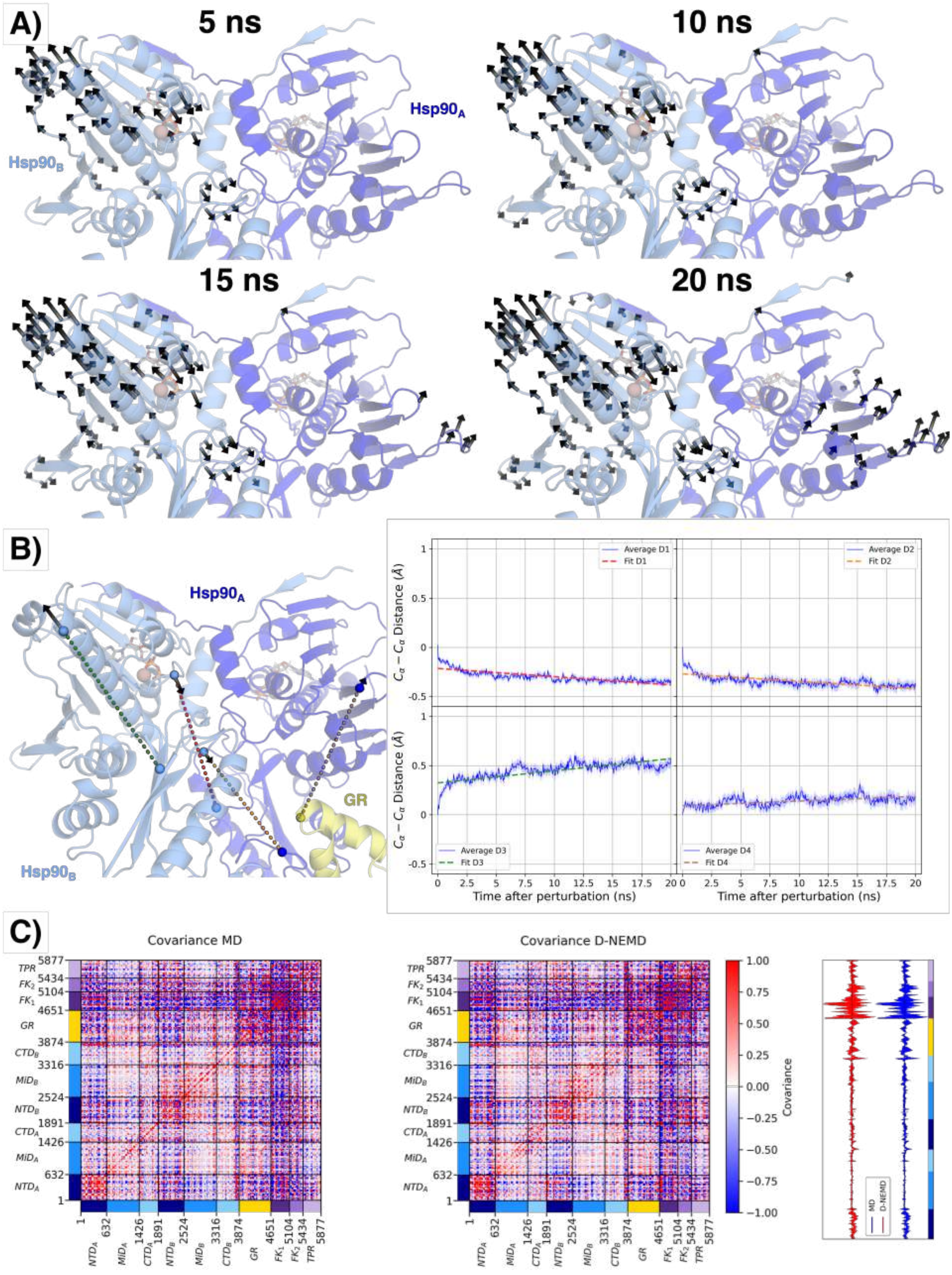

Figure S17: Internal D-NEMD analysis results for the Post-maturation-FKBP52+DEX complex ATP hydrolysis within protomer B conditions. **(A)** The evolution over time of the identified residue displacements. The arrows indicate the average direction and extent of the captured motion. **(B)** On

the left, four of the relevant residue movements are selected and represented with arrows as in panel A. The dotted lines connect each affected residue with the relevant amino acids along the direction of the motion. On the right, the average distance over time plot (blue line) and its linear fit (dotted line). The color of the fit dotted line matches the distances selected on the structural representation. The light blue area dispersed along the plot is the error on the average distance. **(C)** On the left, the maps for the covariance matrices calculated for equilibrium trajectories and D-NEMD at 20 ns after the perturbation. On the right, the plot of the first eigenvector components.

FIGURE S18

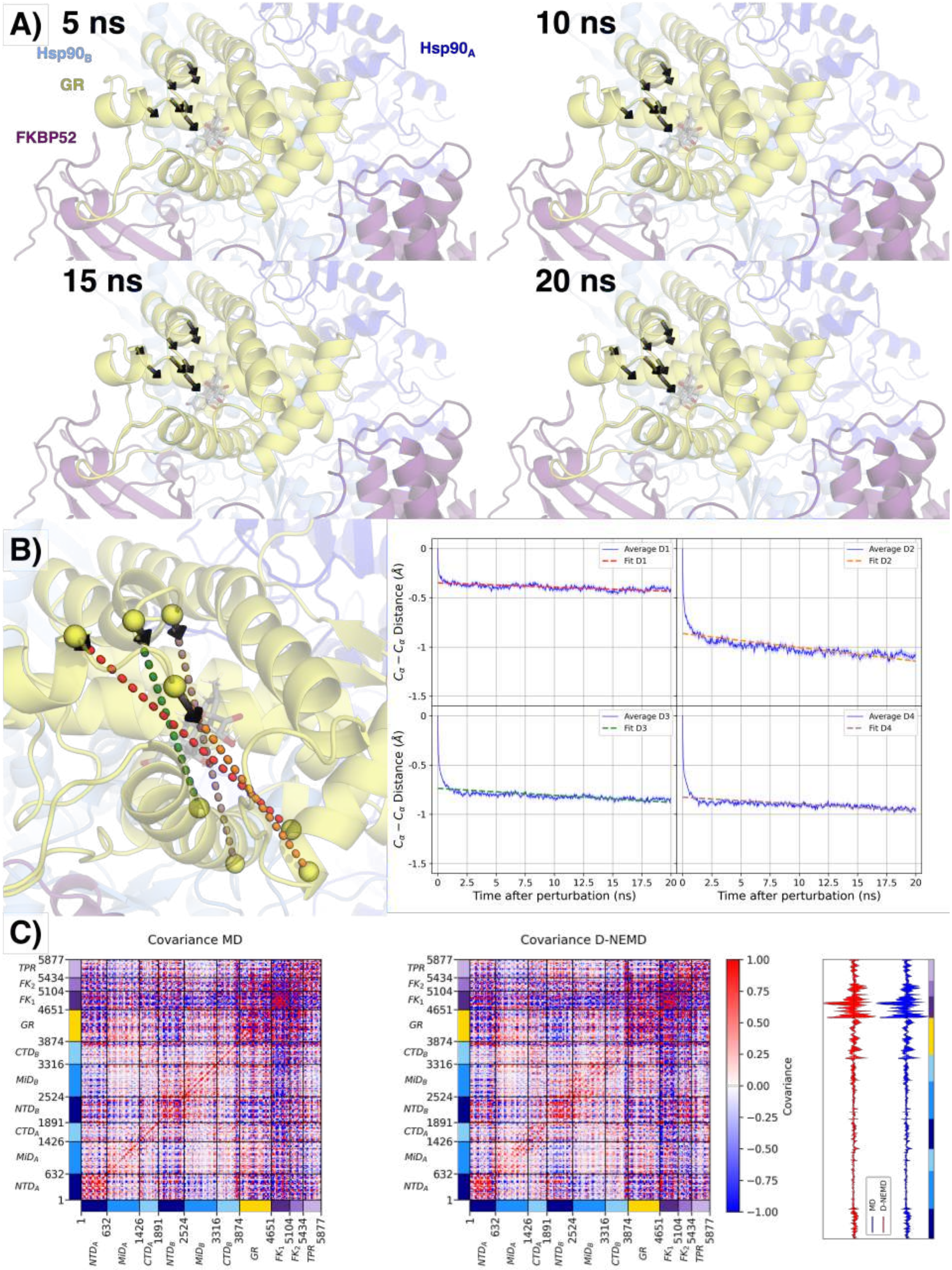

Figure S18: Internal D-NEMD analysis results for the Post-maturation-FKBP52+DEX upon DEX removal from GR. **(A)** The evolution over time of the identified residue displacements. The arrows indicate the average direction and extent of the captured motion. **(B)** On the left, four of the relevant

residue movements are selected and represented with arrows as in panel A. The dotted lines connect each affected residue with the relevant amino acids along the direction of the motion. On the right, the average distance over time plot (blue line) and its linear fit (dotted line). The color of the fit dotted line matches the distances selected on the structural representation. The light blue area dispersed along the plot is the error on the average distance. **(C)** On the left, the maps for the covariance matrices calculated for equilibrium trajectories and D-NEMD at 20 ns after the perturbation. On the right, the plot of the first eigenvector components.

FIGURE S19

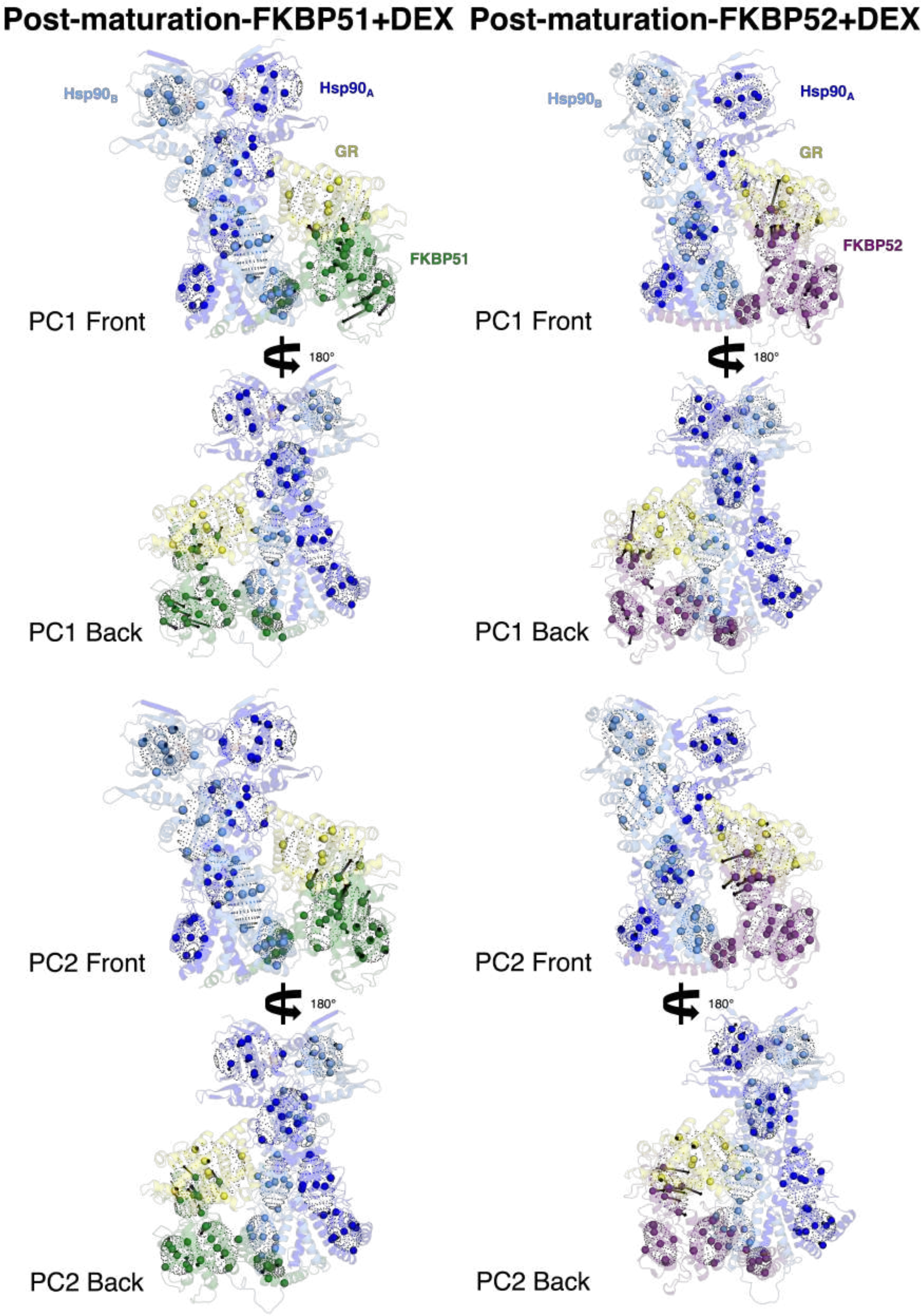

Figure S19: Projection of the first two PCA eigenvectors (vectors as arrows) onto the relative structures of the immunophilin complex, from Post-maturation-FKBP51+DEX (left) and Post-maturation-FKBP52+DEX (right) simulations. Spheres represent the TOG-derived reduced points, while dots outline the shape of the identified ellipsoids that include each cluster of spheres. For each PC, the front and rear views are provided.

FIGURE S20

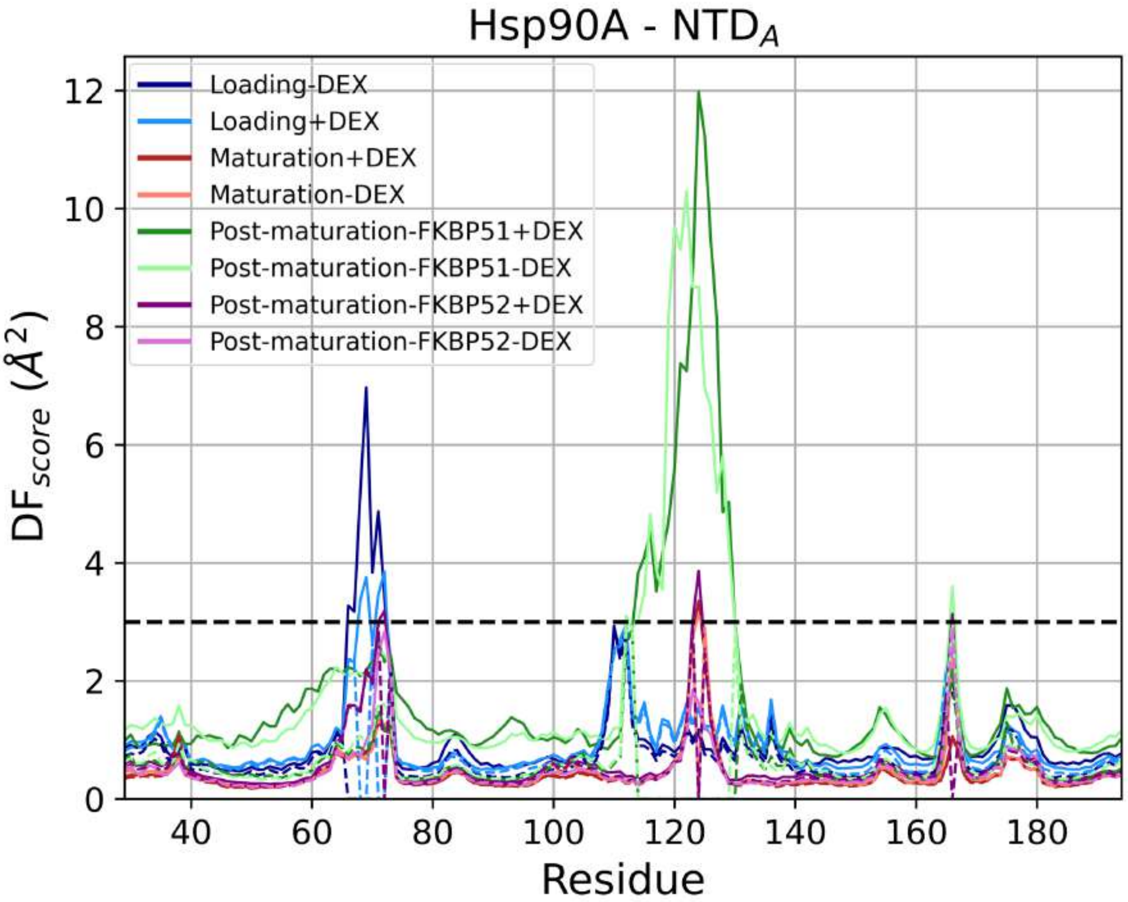

Figure S20: The DF score plot of the NTD$_A$ for all the simulated systems. The original DF score is represented by colored solid lines. The black dotted line represents the threshold to iteratively discard highly flexible amino acids. Colored dotted lines represent the DF score after the iterative selection termination. This plot is also shown in Figure 3B.

FIGURE S21

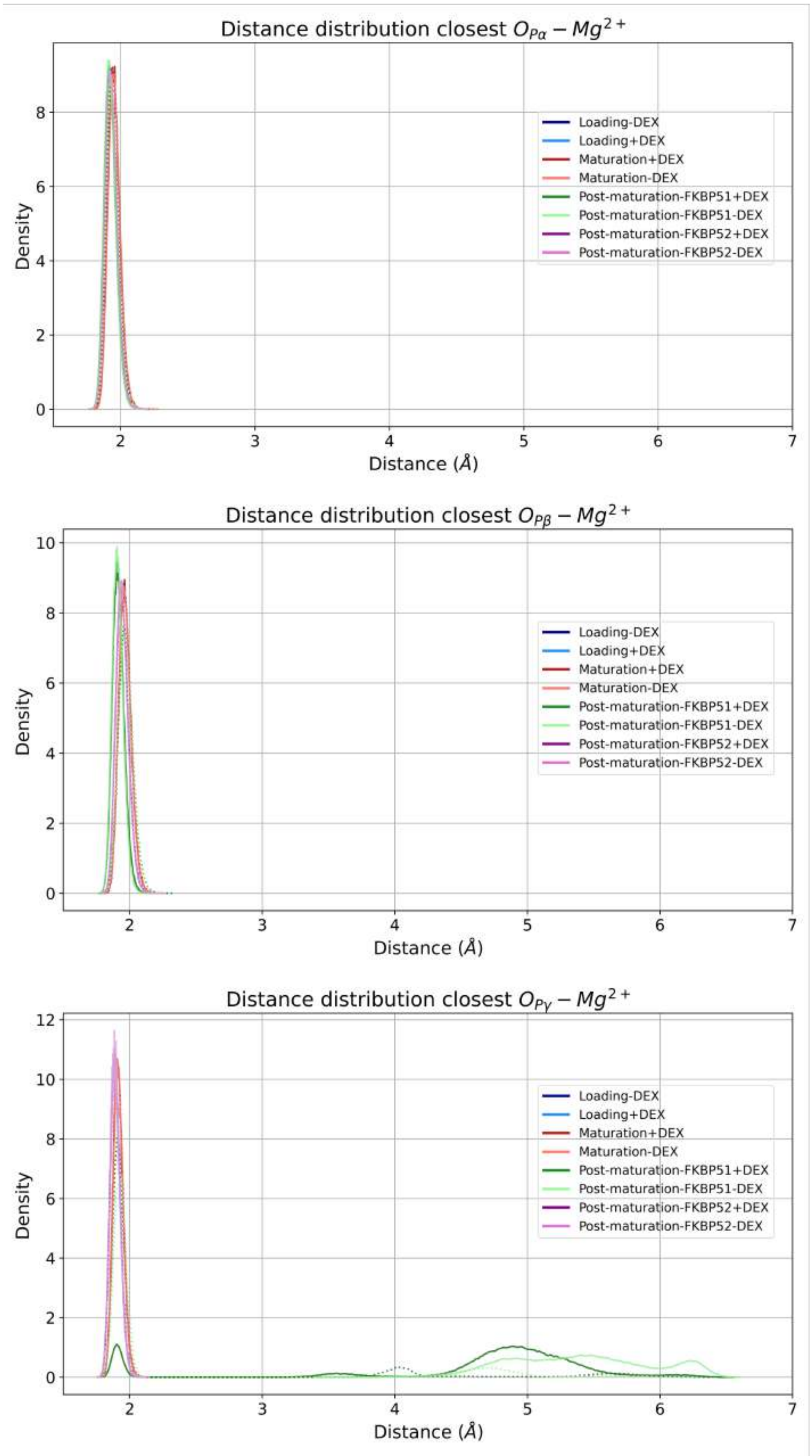

Figure S21: The distance distribution plots between the closest to magnesium oxygen of each phosphate and the Mg^{2+} ion during the simulation for each model.
